# Predator avoidance behaviour in green tortoise beetles depends on environment and shield ontogeny, but varies over time and space

**DOI:** 10.1007/s00442-026-06005-1

**Published:** 2026-11-11

**Authors:** Erica Winslott, Kristina Karlsson Green, Maja Tarka

**Affiliations:** 1 Department of Biology, Lund University, Ekologihuset, Naturvetarvägen 6A, 223 62 Lund, Sweden; 2 Department of Plant Protection Biology, Swedish University of Agricultural Sciences, Box 190, 234 22 Lomma, Sweden

**Keywords:** Ontogenetic differences, Intraspecific variation, Multiple anti-predator defences, Temporal variation, Spatial variation

## Abstract

A prey species can have multiple predator defence mechanisms and these may be used in context-dependent ways to increase survival. How predator avoidance behaviours vary within a species, and how this variation is maintained, are important ecological and evolutionary questions where factors such as the physical state of the individual or the environmental setting may play a role. We investigated spatial and temporal intraspecific variation in two anti-predator behaviours in the green tortoise beetle *Cassida viridis*: ducking under their shield (expanded pronotum and elytra) or dropping from their host plants, by simulating a predator attack in the beetles’ natural environment. We found that the beetle ontogeny (measured as shield hardness and maturity) and the substrate underneath the host plant (water or land) influenced the anti-predator response in *C. viridis*. Immature, soft-shielded beetles were more likely to drop than duck, and mature beetles with a hard shield preferred to duck, whereas immature beetles with a hard shield did not show a preference. Beetles situated on host plants above water ducked more, but no preference for either behaviour was found for beetles situated above land. These results indicate a cost-benefit balance in the response of the anti-predator defences in our study populations of *C. viridis*. In addition, the effect of ontogeny varied between populations and years, and a tendency for such variation was also found for substrate. This study emphasises the importance of considering several factors and their interactions to understand the ecology, maintenance, and evolution of multiple anti-predator behaviours.

**Supplementary Information:**

The online version contains supplementary material available at https://doi.org/10.1007/s00442-026-06005-1.

## Introduction

Predation is a strong selective force that shapes adaptive anti-predator behaviours (Vitt et al. 2002; Losos et al. 2004; Zhou et al. 2017; Sugiura 2020; Lima and Dill 1990) and defensive morphology (Mikolajewski and Rolff 2004; Shinohara and Takami 2020). There is a wide diversity in the defence mechanisms that prey species have evolved against predators (Ruxton et al. 2018), and a prey species can even possess multiple defence mechanisms, which may be used simultaneously or alternatively during a predator attack (Vitt et al. 2002; Exnerová et al. 2023; Kikuchi et al. 2023; Riley et al. 2023). For example, prey may exhibit both a morphological defence and an escape behaviour, as seen in Hermann’s tortoises (*Testudo hermanni*), which either hide within their shells or flee from predators (Golubović 2015). The evolution of multiple defence mechanisms instead of one super-defence system is intriguing and remains a major question in evolutionary biology (Exnerová et al. 2023). Each defence mechanism is costly and may require special morphological structures, colouration, metabolites, or incur indirect ecological costs such as decreased competitive ability or missed mating opportunities (Bowers 1992; Ruxton et al. 2018). Yet, many species exhibit multiple defence mechanisms and little is known about the origins of this variation.

In prey species that possess multiple anti-predator defences, the defence mechanism used during a predator attack may depend on, e.g., the abiotic and biotic environment (Losey and Denno 1998; Shine et al. 2000; Vitt et al. 2002; Rojas et al. 2017; Zvereva et al. 2018) or individual-level variation, such as ontogeny (e.g. Losey and Denno 1998; Landová et al. 2013; Golubović 2015; Raška et al. 2023), as the anti-predator strategies may shift during an individual’s life. For example, the lizard *Lacerta perspicillata* uses different escape routes depending on its position in relation to hiding places and the direction of the predator attack (Vitt et al. 2002), and the shift in antipredation behaviour (aggression or fleeing) in juvenile and adult racer snakes *Coluber constrictor* is correlated with the ontogenetic change in colour pattern (Creer 2005).

Considering the effects of environmental factors and ontogenetic stages on anti-predator responses, we may also expect variation in morphological, physiological, behavioural, life history and anti-predator traits among populations or across years. Such natural, intraspecific variation across time and space is relevant to address for understanding the evolution and maintenance of multiple anti-predator defences. To our knowledge, however, there have only been a few studies addressing variation in predation avoidance traits between populations (Magurran 1999; Creel et al. 2008; Ortega et al. 2014; Golubović 2015; Golubović et al. 2017, 2021) or across years (Ferree et al. 2018), and none that have considered both spatial and temporal variation in the same study. The studies that are present (Magurran 1999; Creel et al. 2008; Ortega et al. 2014; Golubović 2015; Golubović et al. 2017, 2021; Ferree et al. 2018) indicate that both variation and consistency in anti-predator behaviours exist across populations or time. There is thus a need for studies investigating both population-level and year-to-year differences to increase our understanding of the presence or absence of such variation and the conditions under which it occurs (Exnerová et al. 2023).

To further our understanding of how multiple anti-predator strategies vary across space and time, we experimentally investigated anti-predator behaviours in three free-ranging populations of the green tortoise beetle *Cassida viridis* across two years. *Cassida viridis* is a phytophagous beetle that alternates between two distinct behaviours in response to predator attacks: dropping or ducking behaviour.

Dropping, in our definition when an animal falls headlong from a higher position to a lower one, is a simple yet effective behavioural predator avoidance strategy commonly used across the animal kingdom (Francke et al. 2008; Humphreys and Ruxton 2019, *lizards*: Vitt et al. 2002, *birds*: Lima 1993; Hedenström and Rosén 2001, *spiders*: Blackledge and Pickett 2000, *butterflies*: Perović et al. 2008; Castellanos and Barbosa 2011; Zhou et al. 2017*and aphids*: Gish and Inbar 2006). To drop into a new, unknown area can be costly as it dislocates the individual from a food source or potential mates, touchdown might happen on a dangerous substrate, and there is a risk of encountering other predators (Humphreys and Ruxton 2019). Dropping behaviour in insects has mostly been studied in pea aphids (Muller 1983; Dill et al. 1990; Losey and Denno 1998; Agabiti et al. 2016), but has also been reported in several beetle species (Crowson 1981; Selman 1988; Jolivet and Verma 2002; Matsubara and Sugiura 2018, 2021), specifically within Cassidinae, the subfamily to which *C. viridis* belongs (Olmstead 1996; Jolivet and Verma 2002).

When ducking, tortoise beetles retract their legs and antennae underneath their tortoise-like ‘shield’ consisting of their expanded pronota and elytra (Windsor et al. 1992; Jolivet and Verma 2002), and they hold on firmly to the substrate with the pads and claws on their tarsi (Engel 1936; Selman 1988; Buzzi 1988; Windsor et al. 1992; Jolivet 1997; Jolivet and Verma 2002). The shield seems to be an efficient defence against predators (Olmstead 1996; Shinohara 2021), such as crab spiders and assassin bugs (Shinohara and Takami 2020; but see Winslott et al. 2026).

The use of either the dropping or ducking response likely depends on a trade-off between predation risk and the costs associated with each avoidance behaviour. One factor that might influence these costs is the ontogeny of the beetle shield. Individuals emerging from the pupae have a soft cuticle that takes several days to sclerotise (Wigglesworth V.B. 1939; Pryor 1940; Follett and Hilbeck 1995; Scalet et al. 2022; Winslott et al. unpublished). The soft shield might be penetrable by predators, suggesting that the ducking behaviour is less effective and could therefore be used to a lower degree during this life stage. After the soft shield stage (hereafter soft-immature stage), the sclerotised hard shield of *C. viridis* stays unchanged for 1–2 weeks (hereafter hard-immature stage). During the final stage of the shield ontogeny, a silver line appears along the anterior margin of the elytra, which seems to indicate maturation (Engel 1936) (hereafter hard-mature stage). We predict that during the hard-immature phase, the beetles will be prone to use the ducking behaviour to a greater extent due to the protective properties of the shield and the high costs of the dropping behaviour. We have the same prediction for the hard-mature beetles, although these might be even more prone to duck, as the cost to drop from the host plant during mating season might be even higher.

Furthermore, the behavioural response of *C. viridis* may also be affected by environmental factors, which could be interacting with the ontogeny of the shield. For example, host plant characteristics such as height (Matsubara and Sugiura 2021) and leaf shape (Matsubara and Sugiura 2018) have been shown to affect the dropping behaviour of leaf beetles. Another environmental factor that may affect the response is the substrate beneath the host plants. The host plants of *C. viridis* – different species within the Lamiaceae family (Engel 1936; Jolivet and Verma 2002; Borowiec and Świętojańska 2024) – often grow close to water and are sometimes located directly above water in streams or ponds. Dropping into water might decrease survival, hence assessing the substrate beneath the host plant (using e.g., chemical cues and/or polarized light (e.g. Evans 1988; Mathejczyk and Werenet 2017), can be important for the beetle’s decision to drop or duck. Therefore, we also considered the substrate below the host plant (water or land) as a factor that might affect the anti-predator response of *C. viridis*. We predict that *C. viridis* will use the ducking behaviour more frequently than dropping above water to avoid this substrate.

To increase our understanding of how variation in multiple anti-predator behaviours arises, we tested whether the likelihood of using one or the other predator avoidance behaviour differed depending on the ontogeny (soft-immature, hard-immature and hard-mature), and between beetles situated on host plants above water or land. We did this by simulating a predator attack on beetles in their natural environment. We also tested whether the beetles response of predator avoidance differed between populations, and if the response varied across years. As studies in other systems have shown that traits may, or may not, vary across years or populations (Magurran 1999; Creel et al. 2008; Ortega et al. 2014; Golubović 2015; Golubović et al. 2017, 2021; Ferree et al. 2018), we aimed to explore the potential for spatial and temporal variation in the *Cassida viridis* study system. We did, however, not formulate an a priori prediction; instead, our objective was to test whether variation was present.

## Methods

### Study species

Tortoise beetles Cassidinae, and tribe Cassidini in particular, have previously been studied for multiple defence mechanisms, such as the larval faecal shield (Gomez et al. 1999; Vencl et al. 1999; Eisner and Eisner 2000), maternal guarding (Gomes et al. 2020), feet adhesion by adults (Eisner and Aneshansley 2000) and the expanded margins of the adults’ shield (Shinohara and Takami 2020). *Cassida viridis* is a common tortoise beetle which is widespread in Europe (Schmitt and Rönn 2011) and recently discovered in North America (Marshall and Paiero 2016). It is a phytophagous beetle, utilising species of a few genera within the Lamiaceae family such as *Mentha*, *Lycopus*, *Stachys* and *Galeopsis* (Engel 1936; Jolivet and Verma 2002; Borowiec and Świętojańska 2024). The adults hibernate during the winter months and emerge in spring to early summer, thereafter the mating season takes place, followed by the larval and pupal stages in summer. The new generation of beetles that eclose enters the next winter’s hibernation after a month or two (Engel 1936; Kosior 1975). When the newly eclosed beetles leave the pupae, their shields are very soft, and it takes 3–6 days for it to sclerotize (Winslott et al. unpublished). As a last ontogenetic stage of the shield, a silver line is formed along the anterior margin of the elytra, adjacent to the posterior margin of the pronotum 7–14 days after the shield has hardened (Winslott et al., unpublished). This silver line may be an indication of maturation (Engel 1936). We classified the first ontogenetic shield stage as soft-immature, the second as hard-immature, (because the silver line had not developed yet), and the third as hard-mature.

There are few reports of predator attacks on adult Cassidine beetles compared to the egg, larval and pupal stages (Olmstead 1996; Shinohara 2021). However, predation on adult tortoise beetles by spiders (Heron 1996; Gomes et al. 2020; Winslott et al. 2026), wasps (Shinohara and Takami 2017, 2022), birds (Chittenden 1924; Yeung 1934; Olmstead 1996), true bugs (Eisner and Aneshansley 2000; Gomes et al. 2020), and ants (Eisner and Aneshansley 2000) has been observed.

### Field sites

We conducted field work in three study sites (Häckeberga 55°35’42.3"N 13°25’14.2"E, Björkelunda 55°33’21.0"N 13°06’52.5"E, Nöbbelöv 55°44’04.5"N 13°09’11.7"E) in Skåne province, southern Sweden. We visited all host plant patches within the study sites one to two times per week from May until the end of September 2022 and from May until the end of August 2023. The host plant patches in Björkelunda and Nöbbelöv are situated around ponds or along the edges of islands within the ponds, whereas the patches in Häckeberga are situated along a stream in an open forest. This creates heterogeneous environments in which some host plants grow above water and others above land. The total host plant patch area was 210 m^2^ in Björkelunda, 65 m^2^ in Nöbbelöv and 200 m^2^ in Häckeberga, of which 60%, 35%, and 80%, respectively, was situated above water. Within each locality, the host-plant community consisted of a mixture of Lamiaceae species, with *Mentha* spp. (*Mentha aquatica* and *Mentha arvensis*) dominating, while *Lycopus europaeus*, *Stachys palustris* and *Galeopsis speciosa* occurred in lower frequencies.

### Field work and behavioural experiment

To find the beetles, we visually scanned the host plants thoroughly from above and the sides, as *C. viridis* usually sits on top of the leaves of their host plants, as many other Cassidinae species (Jolivet and Verma 2002). We used a standardized protocol to assess the predator avoidance response by simulating a non-specific predator attack with a wooden poking stick (Mikolajewski et al. 2006; Eroukhmanoff and Svensson 2009; Harris et al. 2011; Matsubara and Sugiura 2018). This simulation was intended to mimic the physical contact exerted during an attack by a typical vertebrate or arthropod predator of *C. viridis*. We poked the beetles on the rear end of the shield, up to three times with a wooden stick and noted their behaviour. If the beetle dropped, it was assessed as dropping. If the beetle pulled in its legs and antennae underneath the shield and held on to the leaf after the third poke, the behaviour was noted as ducking. During the test, we held either a hand or a Petri dish underneath the leaf that the beetle was positioned on, but without disturbing the leaf. This was to catch the beetle if it dropped. A third avoidance behaviour was observed during the field work, in which the beetles walked away from the attack. We observed the walking behaviour in 138 of 1534 observations. Since the walking and ducking technique makes the individual avoid the substrate below the host plant, and since an individual walking or ducking is likely more protected with a hard shield, compared to a soft one, we merged these two behaviours in the final analysis. Excluding the beetles that utilized the walking behaviour from the analysis gave a similar result (see supplementary tables S1–4).

After the simulated predator attack, we recorded the substrate beneath the host plant (water or land) on which the beetle was found. Although the effect of host plants was not our main focus, we also recorded the plant species on which the beetle was found. Thereafter we assessed the stage of the shield ontogeny. We categorised beetle ontogeny depending on (i) shield hardness and (ii) presence or lack of the elytral silver line. The hardness of the shield cuticle of *C. viridis* was assessed in line with the procedure used by Follett and Hilbeck (1995). It was classified into two categories by pushing with a fingernail on the elytra; if the shield gave way to the push we classified it as a soft shield, but if the cuticle held its shape it was classified as a hard shield. When sclerotized, the cuticle of the *C. viridis* shield is very hard and inflexible, making the distinction between soft and hard shields unambiguous. Depending on whether the silver line was present or not, we categorised the hard-shielded beetles as either hard-immature (without silver line) or hard-mature (with silver line). After the assessment of antipredator behaviour, beetles were individually marked with a permanent marker before being released to ensure that they were not tested again during the study.

### Data analysis

In order to test how the probability of ducking (or dropping) is affected by shield ontogeny and substrate under the host plant, we used a generalized linear model (GLM) with predator avoidance as the response variable (two levels: drop or duck) and ontogeny (factor with three levels: soft-immature, hard-immature, and hard-mature), substrate (factor with two levels: water and land), population (factor with three levels: Björkelunda, Häckeberga, and Nöbbelöv) and year (factor with two levels: 2022 and 2023) as predictor variables. Due to the binary nature of the response variable (dropping or ducking), the model was fitted using a binomial error distribution and a logit link function.

First, we fitted a “***full model***” that included the predator avoidance behaviours as the response variable and all predictor variables (substrate, ontogeny, population, and year) and the interactions between these (*predator avoidance behaviours ~ substrate × ontogeny × population × year*). Next, a **“*best fit model*”** was selected through manual backward elimination to avoid excluding biologically important predictor variables and their interactions which were close to, but did not fall below, the 0.05 threshold of statistical significance. We also validated how the ***“best fit model”*** chosen with manual backward elimination coincided with the model provided by *Akaike information criterion* (AIC) ranking. We used the emmeans package (Lenth 2024) in R to extract probabilities of ducking from the **“*best fit model”*** associated with: (1) substrate underneath host plant (water or land), (2) ontogeny (soft-immature, hard-immature or hard-mature) and (3) population (Björkelunda, Häckeberga, and Nöbbelöv). This method accounts for covariates and other factors in the model; hence providing adjusted probability estimates.

While characteristics of the host plant species that beetles sit on potentially could affect the predator avoidance response in *C. viridis*, we were not able to include host plant species as a factor in the **“*full model”***, because natural variation in host plant distribution and availability makes the sample size too low for some group combinations. We therefore tested how ontogeny, substrate and host plant species affected the anti-predator behaviours in a separate GLM ***“pooled host plant model”*** with pooled populations and years (supplementary figure S2 and tables S18–21). The interpretation of the results from the ***“pooled host plant model”*** could be affected by non-random associations among the explanatory factors ontogeny, host plant species and substrate; e.g., if beetles of different ontogenetic stages differed in their distribution across host plants, or host plants having different occurrence on substrates. We therefore examined if the distribution of ontogenetic stages across host plant species were uneven (supplementary material page 21 and tables S22–23), as well as the distribution of host plant species across substrate (supplementary material page 23 and tables S24–25), keeping population and year data pooled. Additionally, to ensure that our results from the ***“best fit model”*** were not affected by a skewed distribution of beetles of different ontogeny among the substrates, we assessed whether the distribution of individuals with soft-immature, hard-immature or hard-mature ontogeny differed between the substrates (water or land) across populations and years (supplementary material page 26 and tables S26–27).

All analyses were conducted in R statistical software (v4.3.2; R Core Team, 2023).

## Results

A total of 1534 individual beetles’ behaviour was assessed in our three study populations during the field seasons of 2022 and 2023 (Table 1). The proportion of ducking varied slightly between some populations and years (Table 1): Björkelunda 0.61 and 0.63 (2022 and 2023), Häckeberga 0.55 and 0.55, Nöbbelöv 0.51 and 0.40.

**Table 1:** Beetle frequency in the three study populations (Björkelunda, Häckeberga, and Nöbbelöv) separated by year (2022 and 2023, 3rd column), and by year (2022 and 2023) and predator avoidance behaviour (drop and duck) (5th column)

Year	Population	Beetle frequency	Predator avoidance behaviour	Beetle frequency
2022	Björkelunda	407	Drop	160
			Duck	247
2022	Häckeberga	375	Drop	168
			Duck	207
2022	Nöbbelöv	179	Drop	87
			Duck	92
2023	Björkelunda	343	Drop	127
			Duck	216
2023	Häckeberga	173	Drop	78
			Duck	95
2023	Nöbbelöv	57	Drop	34
			Duck	23

**Fig. 1:**
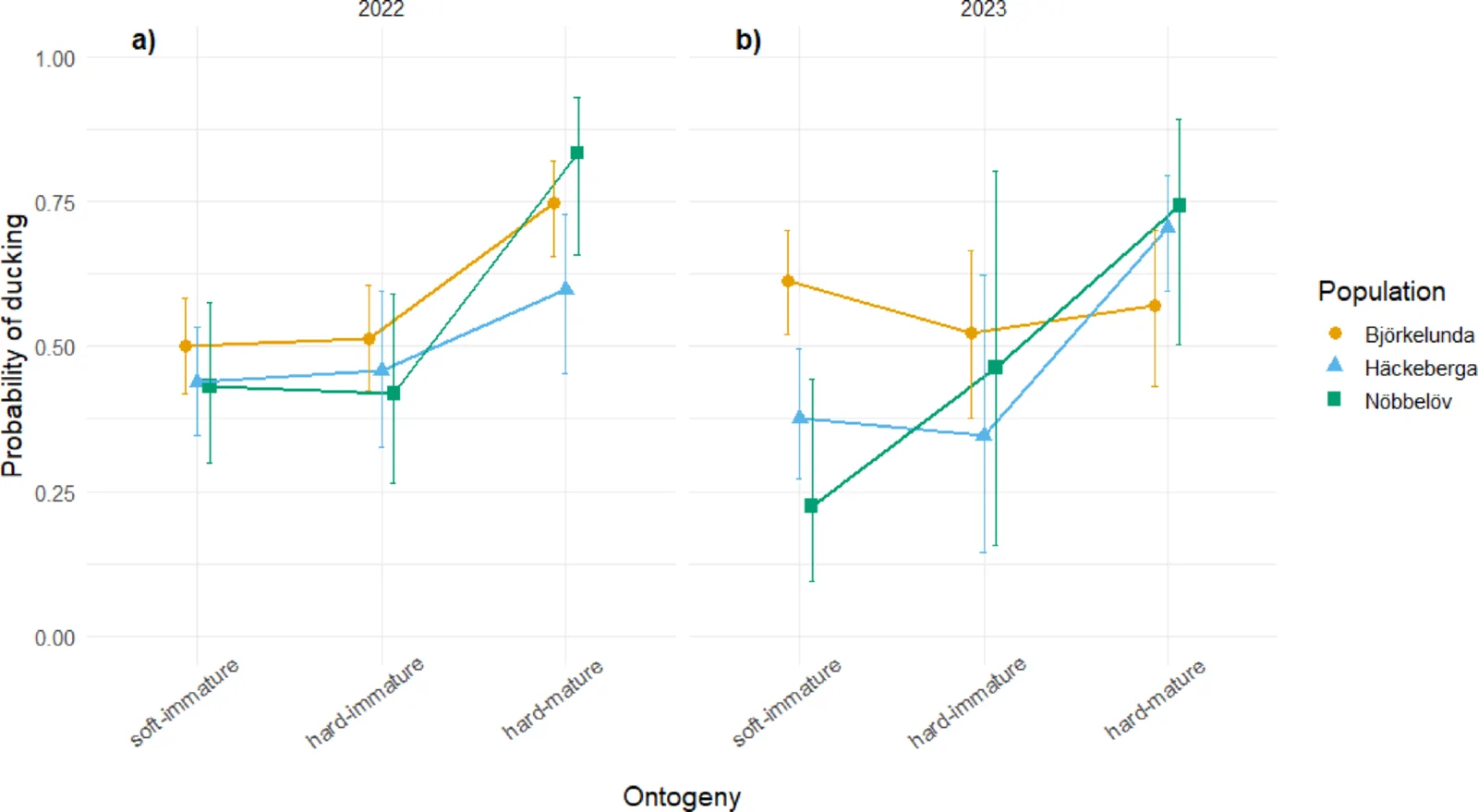
Probability of ducking behaviour in *Cassida viridis* dependent on ontogeny (soft-immature, hard-immature or hard-mature) and year (2022 in panel a and 2023 in panel b). The data are shown for the three study populations (orange filled circle = Björkelunda, blue filled triangle = Häckeberga and green filled square = Nöbbelöv). Points show estimated marginal means from the **“*best fit model*”**; error bars represent 95% confidence intervals calculated using the emmeans package in R

**Fig. 2:**
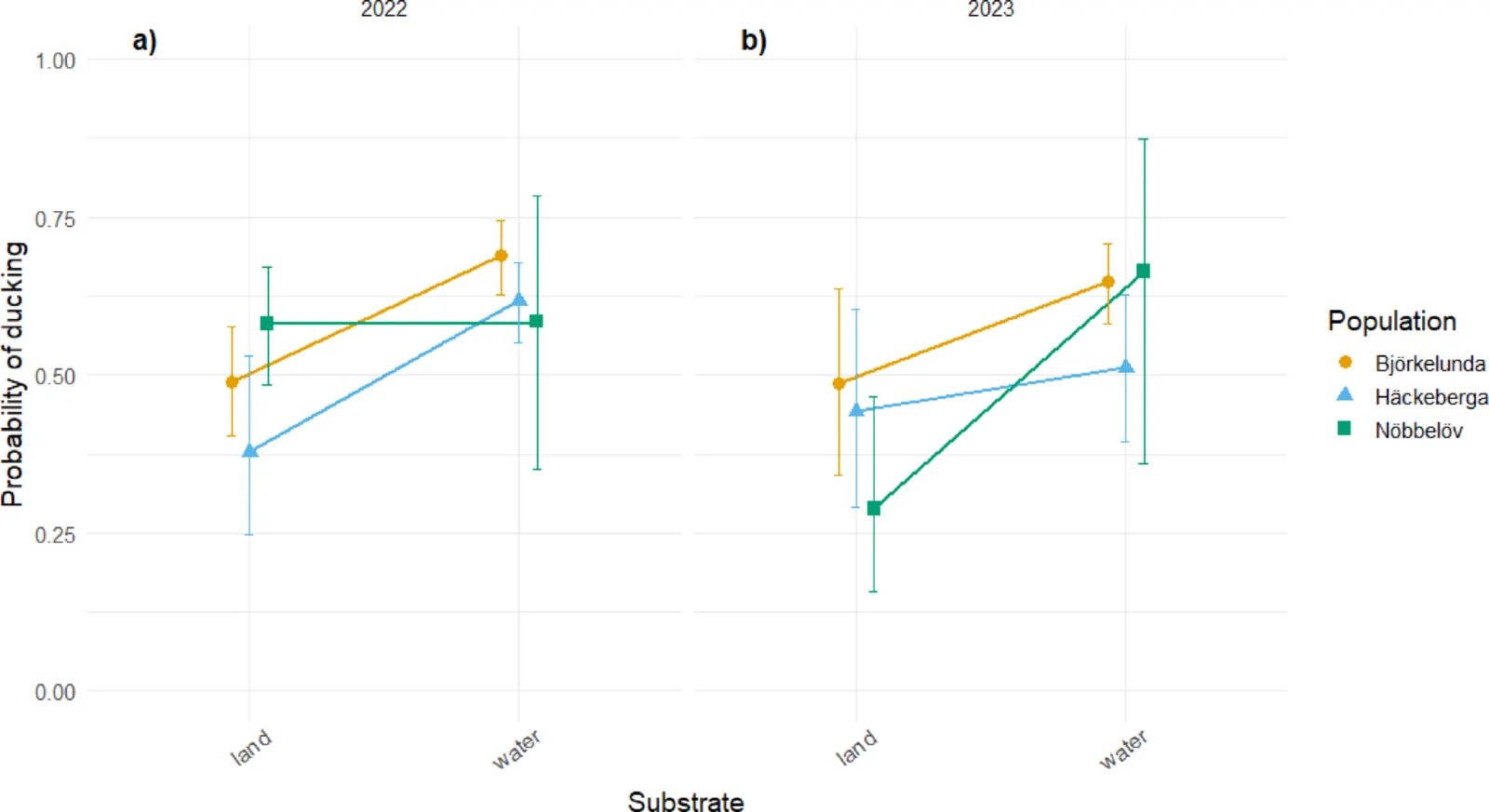
Probability of ducking behaviour in *Cassida viridis* dependent on the substrate under the host plants (land or water) and year (2022 in panel a and 2023 in panel b). The data are illustrated for the three study populations (orange filled circle = Björkelunda, blue filled triangle = Häckeberga and green filled square = Nöbbelöv). Points show estimated marginal means from the **“*best fit model*”;** error bars represent 95% confidence intervals calculated using the emmeans package in R

To establish the **“*best fit model*”** we eliminated the following interactions from the ***“full model”***: “*substrate × ontogeny × population × year*”, *“substrate × ontogeny × population”* and “*substrate × ontogeny × year*” due to their statistically non-significant effect in the model. The ***“best fit model”*** chosen with manual backward elimination gave similar results as the model provided by *Akaike information criterion* (AIC) ranking (supplementary tables S5–6). Based on the **“*best fit model*”**, we found that both main effects and interactions between them affected the predator avoidance response in the green tortoise beetle, *C. viridis.* The anti-predator response was significantly influenced by a three-way interaction between *ontogeny*, *population* and *year* (ꭓ^2^ = 12.58, D.f. = 4, *p* = 0.014). One component of this variation was that the hard-mature beetles in Björkelunda 2022 had significantly higher likelihood of ducking than soft-immature beetles in almost all three populations in 2022 and 2023, except for the soft-immature beetles in Björkelunda 2023 (Fig. 1, supplementary tables S7–8). Additionally, we found a tendency for a three-way interaction between *substrate* underneath host plants, *population* and *year* (ꭓ^2^ = 5.22, D.f. = 2, *p* = 0.073). This tendency could be driven by beetles above water in Björkelunda 2022 having a significantly higher probability to duck, compared to beetles above land in Häckeberga (2022), Björkelunda (2022) and Nöbbelöv (2023), but not compared to Häckeberga (2023), Björkelunda (2023) and Nöbbelöv (2022) (Fig. 2, supplementary tables S9–10).

Using the ***“best fit model”***, we detected a significant two-way interaction between *population* and *year* (ꭓ^2^ = 6.73, D.f. = 2, *p* = 0.035). However, we found no significant differences between groups that could explain this difference (supplementary tables S11–12). In addition, we observed a tendency for a two-way interaction between *ontogeny* and *population* (ꭓ^2^ = 8.67, D.f. = 4, *p* = 0.070). This tendency might be driven by significant differences between a few groups, such as soft-immature beetles in Häckeberga and Nöbbelöv that had a lower probability to duck than hard-mature beetles in all populations, in contrast to soft-immature beetles in Björkelunda that only showed a trend to duck less compared to hard-mature beetles in Nöbbelöv (supplementary tables S13–14). Furthermore, we found a tendency for a two-way interaction between *substrate* and *ontogeny* (ꭓ^2^ = 5.56, D.f. = 2, *p* = 0.062). The tendency was likely influenced by the hard-mature beetles above water that had a significantly higher probability to duck compared to all soft-immature and hard-immature beetles above water and all shield ontogenetic stages above land (supplementary figure S1, and tables S15–16).

**Fig. 3:**
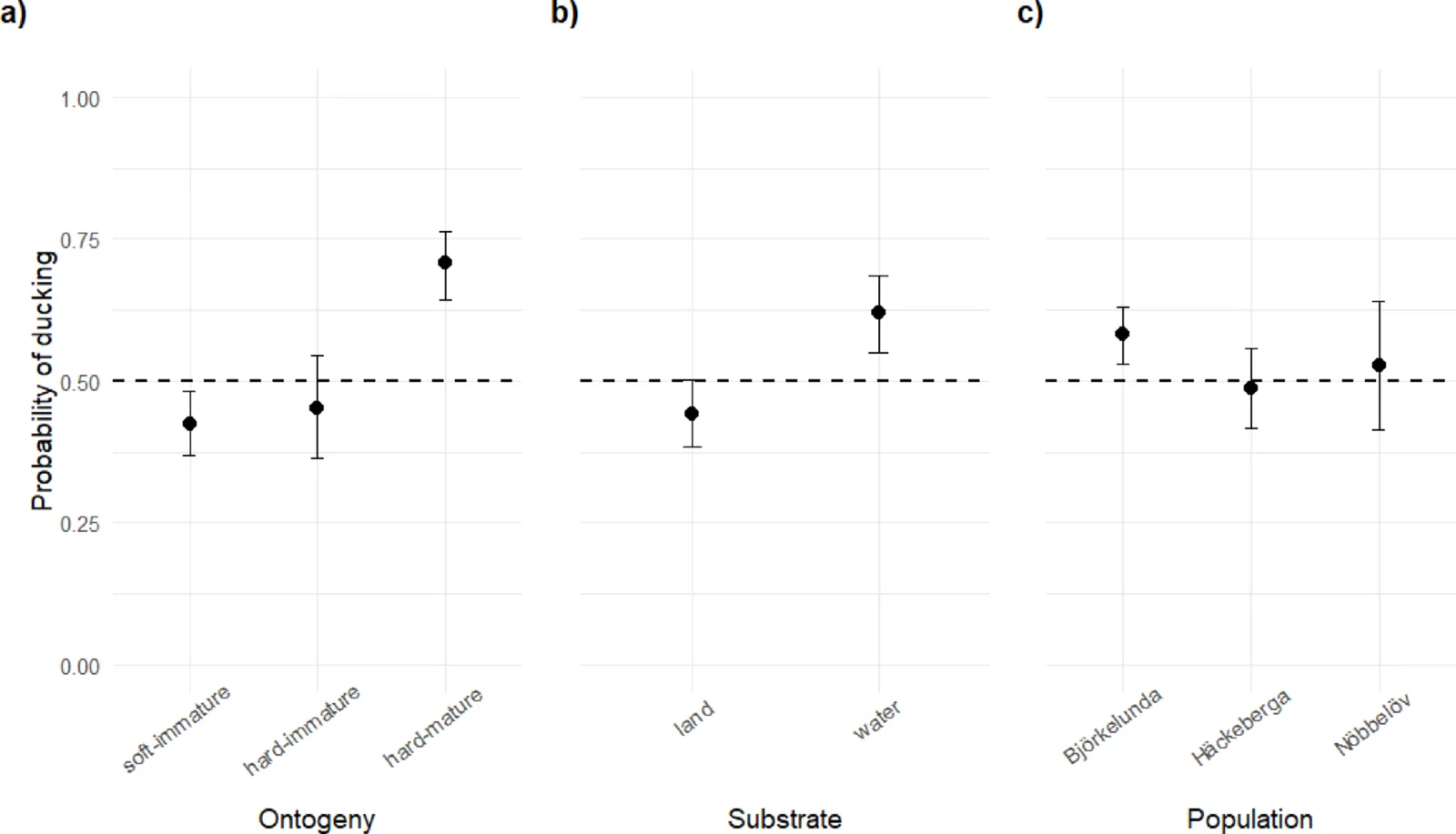
Probability of ducking behaviour in *Cassida viridis* estimated from the ***“best fit model”*** using the emmeans package in R. Points show estimated marginal means of (**a**) ontogeny (adjusted for the effects of substrate, population and year), (**b**) substrate (adjusted for the effects of ontogeny, population and year) and, (**c**) population (adjusted for the effects of ontogeny, substrate and year), error bars represent 95% confidence intervals

The ***“best fit model”*** also showed a strong significant main effect of the ontogeny of the beetles’ shields on the drop and duck behaviour (ꭓ^2^ = 65.83, D.f. = 2, *p* < 0.0001), where soft-immature and hard-immature beetles were less likely to duck than hard-mature beetles (estimate = 1.19, SE = 0.18, z = 6.58, *p* < 0.0001; estimate = − 1.07, SE = 0.23, z = − 4.77, *p* < 0.0001). The adjusted probability to duck (estimated from the ***“best fit model”*** using the emmeans function) for soft-immature individuals (43%, SE = 2.9%) and hard-mature individuals (71%, SE = 3.1%) differed significantly from 50% (where 50% = no preference for either ducking or dropping), while hard-immature beetles (45%, SE = 4.6%) did not differ from 50% (Fig. 3a, supplementary table S17). We also observed a strong main effect of the substrate on the drop or duck behaviour (ꭓ^2^ = 22.87, D.f. = 1, *p* < 0.0001); beetles above water were more likely to duck than beetles above land (estimate = − 0.72, SE = 0.19, z = − 3.85, *p* = 0.0001). The adjusted probability, from the ***“best fit model”***, to duck above water (62%, SE = 3.5%) was significantly different from 50% (where 50% = no preference for either ducking or dropping), but the adjusted probability to duck above land (44%, SE = 3.0%) did not deviate from 50% (Fig. 3b, supplementary table S17). Additionally, we observed a significant main effect of population on the predator avoidance response (ꭓ^2^ = 7.55, D.f. = 2, *p* = 0.023). However, there was no significant difference between populations in how likely they were to duck, but a tendency for such a difference between Björkelunda and Häckeberga (estimate 0.38, SE = 0.18, z = 2.14, *p* = 0.083). The adjusted probability of ducking, provided by the emmeans function from the ***“best fit model”***, was 58% (SE = 2.5%) in Björkelunda, 49% (SE = 3.6%) in Häckeberga and 53% (SE = 5.9%) in Nöbbelöv. These results indicate that beetles in Björkelunda duck in a higher frequency than 50%, while beetles in Häckeberga and Nöbbelöv are equally likely to drop or duck (Fig. 3c, supplementary table S17). Finally, there was no significant main effect of year on the behaviour (ꭓ^2^ = 2.06, D.f. = 1, *p* = 0.151).

To analyse the effects of host plant species on the anti-predator response, we used 1497 individual beetles, of which we had information about ontogeny, substrate and a host plant species. The main host plant species on which *C. viridis* was found were mint (*M. aquaticus* and *M. arvensis*), gypsywort (*L. europaeus*) and marsh woundwort (*S. palustris*). From the ***“pooled host plant model”*** (with pooled data across populations and years); we observed that ontogeny (ꭓ2 = 64.57, D.f. = 2, *p* < 0.0001) and substrate (ꭓ2 = 64.57, D.f. = 2, *p* < 0.0001) had a significant effect on the anti-predator response, while there was a trend for host plant species (ꭓ2 = 5.48, D.f. = 2, *p* = 0.065) to influence the behavioural response of *C. viridis*. No interactions between the factors were significant (supplementary figure S2 and tables S18–21).

Finally, we tested if there were non-random associations among ontogeny, host plant species and substrate which could affect our interpretation of the results. We found that the ontogenetic stages did not deviate from an even distribution across the different host plant species (analysis with pooled population and year data, supplementary material page 21 and tables S22–23). However, we found an uneven distribution of host plant species above water and land, where mint and marsh woundwort were most abundant on water (72% and 86%, respectively), while gypsywort was recorded much less on this substrate (24%) (analysis with pooled population and year data, supplementary material page 23 and tables S24–25). Lastly, there was no significant bias in the distribution of the ontogenetic stages above water and land across populations and years (analysis including all 1534 individuals, supplementary material page 26 and tables S26–27).

## Discussion

In this study, we explored how two anti-predator defences, the dropping and ducking in green tortoise beetles *C. viridis*, are affected by ontogeny and environment, and how the response varies between years and populations. We found that the anti-predator response was affected by both the ontogeny as well as the surrounding environment, specifically the substrate beneath the host plant occupied by the beetle. Furthermore, the predator avoidance response in relation to ontogeny, and possibly to the substrate, varied between and within populations and years, indicating flexibility and intra-specific variation in anti-predator behaviours in time and space.

When exposed to our simulated predator attack, beetles at different ontogenetic stages differed in their likelihood of dropping or ducking (Fig. 3a). As predicted, the hard-mature beetles were more prone to duck than soft-immature beetles and, contrary to our prediction, also than the hard-immature beetles. The soft-immature beetles were more likely to drop, which is in line with our prediction. This may suggest that the beetles with softer shields are not as protected against predator attacks as beetles with hard, more impenetrable shields, and therefore drop even if this could have costs, such as landing onto a dangerous substrate or encountering other predators on the landing site (Humphreys and Ruxton 2019). However, the hard-immature beetles did not duck more than the soft-immature beetles, which indicates that it is not only the hardness of the shield that influences the anti-predator response. In fact, the hard-immature beetles had no significant preference between the two behaviours, and it was only the hard-mature beetles that preferred the ducking behaviour over dropping. This supports that other ontogenetic or life-history factors, such as mating and reproduction, may also influence the anti-predator response. The dropping technique will dislocate an individual from its original position, leading to loss of suitable habitat and food source (Humphreys and Ruxton 2019), therefore, dropping during mating season could pose several additional costs by interrupting mate searching, mate guarding, mating, or oviposition. Hard-mature beetles may thus experience a trade-off between mating opportunity and predator avoidance, which could affect their anti-predator response (see for example, Wing 1988; Franklin et al. 2014). Their greater tendency to duck suggests that they may avoid leaving their hostplant, which also serves as a mating and oviposition site. In contrast, the hard-immature beetles may be more flexible in their anti-predator response. They benefit from the protection of the hard shield, but do not mate before hibernation (Engel 1936), meaning that dropping may be less costly than during mating season, while still, in some contexts, representing the safest strategy. The importance of ontogeny in shaping anti-predator responses has also been observed in other taxonomic groups: snakes (Creer 2005), aphids (Losey and Denno 1998), planthoppers (Kang et al. 2017), geckos (Landová et al. 2013) and tortoises (Golubović 2015), where juveniles have a different strategy than adults. However, the difference in anti-predator responses between mating and non-mating season is less studied. Yet, it has been observed that the root vole (*Microtus oeconomus*) reacts less to least weasel (*Mustela nivalis*) scent during breeding season than during the non-breeding season (Borowski 2002), and male Iberian rock lizards (*L. monticola*) have shorter emergence times from refugees during mating compared to the post-reproductive season (Martín et al. 2003). The necessity to have separate predator avoidance behaviours that are more beneficial at specific life stages could be one reason why multiple anti-predator defences are maintained, even though each of these behaviours requires energy allocation.

The beetles were more likely to duck when they were situated on hostplants above water (Fig. 3b), and this might reflect that dropping into water is more costly. This is also implied by the aphid *A. pisum*, in which individuals from populations with water sources tended to drop less in comparison to populations without water sources around host plants (Muller 1983). The difference in *C. viridis* predator avoidance response above different substrates might indicate that the beetles are able to assess their environment. How they do it is not known, but perhaps they are utilizing similar signals that have been observed in other insect species, such as chemical cues and/or polarized light to locate micro-habitats, such as e.g. water sources (e.g. Evans 1988; Mathejczyk and Werenet 2017). Heterogeneous environments, such as the water and land mosaic of *C. viridis’* habitat, can thereby pose contrasting selective pressures that can lead to evolution of multiple anti-predator behaviours (Templeton and Shriner 2004; Drown et al. 2022). However, an alternative explanation for the behavioural differences observed across substrates could be that hard-mature beetles, which are less likely to drop, occurred more often above water, whereas soft-immature beetles, which are more likely to drop, occurred more often above land. Thus, the apparent substrate effect could partly reflect bias in the distribution of ontogenetic stages, rather than a true difference in anti-predator response. However, we did not find any bias in beetle distribution above substrates that could explain a higher degree of ducking above water (supplementary material page 26 and tables S26-27). Hence, this suggests that ontogeny and substrate are two independent variables which both affect the predator avoidance response in *C. viridis.* Our results, however, indicate that the two factors may interact, since hard-mature beetles situated above water ducked at the highest frequency (supplementary figure S1). The ducking behaviour, which is already the preferred anti-predator response in this ontogenetic group, is thus even more utilised when it is favourable in the environmental context. This combination of ontogeny (hard-mature) and environment (water) affects predator avoidance response in the same direction, resulting in an enhanced ducking response, highlights the importance of investigating several factors that may interact to influence the maintenance of multiple anti-predator behaviours.

Another environmental factor that may influence the anti-predator response is the host plant, e.g., through differences in host plant quality (Dill et al. 1990), leaf morphology (Matsubara and Sugiura 2018) or different predator communities and abundancies (Skirvin and Fenlon 2001; Baker et al. 2012). We, however, only observed a slight difference in predator avoidance behaviour response among our three host plant species, where there was a tendency for beetles on mint to duck to a higher degree. As there was a difference in host plant species distribution above water and land, the higher probability of ducking above water could have been affected by the high distribution of mint above this substrate (supplementary material page 23 and tables S24–25). However, combining the results from all statistical tests (the distribution tests, the ***“best fit model”*** and the ***“pooled host plant model”***), the results suggest that substrate seems to have a stronger influence on anti-predator response than host plant species (supplementary material page 23 and tables S24–25).

The variation in anti-predator response within and between our beetle study populations across years may be explained by several factors (Figs. 1 and 2). One explanation may be fluctuating spatial and temporal selection that maintains multiple anti-predator traits and explain variation across populations and years. In the few previous studies on this topic, variation in anti-predator behaviour between populations (Magurran 1986, 1999; Creel et al. 2008; Ortega et al. 2014; Golubović 2015; Golubović et al. 2017, 2021) and years (Ferree et al. 2018) have been reported. For *C. viridis*, we also observed variation in the predator avoidance behaviours among the study populations and the two years of data collection in relation to ontogeny (Fig. 1), and a trend for variation across populations and years with respect to substrate (Fig. 2). Differences between populations may be due to genetic differences emerging from, for example, local adaptation or genetic drift (e.g. founder effects), but may also result from a plastic response in beetles to environmental factors that we did not record in our study, such as predator community and density as well as predation intensity, which are well-known to affect prey behaviours (Magurran 1986; Losey and Denno 1998; Losos et al. 2004; Creel et al. 2008; Calsbeek and Cox 2010; Deacon et al. 2018; Ferree et al. 2018). Thus, variation in predator presence and predation intensity could be responsible for differences in predator avoidance response between populations (Magurran 1986; Creel et al. 2008) and years (Ferree et al. 2018) even on smaller time scales (Hamilton and Heithaus 2001; Hossie and Murray 2012). Investigating if there is a difference in predation intensity and predator species community among beetle populations, and across years, would thus be highly relevant. Furthermore, it may also be important to assess seasonal variation in predation intensity and predator community, as our ontogenetic beetle categories occur during different months of the year. A better understanding of the natural variation of predation intensity, together with experiments with predator naïve beetles in a common garden setting, may help disentangle whether beetle anti-predator responses are only due to the inherent ontogenetic stage of the beetle or depend on beetles’ previous predator experience. Additionally, to investigate if individual beetles can adjust their defensive behaviour for different contexts, repeated measures of the same beetle individuals covering their adult life cycle, in different environmental settings, could provide valuable insights.

We did find variation in the anti-predator behaviours across ontogenetic stages, environments, populations, and years. Yet, for some populations we did not find any difference between years in the proportion of how the two anti-predator behaviours were utilised (Figs. 1 and 2). Such a lack of variation in traits has also been observed in some previous studies (Magurran 1999; Ortega et al. 2014; Golubović 2015; Golubović et al. 2021). The consistency in frequency of the behaviours within populations between years might be influenced by one or a combination of the following: (1) low genetic variation and/or phenotypic plasticity causing no flexibility in anti-predator behaviour, or (2) similar frequency of beetles in each ontogenetic stage, or due to (3) consistency of the environment so there is no difference in frequency of the expressed behaviours, lastly (4) unmeasured factors may affect the outcome so that different beetles can have different responses between years, but the overall frequency will remain, being interpreted as lack of variation. The variability and consistency of anti-predator behaviours are of equal importance for understanding how multiple anti-predator behaviours evolve and how they contribute to ecological interactions.

In conclusion, we found that ontogeny and environment (i.e. substrate under the host plant) affect the predator avoidance behaviour in our study populations of the green tortoise beetle *C. viridis.* Additionally, the anti-predator response varied within and between populations and years for ontogeny and tended to vary for substrate. This may indicate a dynamic relationship between predator avoidance behaviours, ontogenetic life stages, and mating status, and the local environment across time and space. Our study highlights the importance of using a multivariate approach to understand the ecological and evolutionary drivers of anti-predator responses. Further studies that include, e.g., variability in predation pressure or population genetics, could help us better understand the ecological interactions and how multiple anti-predator defences evolve and are being maintained in natural populations.

## Supplementary Information

Below is the link to the electronic supplementary material.

Supplementary Material 1

Supplementary Material 2

Supplementary Material 3

## Data Availability

Data is included as electronic supplementary material.

## Code availability

Code is included as electronic supplementary material.
