# Supplementary material for: Predator avoidance behaviour in green tortoise beetles depends on environment and shield ontogeny, but varies over time and space
Source: Oecologia. 2026 Nov 11;208(12):169. doi: 10.1007/s00442-026-06005-1 (PMC13751549; doi:10.1007/s00442-026-06005-1)
Supplement: Supplementary file 1 [file 442_2026_6005_MOESM1_ESM.pdf]

Drop.or.Duck	Substrate	Ontogeny	Population	Year	Hostplant
Duck	Water	hard-mature	Häckeberga	2023	Mint
Duck	Land	hard-mature	Björkelunda	2023	Mint
Drop	Land	soft-immature	Björkelunda	2023	Mint
Drop	Water	soft-immature	Häckeberga	2023	Mint
Drop	Land	soft-immature	Björkelunda	2023	Mint
Drop	Land	soft-immature	Nöbbelöv	2023	Mint
Duck	Water	soft-immature	Björkelunda	2023	Marsh woundwort
Duck	Land	hard-mature	Häckeberga	2023	Mint
Walk	Water	hard-mature	Häckeberga	2023	Mint
Drop	Water	soft-immature	Häckeberga	2023	Mint
Walk	Water	soft-immature	Björkelunda	2023	Mint
Drop	Land	hard-mature	Häckeberga	2023	Mint
Duck	Land	soft-immature	Häckeberga	2023	Mint
Duck	Water	soft-immature	Björkelunda	2023	Mint
Duck	Water	hard-mature	Björkelunda	2023	Mint
Drop	Water	hard-mature	Häckeberga	2023	Mint
Walk	Water	hard-mature	Häckeberga	2023	Gypsywort
Duck	Water	soft-immature	Björkelunda	2023	Mint
Walk	Water	soft-immature	Björkelunda	2023	Mint
Duck	Water	hard-mature	Björkelunda	2023	Mint
Drop	Water	hard-mature	Björkelunda	2023	Mint
Duck	Water	hard-mature	Häckeberga	2023	Mint
Drop	Water	soft-immature	Häckeberga	2023	Marsh woundwort
Duck	Water	hard-mature	Nöbbelöv	2023	Mint
Drop	Land	hard-mature	Häckeberga	2023	Mint
Duck	Water	hard-mature	Björkelunda	2023	Mint
Duck	Water	hard-mature	Häckeberga	2023	Mint
Duck	Water	soft-immature	Björkelunda	2023	Mint
Duck	Water	soft-immature	Björkelunda	2023	Mint
Duck	Water	soft-immature	Björkelunda	2023	Mint
Drop	Land	soft-immature	Björkelunda	2023	Mint
Duck	Water	soft-immature	Björkelunda	2023	Mint
Drop	Water	hard-mature	Björkelunda	2023	Mint
Walk	Land	soft-immature	Nöbbelöv	2023	Gypsywort
Duck	Land	hard-mature	Nöbbelöv	2023	Mint
Duck	Water	soft-immature	Björkelunda	2023	Marsh woundwort
Drop	Water	soft-immature	Häckeberga	2023	Marsh woundwort
Duck	Water	soft-immature	Björkelunda	2023	Mint
Drop	Land	hard-immature	Björkelunda	2023	Mint
Drop	Water	soft-immature	Häckeberga	2023	Mint
Duck	Water	soft-immature	Björkelunda	2023	Mint
Duck	Land	soft-immature	Häckeberga	2023	Mint
Drop	Water	soft-immature	Björkelunda	2023	Mint
Walk	Water	hard-mature	Björkelunda	2023	Mint
Drop	Land	hard-immature	Nöbbelöv	2023	Mint
Duck	Water	soft-immature	Björkelunda	2023	Mint
Drop	Water	soft-immature	Björkelunda	2023	Mint

Drop	Water	soft-immature	Björkelunda	2023	Mint
Duck	Land	soft-immature	Häckeberga	2023	Mint
Drop	Water	soft-immature	Björkelunda	2023	Mint
Duck	Water	soft-immature	Björkelunda	2023	Mint
Duck	Water	soft-immature	Björkelunda	2023	Mint
Duck	Water	hard-immature	Häckeberga	2023	Mint
Drop	Land	hard-mature	Björkelunda	2023	Mint
Drop	Land	hard-mature	Häckeberga	2023	Marsh woundwort
Duck	Land	soft-immature	Häckeberga	2023	Mint
Drop	Land	soft-immature	Björkelunda	2023	Mint
Walk	Water	hard-immature	Björkelunda	2023	Mint
Drop	Water	soft-immature	Nöbbelöv	2023	Mint
Duck	Water	hard-mature	Häckeberga	2023	Mint
Duck	Land	hard-mature	Häckeberga	2023	Mint
Duck	Land	hard-mature	Björkelunda	2023	Mint
Duck	Water	hard-immature	Björkelunda	2023	Mint
Duck	Water	hard-mature	Häckeberga	2023	Marsh woundwort
Walk	Land	soft-immature	Björkelunda	2023	Mint
Duck	Water	soft-immature	Häckeberga	2023	Marsh woundwort
Duck	Water	hard-immature	Björkelunda	2023	Mint
Duck	Land	hard-mature	Häckeberga	2023	Mint
Duck	Water	soft-immature	Björkelunda	2023	Mint
Drop	Land	soft-immature	Nöbbelöv	2023	Mint
Duck	Land	hard-mature	Björkelunda	2023	NA
Drop	Water	hard-mature	Björkelunda	2023	Mint
Duck	Land	hard-mature	Häckeberga	2023	Mint
Drop	Land	hard-mature	Nöbbelöv	2023	Mint
Drop	Water	hard-mature	Häckeberga	2023	Mint
Drop	Water	soft-immature	Björkelunda	2023	Mint
Drop	Land	soft-immature	Häckeberga	2023	Mint
Duck	Water	hard-mature	Häckeberga	2023	Mint
Drop	Water	soft-immature	Björkelunda	2023	Mint
Drop	Water	hard-mature	Björkelunda	2023	Mint
Duck	Water	hard-mature	Nöbbelöv	2023	Mint
Drop	Water	soft-immature	Häckeberga	2023	Marsh woundwort
Drop	Water	hard-immature	Björkelunda	2023	Mint
Duck	Water	hard-immature	Björkelunda	2023	Mint
Drop	Land	soft-immature	Nöbbelöv	2023	Gypsywort
Walk	Water	soft-immature	Björkelunda	2023	NA
Duck	Land	hard-mature	Björkelunda	2023	Mint
Drop	Land	soft-immature	Häckeberga	2023	Mint
Duck	Water	hard-mature	Björkelunda	2023	Mint
Drop	Water	hard-immature	Björkelunda	2023	Mint
Duck	Water	soft-immature	Björkelunda	2023	Mint
Drop	Water	soft-immature	Björkelunda	2023	Mint
Drop	Land	soft-immature	Häckeberga	2023	Mint
Drop	Water	soft-immature	Björkelunda	2023	Mint
Drop	Water	hard-mature	Häckeberga	2023	Mint

Walk	Water	hard-mature	Björkelunda	2023	Mint
Duck	Water	hard-mature	Häckeberga	2023	Mint
Walk	Land	soft-immature	Björkelunda	2023	Mint
Duck	Water	soft-immature	Björkelunda	2023	Mint
Drop	Land	hard-mature	Nöbbelöv	2023	NA
Duck	Water	soft-immature	Björkelunda	2023	Mint
Walk	Water	soft-immature	Björkelunda	2023	Mint
Duck	Water	hard-mature	Björkelunda	2023	Mint
Drop	Land	hard-immature	Nöbbelöv	2023	Mint
Drop	Water	soft-immature	Häckeberga	2023	Mint
Duck	Water	soft-immature	Häckeberga	2023	Mint
Drop	Water	soft-immature	Häckeberga	2023	Mint
Drop	Water	soft-immature	Björkelunda	2023	Mint
Duck	Water	hard-immature	Björkelunda	2023	Mint
Duck	Water	soft-immature	Häckeberga	2023	Gypsywort
Drop	Land	hard-mature	Björkelunda	2023	NA
Drop	Water	hard-mature	Nöbbelöv	2023	Mint
Drop	Land	soft-immature	Häckeberga	2023	Mint
Drop	Water	soft-immature	Björkelunda	2023	Mint
Duck	Water	hard-mature	Häckeberga	2023	Mint
Drop	Water	soft-immature	Björkelunda	2023	Mint
Duck	Water	soft-immature	Björkelunda	2023	Mint
Drop	Land	soft-immature	Nöbbelöv	2023	Gypsywort
Duck	Water	hard-mature	Björkelunda	2023	Mint
Duck	Water	soft-immature	Björkelunda	2023	Mint
Walk	Water	soft-immature	Björkelunda	2023	Mint
Duck	Water	soft-immature	Björkelunda	2023	Mint
Walk	Land	soft-immature	Björkelunda	2023	Marsh woundwort
Drop	Land	hard-immature	Nöbbelöv	2023	Mint
Duck	Water	hard-immature	Häckeberga	2023	Gypsywort
Drop	Land	soft-immature	Nöbbelöv	2023	Gypsywort
Walk	Water	soft-immature	Björkelunda	2023	Mint
Walk	Water	hard-mature	Häckeberga	2023	Mint
Duck	Water	soft-immature	Björkelunda	2023	Mint
Drop	Water	soft-immature	Nöbbelöv	2023	Mint
Drop	Land	hard-mature	Häckeberga	2023	Mint
Drop	Water	soft-immature	Björkelunda	2023	Mint
Drop	Water	soft-immature	Häckeberga	2023	Mint
Duck	Water	soft-immature	Björkelunda	2023	Mint
Duck	Water	soft-immature	Björkelunda	2023	Mint
Duck	Water	soft-immature	Björkelunda	2023	Mint
Drop	Land	hard-immature	Nöbbelöv	2023	NA
Drop	Land	soft-immature	Häckeberga	2023	Mint
Duck	Water	soft-immature	Björkelunda	2023	Mint
Drop	Water	soft-immature	Björkelunda	2023	Mint
Drop	Land	soft-immature	Häckeberga	2023	Mint
Drop	Water	hard-immature	Björkelunda	2023	Mint
Duck	Water	soft-immature	Björkelunda	2023	Mint

Duck	Water	hard-immature	Björkelunda	2023	Mint
Duck	Water	hard-mature	Nöbbelöv	2023	Mint
Drop	Land	hard-mature	Nöbbelöv	2023	Gypsywort
Duck	Water	hard-mature	Häckeberga	2023	Mint
Duck	Water	hard-mature	Häckeberga	2023	Marsh woundwort
Drop	Land	soft-immature	Häckeberga	2023	Mint
Drop	Water	soft-immature	Björkelunda	2023	Marsh woundwort
Drop	Land	soft-immature	Nöbbelöv	2023	Mint
Duck	Water	hard-mature	Björkelunda	2023	Mint
Drop	Water	soft-immature	Björkelunda	2023	Mint
Drop	Water	hard-immature	Björkelunda	2023	Mint
Drop	Water	hard-mature	Björkelunda	2023	Mint
Walk	Water	hard-mature	Björkelunda	2023	Mint
Drop	Land	soft-immature	Häckeberga	2023	Mint
Duck	Land	hard-mature	Häckeberga	2023	Mint
Duck	Water	hard-mature	Björkelunda	2023	Marsh woundwort
Drop	Water	hard-immature	Björkelunda	2023	Marsh woundwort
Duck	Water	soft-immature	Björkelunda	2023	Mint
Duck	Water	hard-mature	Björkelunda	2023	Mint
Duck	Water	soft-immature	Nöbbelöv	2023	NA
Duck	Water	hard-mature	Björkelunda	2023	Mint
Duck	Water	hard-mature	Björkelunda	2023	Mint
Walk	Water	hard-mature	Häckeberga	2023	Mint
Drop	Water	soft-immature	Björkelunda	2023	Mint
Duck	Water	hard-immature	Björkelunda	2023	Marsh woundwort
Drop	Water	soft-immature	Björkelunda	2023	Mint
Drop	Land	soft-immature	Häckeberga	2023	Mint
Duck	Water	hard-mature	Häckeberga	2023	Mint
Duck	Water	soft-immature	Björkelunda	2023	Mint
Drop	Water	soft-immature	Häckeberga	2023	Mint
Drop	Water	hard-immature	Björkelunda	2023	Mint
Duck	Water	soft-immature	Björkelunda	2023	Mint
Duck	Land	soft-immature	Häckeberga	2023	Mint
Drop	Water	soft-immature	Nöbbelöv	2023	Mint
Duck	Water	soft-immature	Björkelunda	2023	Mint
Drop	Land	soft-immature	Nöbbelöv	2023	Gypsywort
Drop	Land	hard-mature	Björkelunda	2023	NA
Drop	Water	hard-mature	Björkelunda	2023	Mint
Duck	Water	hard-immature	Björkelunda	2023	Mint
Drop	Water	hard-immature	Björkelunda	2023	Mint
Duck	Water	soft-immature	Björkelunda	2023	Marsh woundwort
Duck	Water	hard-mature	Nöbbelöv	2023	Mint
Walk	Water	hard-immature	Björkelunda	2023	Marsh woundwort
Duck	Land	hard-mature	Häckeberga	2023	Mint
Duck	Water	soft-immature	Häckeberga	2023	Mint
Walk	Water	soft-immature	Björkelunda	2023	Mint
Duck	Water	soft-immature	Björkelunda	2023	Mint
Duck	Water	hard-mature	Björkelunda	2023	Mint

Drop	Water	hard-immature	Björkelunda	2023	Mint
Duck	Water	soft-immature	Björkelunda	2023	Marsh woundwort
Drop	Water	soft-immature	Häckeberga	2023	Gypsywort
Drop	Water	soft-immature	Björkelunda	2023	Mint
Duck	Land	hard-mature	Häckeberga	2023	Mint
Drop	Water	soft-immature	Häckeberga	2023	Mint
Duck	Water	soft-immature	Björkelunda	2023	Mint
Drop	Water	soft-immature	Björkelunda	2023	Mint
Drop	Land	soft-immature	Nöbbelöv	2023	Gypsywort
Duck	Water	soft-immature	Björkelunda	2023	Mint
Duck	Water	soft-immature	Björkelunda	2023	Mint
Drop	Water	hard-immature	Häckeberga	2023	Mint
Duck	Water	hard-mature	Häckeberga	2023	NA
Duck	Water	hard-mature	Björkelunda	2023	Marsh woundwort
Drop	Water	soft-immature	Björkelunda	2023	Mint
Drop	Water	soft-immature	Nöbbelöv	2023	Mint
Drop	Land	soft-immature	Björkelunda	2023	Mint
Drop	Water	soft-immature	Björkelunda	2023	NA
Drop	Land	soft-immature	Häckeberga	2023	Mint
Duck	Water	soft-immature	Björkelunda	2023	Mint
Walk	Water	soft-immature	Björkelunda	2023	Mint
Drop	Water	hard-immature	Häckeberga	2023	Mint
Drop	Water	soft-immature	Björkelunda	2023	Mint
Duck	Water	soft-immature	Häckeberga	2023	Mint
Drop	Land	hard-mature	Häckeberga	2023	Mint
Duck	Land	hard-immature	Nöbbelöv	2023	Mint
Duck	Water	soft-immature	Björkelunda	2023	Mint
Drop	Water	soft-immature	Björkelunda	2023	Mint
Drop	Land	soft-immature	Nöbbelöv	2023	Mint
Drop	Water	soft-immature	Björkelunda	2023	Mint
Walk	Land	soft-immature	Björkelunda	2023	Mint
Drop	Water	soft-immature	Häckeberga	2023	Marsh woundwort
Walk	Land	soft-immature	Häckeberga	2023	Mint
Duck	Water	hard-mature	Häckeberga	2023	Marsh woundwort
Drop	Water	soft-immature	Björkelunda	2023	Mint
Drop	Water	hard-immature	Häckeberga	2023	Marsh woundwort
Duck	Water	soft-immature	Björkelunda	2023	Marsh woundwort
Duck	Water	hard-mature	Häckeberga	2023	Mint
Drop	Water	soft-immature	Björkelunda	2023	Mint
Duck	Water	soft-immature	Björkelunda	2023	Mint
Duck	Water	soft-immature	Björkelunda	2023	Mint
Drop	Land	soft-immature	Nöbbelöv	2023	Gypsywort
Duck	Water	soft-immature	Björkelunda	2023	Marsh woundwort
Drop	Water	hard-mature	Häckeberga	2023	Mint
Drop	Water	soft-immature	Björkelunda	2023	Marsh woundwort
Duck	Water	hard-mature	Björkelunda	2023	Mint
Duck	Land	soft-immature	Björkelunda	2023	Mint
Duck	Water	hard-mature	Björkelunda	2023	Mint

Drop	Water	hard-immature	Björkelunda	2023	Mint
Drop	Water	soft-immature	Björkelunda	2023	Mint
Duck	Water	hard-immature	Björkelunda	2023	Mint
Drop	Water	hard-immature	Björkelunda	2023	Mint
Drop	Land	hard-immature	Häckeberga	2023	Marsh woundwort
Duck	Water	hard-immature	Björkelunda	2023	Mint
Drop	Water	soft-immature	Björkelunda	2023	Mint
Duck	Water	soft-immature	Björkelunda	2023	Mint
Drop	Water	hard-mature	Björkelunda	2023	Mint
Drop	Water	hard-immature	Björkelunda	2023	Mint
Walk	Land	soft-immature	Björkelunda	2023	Mint
Walk	Water	hard-mature	Nöbbelöv	2023	Mint
Drop	Water	soft-immature	Björkelunda	2023	Mint
Duck	Water	soft-immature	Björkelunda	2023	Mint
Duck	Water	hard-mature	Björkelunda	2023	Mint
Duck	Water	soft-immature	Björkelunda	2023	Mint
Drop	Water	soft-immature	Björkelunda	2023	Mint
Drop	Water	hard-mature	Björkelunda	2023	NA
Duck	Water	soft-immature	Björkelunda	2023	Mint
Duck	Water	soft-immature	Björkelunda	2023	Mint
Duck	Water	soft-immature	Björkelunda	2023	Mint
Drop	Water	hard-immature	Häckeberga	2023	Mint
Duck	Water	hard-immature	Björkelunda	2023	Marsh woundwort
Duck	Water	soft-immature	Häckeberga	2023	Mint
Walk	Land	hard-mature	Nöbbelöv	2023	Gypsywort
Drop	Water	soft-immature	Björkelunda	2023	Mint
Duck	Water	hard-mature	Häckeberga	2023	Mint
Walk	Water	soft-immature	Björkelunda	2023	Mint
Walk	Water	soft-immature	Björkelunda	2023	Mint
Drop	Water	soft-immature	Häckeberga	2023	Mint
Walk	Water	soft-immature	Björkelunda	2023	Mint
Drop	Water	hard-immature	Björkelunda	2023	Mint
Drop	Water	soft-immature	Björkelunda	2023	Mint
Drop	Water	hard-mature	Häckeberga	2023	Mint
Duck	Water	hard-immature	Björkelunda	2023	Marsh woundwort
Duck	Water	hard-immature	Björkelunda	2023	Mint
Duck	Water	hard-mature	Nöbbelöv	2023	NA
Duck	Water	soft-immature	Björkelunda	2023	Mint
Duck	Water	hard-mature	Häckeberga	2023	Mint
Drop	Water	soft-immature	Björkelunda	2023	Mint
Drop	Water	hard-mature	Häckeberga	2023	Mint
Walk	Water	hard-immature	Häckeberga	2023	NA
Drop	Water	soft-immature	Björkelunda	2023	Mint
Drop	Land	soft-immature	Nöbbelöv	2023	Mint
Drop	Water	hard-mature	Häckeberga	2023	Mint
Duck	Water	soft-immature	Häckeberga	2023	Mint
Walk	Water	soft-immature	Björkelunda	2023	Mint
Walk	Water	soft-immature	Björkelunda	2023	Mint

Drop	Water	hard-mature	Häckeberga	2023	Marsh woundwort
Drop	Water	hard-mature	Björkelunda	2023	Mint
Duck	Land	hard-mature	Häckeberga	2023	Mint
Duck	Water	hard-mature	Häckeberga	2023	Mint
Duck	Water	hard-mature	Björkelunda	2023	Mint
Duck	Water	soft-immature	Björkelunda	2023	Mint
Duck	Water	soft-immature	Häckeberga	2023	Mint
Drop	Land	hard-mature	Björkelunda	2023	Mint
Walk	Water	hard-immature	Björkelunda	2023	Mint
Duck	Water	soft-immature	Björkelunda	2023	Mint
Drop	Land	soft-immature	Nöbbelöv	2023	Mint
Duck	Water	hard-immature	Björkelunda	2023	Mint
Walk	Water	soft-immature	Björkelunda	2023	Mint
Drop	Water	hard-immature	Björkelunda	2023	Mint
Duck	Water	soft-immature	Björkelunda	2023	Mint
Duck	Land	soft-immature	Häckeberga	2023	Marsh woundwort
Walk	Water	soft-immature	Björkelunda	2023	Mint
Drop	Water	hard-immature	Björkelunda	2023	Marsh woundwort
Duck	Water	soft-immature	Björkelunda	2023	Marsh woundwort
Duck	Water	soft-immature	Björkelunda	2023	Mint
Duck	Water	hard-mature	Björkelunda	2023	Mint
Duck	Water	soft-immature	Björkelunda	2023	Mint
Drop	Land	soft-immature	Häckeberga	2023	Mint
Drop	Water	soft-immature	Häckeberga	2023	Mint
Drop	Water	soft-immature	Björkelunda	2023	Mint
Drop	Water	soft-immature	Björkelunda	2023	Mint
Duck	Water	hard-mature	Björkelunda	2023	Mint
Drop	Water	hard-immature	Björkelunda	2023	Mint
Duck	Water	hard-immature	Björkelunda	2023	NA
Walk	Water	hard-mature	Nöbbelöv	2023	NA
Duck	Land	hard-mature	Häckeberga	2023	Mint
Duck	Water	hard-mature	Björkelunda	2023	Mint
Drop	Water	hard-mature	Häckeberga	2023	Mint
Drop	Land	hard-immature	Björkelunda	2023	Gypsywort
Duck	Water	soft-immature	Björkelunda	2023	Mint
Drop	Water	hard-immature	Häckeberga	2023	Marsh woundwort
Drop	Water	hard-mature	Björkelunda	2023	Mint
Walk	Water	soft-immature	Björkelunda	2023	Mint
Duck	Land	hard-mature	Häckeberga	2023	Mint
Walk	Water	soft-immature	Björkelunda	2023	Mint
Drop	Land	hard-mature	Björkelunda	2023	Mint
Duck	Water	soft-immature	Björkelunda	2023	Mint
Drop	Water	soft-immature	Häckeberga	2023	Marsh woundwort
Duck	Water	soft-immature	Björkelunda	2023	Mint
Duck	Water	hard-immature	Björkelunda	2023	Mint
Drop	Land	hard-mature	Häckeberga	2023	Mint
Duck	Water	hard-mature	Häckeberga	2023	Mint
Duck	Water	soft-immature	Björkelunda	2023	Mint

Duck	Land	hard-mature	Nöbbelöv	2023	Mint
Drop	Land	hard-immature	Björkelunda	2023	Mint
Duck	Land	soft-immature	Björkelunda	2023	Mint
Walk	Water	soft-immature	Björkelunda	2023	Mint
Drop	Water	hard-immature	Björkelunda	2023	Mint
Drop	Land	soft-immature	Björkelunda	2023	Mint
Duck	Water	soft-immature	Häckeberga	2023	Marsh woundwort
Duck	Water	soft-immature	Björkelunda	2023	Mint
Drop	Land	hard-mature	Björkelunda	2023	Mint
Duck	Water	hard-mature	Björkelunda	2023	Mint
Duck	Water	hard-mature	Häckeberga	2023	Mint
Walk	Land	soft-immature	Björkelunda	2023	Mint
Walk	Land	soft-immature	Björkelunda	2023	Mint
Drop	Water	hard-mature	Häckeberga	2023	Gypsywort
Duck	Water	soft-immature	Björkelunda	2023	Mint
Duck	Land	soft-immature	Häckeberga	2023	Mint
Walk	Land	hard-immature	Björkelunda	2023	Mint
Drop	Water	soft-immature	Björkelunda	2023	Mint
Drop	Water	hard-immature	Häckeberga	2023	Marsh woundwort
Duck	Water	soft-immature	Björkelunda	2023	Mint
Duck	Land	hard-mature	Häckeberga	2023	Mint
Duck	Water	soft-immature	Björkelunda	2023	Mint
Drop	Water	soft-immature	Björkelunda	2023	Mint
Drop	Land	hard-mature	Nöbbelöv	2023	Gypsywort
Duck	Water	hard-mature	Björkelunda	2023	Mint
Duck	Land	soft-immature	Häckeberga	2023	Mint
Duck	Water	hard-mature	Björkelunda	2023	Mint
Drop	Water	soft-immature	Björkelunda	2023	Mint
Duck	Water	soft-immature	Björkelunda	2023	Mint
Drop	Land	soft-immature	Häckeberga	2023	Mint
Duck	Water	soft-immature	Björkelunda	2023	Mint
Duck	Water	hard-mature	Häckeberga	2023	Mint
Drop	Water	soft-immature	Björkelunda	2023	Mint
Duck	Water	hard-immature	Björkelunda	2023	Mint
Duck	Land	hard-immature	Björkelunda	2023	Mint
Duck	Water	soft-immature	Björkelunda	2023	Mint
Drop	Land	soft-immature	Björkelunda	2023	Mint
Drop	Water	soft-immature	Häckeberga	2023	Marsh woundwort
Duck	Water	soft-immature	Björkelunda	2023	Mint
Drop	Water	soft-immature	Björkelunda	2023	Mint
Duck	Water	hard-immature	Björkelunda	2023	Mint
Drop	Water	hard-immature	Björkelunda	2023	Mint
Drop	Water	hard-immature	Björkelunda	2023	Mint
Duck	Water	hard-mature	Björkelunda	2023	Mint
Duck	Land	soft-immature	Björkelunda	2023	Mint
Duck	Water	soft-immature	Björkelunda	2023	Mint
Duck	Water	hard-immature	Häckeberga	2023	Gypsywort
Drop	Water	hard-immature	Björkelunda	2023	Marsh woundwort

Duck	Land	hard-mature	Häckeberga	2023	Mint
Duck	Water	soft-immature	Björkelunda	2023	Mint
Walk	Water	soft-immature	Björkelunda	2023	Mint
Duck	Water	soft-immature	Björkelunda	2023	Mint
Drop	Water	hard-immature	Björkelunda	2023	Mint
Duck	Water	soft-immature	Björkelunda	2023	Mint
Duck	Water	hard-mature	Björkelunda	2023	Mint
Drop	Land	hard-mature	Häckeberga	2023	Mint
Walk	Land	soft-immature	Björkelunda	2023	Mint
Duck	Water	hard-mature	Björkelunda	2023	Mint
Drop	Water	soft-immature	Häckeberga	2023	Mint
Duck	Land	hard-mature	Häckeberga	2023	Mint
Drop	Water	hard-mature	Björkelunda	2023	Mint
Duck	Water	soft-immature	Häckeberga	2023	Mint
Duck	Water	hard-mature	Häckeberga	2023	Marsh woundwort
Duck	Water	soft-immature	Björkelunda	2023	Mint
Duck	Water	hard-immature	Björkelunda	2023	Mint
Duck	Water	soft-immature	Häckeberga	2023	Mint
Walk	Water	soft-immature	Björkelunda	2023	Mint
Drop	Water	soft-immature	Björkelunda	2023	Marsh woundwort
Duck	Water	hard-mature	Häckeberga	2023	Gypsywort
Duck	Water	hard-mature	Häckeberga	2023	Mint
Drop	Water	soft-immature	Björkelunda	2023	Mint
Drop	Water	soft-immature	Häckeberga	2023	Marsh woundwort
Drop	Land	soft-immature	Häckeberga	2023	Mint
Walk	Water	soft-immature	Björkelunda	2023	Mint
Drop	Water	soft-immature	Björkelunda	2023	Mint
Duck	Water	hard-mature	Häckeberga	2023	Mint
Duck	Water	soft-immature	Björkelunda	2023	Mint
Duck	Water	soft-immature	Björkelunda	2023	Mint
Duck	Water	hard-mature	Björkelunda	2023	Mint
Duck	Land	hard-mature	Nöbbelöv	2023	Mint
Drop	Water	hard-immature	Björkelunda	2023	Marsh woundwort
Duck	Land	hard-mature	Björkelunda	2023	Mint
Drop	Water	soft-immature	Björkelunda	2023	Mint
Drop	Land	hard-mature	Häckeberga	2023	Mint
Drop	Water	soft-immature	Björkelunda	2023	Mint
Walk	Water	hard-mature	Häckeberga	2023	Marsh woundwort
Drop	Water	soft-immature	Björkelunda	2023	Mint
Drop	Water	soft-immature	Häckeberga	2023	Mint
Walk	Water	hard-mature	Häckeberga	2023	Mint
Duck	Water	soft-immature	Häckeberga	2023	NA
Drop	Land	hard-mature	Nöbbelöv	2023	Gypsywort
Drop	Water	soft-immature	Björkelunda	2023	Mint
Drop	Water	soft-immature	Björkelunda	2023	Mint
Drop	Water	soft-immature	Björkelunda	2023	Mint
Drop	Water	soft-immature	Häckeberga	2023	Marsh woundwort
Duck	Water	soft-immature	Björkelunda	2023	Mint

Duck	Water	hard-immature	Nöbbelöv	2023	Mint
Duck	Water	soft-immature	Björkelunda	2023	Mint
Walk	Water	hard-immature	Björkelunda	2023	Mint
Duck	Water	hard-mature	Häckeberga	2023	Mint
Duck	Water	hard-immature	Björkelunda	2023	Mint
Walk	Water	hard-mature	Häckeberga	2023	Mint
Drop	Water	hard-mature	Björkelunda	2023	NA
Drop	Water	soft-immature	Björkelunda	2023	Mint
Drop	Water	soft-immature	Björkelunda	2023	Mint
Drop	Water	soft-immature	Häckeberga	2023	Mint
Duck	Water	soft-immature	Björkelunda	2023	Mint
Drop	Water	soft-immature	Björkelunda	2023	Mint
Drop	Water	soft-immature	Björkelunda	2023	Mint
Duck	Water	soft-immature	Häckeberga	2023	Mint
Duck	Land	soft-immature	Björkelunda	2023	Mint
Duck	Land	soft-immature	Nöbbelöv	2023	Mint
Drop	Water	soft-immature	Häckeberga	2023	Marsh woundwort
Drop	Land	hard-immature	Nöbbelöv	2023	Mint
Walk	Water	soft-immature	Björkelunda	2023	Mint
Drop	Water	soft-immature	Häckeberga	2023	Mint
Drop	Water	soft-immature	Björkelunda	2023	Marsh woundwort
Duck	Land	soft-immature	Häckeberga	2023	Mint
Duck	Water	hard-mature	Häckeberga	2023	Mint
Duck	Water	soft-immature	Nöbbelöv	2023	Mint
Drop	Land	soft-immature	Björkelunda	2023	Mint
Walk	Water	hard-mature	Häckeberga	2023	Mint
Drop	Land	soft-immature	Björkelunda	2023	Mint
Duck	Water	soft-immature	Björkelunda	2023	Mint
Drop	Land	hard-immature	Nöbbelöv	2023	Gypsywort
Duck	Water	hard-mature	Häckeberga	2023	Mint
Duck	Water	soft-immature	Björkelunda	2023	Mint
Duck	Water	soft-immature	Björkelunda	2023	Mint
Drop	Land	soft-immature	Björkelunda	2023	Marsh woundwort
Walk	Water	hard-mature	Häckeberga	2023	Mint
Drop	Land	soft-immature	Häckeberga	2023	Mint
Drop	Water	hard-mature	Björkelunda	2023	Mint
Duck	Water	hard-mature	Häckeberga	2023	Marsh woundwort
Drop	Water	soft-immature	Björkelunda	2023	Mint
Drop	Water	hard-mature	Häckeberga	2023	Mint
Duck	Water	soft-immature	Björkelunda	2023	Marsh woundwort
Drop	Land	hard-mature	Häckeberga	2023	Mint
Duck	Water	hard-immature	Björkelunda	2023	Mint
Drop	Water	soft-immature	Häckeberga	2023	Marsh woundwort
Duck	Water	hard-mature	Björkelunda	2023	Mint
Drop	Water	soft-immature	Björkelunda	2023	Mint
Duck	Water	soft-immature	Björkelunda	2023	Mint
Duck	Water	hard-immature	Björkelunda	2023	Mint
Duck	Land	hard-mature	Björkelunda	2023	Mint

Duck	Water	hard-immature	Björkelunda	2023	Mint
Drop	Water	hard-mature	Häckeberga	2023	Mint
Duck	Land	soft-immature	Nöbbelöv	2023	Gypsywort
Duck	Water	soft-immature	Björkelunda	2023	Mint
Duck	Water	soft-immature	Björkelunda	2023	Mint
Walk	Water	hard-mature	Häckeberga	2023	Mint
Duck	Water	soft-immature	Björkelunda	2023	Mint
Drop	Water	soft-immature	Häckeberga	2023	Marsh woundwort
Drop	Water	soft-immature	Björkelunda	2023	Mint
Duck	Land	soft-immature	Häckeberga	2023	Marsh woundwort
Drop	Land	hard-immature	Häckeberga	2023	Mint
Drop	Water	hard-mature	Björkelunda	2023	Mint
Duck	Water	soft-immature	Björkelunda	2023	Mint
Walk	Water	soft-immature	Björkelunda	2023	Mint
Drop	Land	soft-immature	Nöbbelöv	2023	Mint
Drop	Water	hard-immature	Häckeberga	2023	Mint
Walk	Land	hard-mature	Nöbbelöv	2023	Gypsywort
Drop	Water	soft-immature	Häckeberga	2023	Mint
Drop	Land	hard-immature	Björkelunda	2023	Mint
Duck	Water	soft-immature	Björkelunda	2023	Mint
Drop	Water	soft-immature	Björkelunda	2023	Mint
Drop	Water	hard-immature	Björkelunda	2023	Mint
Duck	Land	hard-mature	Björkelunda	2023	Mint
Duck	Water	soft-immature	Björkelunda	2023	Mint
Duck	Water	soft-immature	Björkelunda	2023	Mint
Duck	Water	soft-immature	Björkelunda	2023	Mint
Walk	Water	soft-immature	Björkelunda	2023	Mint
Duck	Water	hard-mature	Nöbbelöv	2023	Mint
Duck	Land	hard-mature	Häckeberga	2023	Mint
Duck	Water	hard-mature	Häckeberga	2023	Mint
Duck	Water	hard-immature	Björkelunda	2023	NA
Duck	Water	hard-immature	Björkelunda	2023	Marsh woundwort
Duck	Land	hard-mature	Häckeberga	2023	Mint
Drop	Water	soft-immature	Björkelunda	2023	Mint
Duck	Water	hard-mature	Häckeberga	2023	Mint
Drop	Water	soft-immature	Häckeberga	2023	Mint
Walk	Water	soft-immature	Björkelunda	2023	Mint
Duck	Water	hard-immature	Björkelunda	2023	NA
Walk	Water	soft-immature	Björkelunda	2023	Mint
Duck	Water	hard-mature	Nöbbelöv	2023	Mint
Duck	Water	hard-immature	Björkelunda	2023	Mint
Drop	Land	soft-immature	Nöbbelöv	2023	Gypsywort
Drop	Water	soft-immature	Björkelunda	2023	Mint
Duck	Water	hard-immature	Björkelunda	2023	Mint
Duck	Water	soft-immature	Häckeberga	2023	Mint
Duck	Water	hard-mature	Häckeberga	2023	Mint
Duck	Water	soft-immature	Björkelunda	2023	Mint
Duck	Land	soft-immature	Björkelunda	2023	Mint

Duck	Water	soft-immature	Björkelunda	2023	Mint
Duck	Water	soft-immature	Häckeberga	2023	Mint
Duck	Land	hard-mature	Nöbbelöv	2023	Mint
Walk	Water	soft-immature	Björkelunda	2023	Mint
Drop	Water	soft-immature	Häckeberga	2023	Mint
Drop	Land	soft-immature	Häckeberga	2023	Mint
Drop	Land	hard-mature	Häckeberga	2023	Marsh woundwort
Duck	Land	hard-mature	Häckeberga	2023	Mint
Duck	Water	hard-mature	Björkelunda	2023	Mint
Duck	Water	hard-mature	Björkelunda	2023	Mint
Drop	Water	soft-immature	Nöbbelöv	2023	Mint
Duck	Water	hard-mature	Häckeberga	2023	Mint
Duck	Water	soft-immature	Björkelunda	2023	Mint
Drop	Water	hard-immature	Björkelunda	2023	Mint
Duck	Land	hard-immature	Nöbbelöv	2023	Mint
Duck	Water	soft-immature	Björkelunda	2023	Mint
Duck	Water	soft-immature	Björkelunda	2023	Mint
Duck	Water	soft-immature	Björkelunda	2023	Mint
Duck	Water	hard-mature	Björkelunda	2023	Mint
Drop	Water	soft-immature	Björkelunda	2023	Mint
Duck	Water	hard-immature	Häckeberga	2023	Mint
Drop	Land	hard-mature	Björkelunda	2023	Mint
Drop	Land	soft-immature	Nöbbelöv	2023	Mint
Walk	Water	hard-mature	Häckeberga	2023	Mint
Duck	Land	hard-immature	Björkelunda	2023	Mint
Duck	Water	soft-immature	Häckeberga	2023	Gypsywort
Duck	Water	soft-immature	Björkelunda	2023	Mint
Duck	Water	hard-mature	Häckeberga	2023	Marsh woundwort
Duck	Water	soft-immature	Björkelunda	2023	Mint
Drop	Land	hard-mature	Häckeberga	2023	Mint
Duck	Water	hard-mature	Häckeberga	2023	NA
Duck	Water	soft-immature	Björkelunda	2023	Marsh woundwort
Drop	Land	soft-immature	Nöbbelöv	2023	Mint
Duck	Water	hard-immature	Björkelunda	2023	Mint
Drop	Water	soft-immature	Björkelunda	2023	Mint
Drop	Land	soft-immature	Häckeberga	2023	Mint
Duck	Water	hard-immature	Björkelunda	2023	Mint
Drop	Water	soft-immature	Björkelunda	2023	Mint
Drop	Land	soft-immature	Björkelunda	2023	Mint
Drop	Water	hard-mature	Björkelunda	2023	Mint
Duck	Land	hard-mature	Häckeberga	2023	Mint
Drop	Water	hard-mature	Häckeberga	2023	Marsh woundwort
Drop	Water	hard-mature	Björkelunda	2023	Mint
Duck	Land	hard-mature	Häckeberga	2023	Gypsywort
Drop	Land	hard-mature	Nöbbelöv	2023	Gypsywort
Drop	Water	soft-immature	Björkelunda	2023	Mint
Duck	Land	hard-immature	Björkelunda	2022	Mint
Drop	Water	hard-mature	Björkelunda	2022	Mint

Drop	Land	soft-immature	Nöbbelöv	2022	Gypsywort
Drop	Water	soft-immature	Häckeberga	2022	Mint
Drop	Land	hard-mature	Björkelunda	2022	Mint
Duck	Land	soft-immature	Nöbbelöv	2022	Gypsywort
Duck	Water	hard-immature	Häckeberga	2022	Marsh woundwort
Drop	Water	hard-immature	Häckeberga	2022	Mint
Drop	Land	hard-mature	Häckeberga	2022	Mint
Drop	Water	hard-mature	Björkelunda	2022	Mint
Duck	Land	soft-immature	Nöbbelöv	2022	Mint
Duck	Water	soft-immature	Häckeberga	2022	Mint
Drop	Water	soft-immature	Häckeberga	2022	Mint
Drop	Water	hard-mature	Häckeberga	2022	Mint
Drop	Water	hard-immature	Häckeberga	2022	Mint
Duck	Water	hard-mature	Häckeberga	2022	Marsh woundwort
Drop	Land	hard-mature	Björkelunda	2022	Mint
Duck	Water	hard-mature	Björkelunda	2022	Mint
Duck	Land	hard-mature	Björkelunda	2022	NA
Drop	Land	soft-immature	Häckeberga	2022	Mint
Duck	Water	hard-mature	Björkelunda	2022	Mint
Duck	Water	hard-mature	Häckeberga	2022	Mint
Drop	Land	hard-immature	Nöbbelöv	2022	Gypsywort
Duck	Water	hard-mature	Häckeberga	2022	Marsh woundwort
Duck	Water	hard-mature	Häckeberga	2022	Mint
Drop	Water	hard-immature	Björkelunda	2022	Mint
Duck	Land	hard-mature	Nöbbelöv	2022	Gypsywort
Duck	Land	hard-immature	Häckeberga	2022	Gypsywort
Drop	Land	hard-immature	Björkelunda	2022	Mint
Duck	Water	soft-immature	Häckeberga	2022	Marsh woundwort
Drop	Land	hard-immature	Nöbbelöv	2022	Gypsywort
Walk	Water	soft-immature	Häckeberga	2022	Mint
Drop	Land	soft-immature	Häckeberga	2022	Mint
Duck	Water	hard-mature	Björkelunda	2022	Mint
Drop	Water	soft-immature	Björkelunda	2022	Mint
Duck	Water	hard-mature	Björkelunda	2022	Mint
Walk	Water	soft-immature	Häckeberga	2022	Mint
Duck	Water	soft-immature	Häckeberga	2022	Mint
Drop	Land	hard-mature	Björkelunda	2022	Mint
Duck	Water	hard-mature	Björkelunda	2022	Mint
Drop	Water	hard-immature	Björkelunda	2022	Mint
Drop	Land	hard-immature	Björkelunda	2022	Mint
Duck	Water	hard-mature	Björkelunda	2022	Mint
Duck	Water	soft-immature	Häckeberga	2022	Marsh woundwort
Duck	Water	hard-mature	Nöbbelöv	2022	Mint
Duck	Water	hard-immature	Häckeberga	2022	Mint
Duck	Land	hard-immature	Häckeberga	2022	Mint
Duck	Water	hard-mature	Nöbbelöv	2022	Mint
Duck	Water	hard-mature	Häckeberga	2022	Marsh woundwort
Duck	Land	hard-immature	Björkelunda	2022	Mint

Duck	Land	soft-immature	Häckeberga	2022	NA
Duck	Land	hard-mature	Häckeberga	2022	Mint
Drop	Water	soft-immature	Björkelunda	2022	Mint
Duck	Land	hard-mature	Häckeberga	2022	Mint
Drop	Water	soft-immature	Björkelunda	2022	Mint
Drop	Land	soft-immature	Björkelunda	2022	Mint
Drop	Land	hard-immature	Nöbbelöv	2022	Gypsywort
Duck	Land	hard-immature	Nöbbelöv	2022	NA
Duck	Water	hard-mature	Björkelunda	2022	Mint
Duck	Land	hard-immature	Björkelunda	2022	Mint
Drop	Water	soft-immature	Björkelunda	2022	Mint
Drop	Water	hard-immature	Björkelunda	2022	Mint
Duck	Land	soft-immature	Nöbbelöv	2022	Gypsywort
Duck	Water	soft-immature	Häckeberga	2022	Mint
Duck	Water	soft-immature	Häckeberga	2022	Mint
Duck	Water	hard-mature	Häckeberga	2022	Marsh woundwort
Drop	Water	soft-immature	Häckeberga	2022	Mint
Duck	Water	soft-immature	Häckeberga	2022	Marsh woundwort
Duck	Water	hard-mature	Häckeberga	2022	Mint
Duck	Water	hard-mature	Häckeberga	2022	Mint
Drop	Land	hard-mature	Häckeberga	2022	Mint
Walk	Water	soft-immature	Häckeberga	2022	Mint
Duck	Land	soft-immature	Björkelunda	2022	Mint
Drop	Water	hard-mature	Björkelunda	2022	Marsh woundwort
Duck	Water	soft-immature	Björkelunda	2022	Mint
Duck	Water	hard-mature	Häckeberga	2022	Mint
Drop	Land	hard-immature	Nöbbelöv	2022	Mint
Duck	Water	hard-mature	Björkelunda	2022	Mint
Duck	Land	hard-mature	Björkelunda	2022	Mint
Drop	Water	soft-immature	Häckeberga	2022	Mint
Duck	Land	soft-immature	Nöbbelöv	2022	Mint
Duck	Water	hard-mature	Björkelunda	2022	Mint
Drop	Water	hard-mature	Häckeberga	2022	Mint
Duck	Water	hard-immature	Björkelunda	2022	Mint
Walk	Water	hard-mature	Björkelunda	2022	Mint
Walk	Water	hard-mature	Björkelunda	2022	Mint
Drop	Water	soft-immature	Häckeberga	2022	Mint
Duck	Water	hard-mature	Häckeberga	2022	Mint
Drop	Land	hard-immature	Nöbbelöv	2022	Mint
Drop	Water	hard-mature	Björkelunda	2022	Mint
Duck	Water	hard-immature	Häckeberga	2022	Mint
Duck	Land	soft-immature	Björkelunda	2022	Mint
Drop	Water	soft-immature	Häckeberga	2022	Mint
Drop	Water	hard-immature	Häckeberga	2022	Mint
Drop	Land	soft-immature	Nöbbelöv	2022	Gypsywort
Duck	Water	hard-mature	Häckeberga	2022	Mint
Duck	Water	soft-immature	Björkelunda	2022	Mint
Drop	Land	soft-immature	Björkelunda	2022	Mint

Duck	Land	hard-immature	Nöbbelöv	2022	Gypsywort
Drop	Land	hard-immature	Nöbbelöv	2022	Mint
Drop	Water	hard-immature	Björkelunda	2022	Mint
Drop	Land	hard-immature	Björkelunda	2022	Mint
Duck	Land	soft-immature	Nöbbelöv	2022	Gypsywort
Drop	Water	soft-immature	Häckeberga	2022	Mint
Duck	Water	hard-mature	Björkelunda	2022	Mint
Drop	Water	soft-immature	Häckeberga	2022	Mint
Drop	Land	hard-immature	Björkelunda	2022	Mint
Drop	Water	soft-immature	Björkelunda	2022	Mint
Duck	Land	hard-mature	Björkelunda	2022	Mint
Duck	Land	soft-immature	Nöbbelöv	2022	Gypsywort
Drop	Land	soft-immature	Häckeberga	2022	Marsh woundwort
Drop	Land	soft-immature	Häckeberga	2022	Mint
Drop	Land	soft-immature	Nöbbelöv	2022	Gypsywort
Duck	Land	hard-immature	Nöbbelöv	2022	Mint
Duck	Water	hard-mature	Häckeberga	2022	Mint
Drop	Water	soft-immature	Björkelunda	2022	Mint
Walk	Land	hard-mature	Björkelunda	2022	Mint
Drop	Water	hard-mature	Häckeberga	2022	Marsh woundwort
Duck	Land	soft-immature	Nöbbelöv	2022	Mint
Walk	Water	hard-mature	Björkelunda	2022	Mint
Drop	Water	hard-immature	Häckeberga	2022	Marsh woundwort
Drop	Water	hard-immature	Häckeberga	2022	Mint
Duck	Water	hard-mature	Häckeberga	2022	Mint
Duck	Land	hard-immature	Nöbbelöv	2022	Mint
Duck	Water	hard-mature	Häckeberga	2022	Mint
Drop	Water	hard-mature	Häckeberga	2022	Gypsywort
Drop	Water	soft-immature	Häckeberga	2022	Mint
Duck	Water	hard-mature	Björkelunda	2022	Mint
Drop	Land	hard-mature	Björkelunda	2022	Mint
Duck	Land	hard-immature	Nöbbelöv	2022	Gypsywort
Walk	Water	hard-mature	Björkelunda	2022	Mint
Drop	Land	hard-immature	Björkelunda	2022	Mint
Duck	Water	hard-mature	Häckeberga	2022	Marsh woundwort
Duck	Water	soft-immature	Björkelunda	2022	Mint
Duck	Water	soft-immature	Björkelunda	2022	Mint
Duck	Water	hard-mature	Björkelunda	2022	Mint
Drop	Land	soft-immature	Nöbbelöv	2022	Gypsywort
Drop	Land	soft-immature	Björkelunda	2022	Mint
Duck	Water	hard-immature	Björkelunda	2022	Mint
Drop	Water	hard-immature	Björkelunda	2022	Mint
Drop	Water	soft-immature	Häckeberga	2022	Mint
Drop	Land	soft-immature	Nöbbelöv	2022	Gypsywort
Duck	Land	hard-immature	Björkelunda	2022	Mint
Duck	Land	soft-immature	Nöbbelöv	2022	Gypsywort
Drop	Land	hard-immature	Nöbbelöv	2022	Mint
Duck	Land	hard-mature	Nöbbelöv	2022	Mint

Duck	Land	hard-immature	Björkelunda	2022	Mint
Walk	Water	hard-mature	Häckeberga	2022	Mint
Drop	Water	soft-immature	Häckeberga	2022	Mint
Walk	Land	hard-mature	Björkelunda	2022	Gypsywort
Duck	Water	hard-immature	Björkelunda	2022	Mint
Drop	Water	soft-immature	Häckeberga	2022	Mint
Duck	Water	hard-mature	Häckeberga	2022	Marsh woundwort
Drop	Water	soft-immature	Häckeberga	2022	Mint
Duck	Water	hard-mature	Nöbbelöv	2022	Mint
Duck	Water	soft-immature	Björkelunda	2022	Mint
Drop	Land	soft-immature	Häckeberga	2022	Mint
Drop	Water	hard-mature	Björkelunda	2022	Mint
Duck	Land	hard-mature	Björkelunda	2022	Marsh woundwort
Drop	Water	hard-immature	Björkelunda	2022	Mint
Drop	Land	soft-immature	Björkelunda	2022	Mint
Drop	Water	hard-mature	Björkelunda	2022	Mint
Drop	Land	hard-immature	Nöbbelöv	2022	Gypsywort
Duck	Land	hard-mature	Nöbbelöv	2022	Gypsywort
Duck	Water	hard-mature	Björkelunda	2022	Marsh woundwort
Duck	Water	hard-mature	Björkelunda	2022	Mint
Duck	Land	hard-mature	Nöbbelöv	2022	Gypsywort
Walk	Water	hard-mature	Björkelunda	2022	Mint
Drop	Water	soft-immature	Björkelunda	2022	Mint
Drop	Water	soft-immature	Häckeberga	2022	Mint
Duck	Water	hard-mature	Björkelunda	2022	Mint
Drop	Land	soft-immature	Björkelunda	2022	Mint
Duck	Water	soft-immature	Björkelunda	2022	Mint
Drop	Water	soft-immature	Häckeberga	2022	Mint
Drop	Land	hard-immature	Nöbbelöv	2022	Mint
Duck	Water	soft-immature	Häckeberga	2022	Marsh woundwort
Duck	Water	hard-immature	Björkelunda	2022	Mint
Walk	Water	soft-immature	Häckeberga	2022	Mint
Duck	Land	hard-immature	Nöbbelöv	2022	Gypsywort
Drop	Land	hard-immature	Nöbbelöv	2022	Gypsywort
Duck	Water	hard-immature	Häckeberga	2022	Mint
Duck	Water	hard-mature	Häckeberga	2022	Mint
Drop	Water	hard-mature	Björkelunda	2022	Mint
Drop	Water	hard-mature	Häckeberga	2022	Marsh woundwort
Drop	Water	hard-immature	Björkelunda	2022	Mint
Duck	Land	hard-mature	Nöbbelöv	2022	Mint
Duck	Water	hard-mature	Häckeberga	2022	Mint
Drop	Land	soft-immature	Nöbbelöv	2022	Mint
Duck	Land	soft-immature	Nöbbelöv	2022	Mint
Duck	Water	hard-mature	Häckeberga	2022	Mint
Duck	Water	hard-immature	Häckeberga	2022	Mint
Duck	Water	soft-immature	Häckeberga	2022	Marsh woundwort
Drop	Water	hard-immature	Björkelunda	2022	Mint
Duck	Water	soft-immature	Björkelunda	2022	Mint

Duck	Land	hard-mature	Björkelunda	2022	Mint
Duck	Water	soft-immature	Häckeberga	2022	Marsh woundwort
Duck	Water	hard-immature	Häckeberga	2022	Mint
Duck	Water	hard-mature	Björkelunda	2022	Gypsywort
Duck	Land	hard-mature	Björkelunda	2022	Mint
Drop	Water	hard-mature	Häckeberga	2022	Mint
Drop	Land	soft-immature	Nöbbelöv	2022	Gypsywort
Walk	Land	hard-mature	Nöbbelöv	2022	Mint
Duck	Water	soft-immature	Björkelunda	2022	Mint
Drop	Land	soft-immature	Björkelunda	2022	Mint
Duck	Water	hard-mature	Björkelunda	2022	Mint
Drop	Water	hard-immature	Häckeberga	2022	Marsh woundwort
Duck	Water	hard-mature	Björkelunda	2022	Mint
Duck	Land	hard-immature	Björkelunda	2022	Mint
Duck	Land	hard-mature	Nöbbelöv	2022	Mint
Walk	Land	hard-mature	Nöbbelöv	2022	Mint
Drop	Land	soft-immature	Björkelunda	2022	Mint
Duck	Water	hard-mature	Björkelunda	2022	Mint
Duck	Land	soft-immature	Björkelunda	2022	Mint
Duck	Water	hard-immature	Häckeberga	2022	Mint
Walk	Water	hard-immature	Björkelunda	2022	Mint
Walk	Water	hard-mature	Häckeberga	2022	Mint
Drop	Land	hard-mature	Nöbbelöv	2022	Gypsywort
Drop	Water	soft-immature	Häckeberga	2022	Mint
Drop	Water	soft-immature	Häckeberga	2022	Mint
Drop	Land	soft-immature	Nöbbelöv	2022	Mint
Drop	Land	hard-immature	Häckeberga	2022	Marsh woundwort
Duck	Water	hard-immature	Björkelunda	2022	Mint
Duck	Water	soft-immature	Häckeberga	2022	Mint
Drop	Water	hard-immature	Björkelunda	2022	Mint
Duck	Water	hard-mature	Häckeberga	2022	Mint
Duck	Water	hard-immature	Björkelunda	2022	Mint
Drop	Water	soft-immature	Häckeberga	2022	Mint
Duck	Water	hard-mature	Häckeberga	2022	Mint
Drop	Land	hard-immature	Nöbbelöv	2022	Mint
Duck	Water	hard-mature	Häckeberga	2022	Mint
Duck	Water	hard-mature	Häckeberga	2022	Marsh woundwort
Drop	Land	hard-mature	Björkelunda	2022	Mint
Drop	Water	soft-immature	Häckeberga	2022	Mint
Drop	Water	hard-immature	Häckeberga	2022	Mint
Drop	Water	soft-immature	Häckeberga	2022	Mint
Duck	Water	hard-mature	Häckeberga	2022	Mint
Drop	Land	hard-immature	Häckeberga	2022	Mint
Duck	Water	hard-mature	Häckeberga	2022	Mint
Drop	Land	hard-immature	Häckeberga	2022	Marsh woundwort
Duck	Water	hard-mature	Björkelunda	2022	Mint
Duck	Land	soft-immature	Nöbbelöv	2022	Mint
Duck	Water	hard-immature	Björkelunda	2022	Mint

Duck	Water	soft-immature	Häckeberga	2022	Mint
Drop	Water	soft-immature	Häckeberga	2022	Mint
Walk	Land	soft-immature	Nöbbelöv	2022	Gypsywort
Drop	Water	hard-mature	Häckeberga	2022	Mint
Drop	Water	soft-immature	Häckeberga	2022	Mint
Duck	Water	soft-immature	Björkelunda	2022	Mint
Duck	Land	hard-immature	Häckeberga	2022	Mint
Drop	Land	hard-immature	Björkelunda	2022	Mint
Drop	Land	hard-mature	Björkelunda	2022	Mint
Drop	Water	soft-immature	Björkelunda	2022	Mint
Duck	Water	hard-mature	Björkelunda	2022	Mint
Drop	Land	soft-immature	Häckeberga	2022	Mint
Duck	Water	soft-immature	Häckeberga	2022	Mint
Duck	Water	hard-mature	Björkelunda	2022	Mint
Walk	Land	hard-immature	Häckeberga	2022	Mint
Drop	Land	hard-mature	Nöbbelöv	2022	Gypsywort
Duck	Water	hard-mature	Björkelunda	2022	Mint
Duck	Land	hard-immature	Björkelunda	2022	Mint
Drop	Water	hard-mature	Häckeberga	2022	Marsh woundwort
Drop	Water	soft-immature	Björkelunda	2022	Mint
Duck	Land	hard-mature	Nöbbelöv	2022	Gypsywort
Drop	Water	hard-immature	Häckeberga	2022	Mint
Drop	Land	hard-mature	Häckeberga	2022	Mint
Drop	Land	soft-immature	Björkelunda	2022	Mint
Duck	Water	hard-mature	Häckeberga	2022	Mint
Drop	Land	hard-mature	Nöbbelöv	2022	Gypsywort
Walk	Water	hard-mature	Björkelunda	2022	Mint
Drop	Water	soft-immature	Häckeberga	2022	Mint
Drop	Water	hard-immature	Björkelunda	2022	Mint
Duck	Water	hard-immature	Björkelunda	2022	Mint
Duck	Water	hard-mature	Björkelunda	2022	Mint
Drop	Water	hard-immature	Björkelunda	2022	Mint
Drop	Land	hard-mature	Björkelunda	2022	Mint
Drop	Water	soft-immature	Häckeberga	2022	Marsh woundwort
Drop	Land	hard-immature	Häckeberga	2022	Mint
Drop	Water	soft-immature	Häckeberga	2022	Marsh woundwort
Drop	Water	hard-mature	Nöbbelöv	2022	Mint
Walk	Land	hard-immature	Nöbbelöv	2022	Gypsywort
Duck	Land	hard-immature	Björkelunda	2022	Mint
Duck	Water	soft-immature	Björkelunda	2022	Mint
Drop	Water	soft-immature	Häckeberga	2022	Mint
Duck	Water	hard-mature	Häckeberga	2022	Marsh woundwort
Drop	Water	soft-immature	Häckeberga	2022	Mint
Drop	Land	soft-immature	Nöbbelöv	2022	Gypsywort
Duck	Land	soft-immature	Nöbbelöv	2022	Mint
Drop	Land	hard-mature	Nöbbelöv	2022	Gypsywort
Duck	Water	soft-immature	Björkelunda	2022	Mint
Duck	Water	hard-mature	Björkelunda	2022	Mint

Duck	Water	hard-mature	Björkelunda	2022	Mint
Duck	Water	soft-immature	Björkelunda	2022	Mint
Drop	Water	hard-immature	Häckeberga	2022	Mint
Drop	Water	hard-mature	Häckeberga	2022	Marsh woundwort
Drop	Land	soft-immature	Nöbbelöv	2022	Gypsywort
Duck	Land	hard-mature	Björkelunda	2022	Mint
Walk	Land	hard-immature	Nöbbelöv	2022	Mint
Drop	Land	soft-immature	Björkelunda	2022	Mint
Duck	Water	hard-mature	Häckeberga	2022	Mint
Drop	Water	hard-immature	Björkelunda	2022	Mint
Duck	Water	soft-immature	Björkelunda	2022	Mint
Duck	Water	hard-mature	Björkelunda	2022	Mint
Duck	Land	hard-mature	Nöbbelöv	2022	Mint
Drop	Water	hard-immature	Häckeberga	2022	Marsh woundwort
Duck	Water	hard-mature	Björkelunda	2022	Mint
Walk	Land	hard-mature	Nöbbelöv	2022	Gypsywort
Duck	Water	hard-immature	Häckeberga	2022	Mint
Duck	Land	hard-immature	Nöbbelöv	2022	Gypsywort
Duck	Water	hard-immature	Björkelunda	2022	Mint
Walk	Land	hard-mature	Nöbbelöv	2022	Mint
Walk	Land	hard-immature	Nöbbelöv	2022	Gypsywort
Duck	Land	hard-mature	Björkelunda	2022	Mint
Drop	Water	soft-immature	Häckeberga	2022	Mint
Duck	Water	hard-mature	Björkelunda	2022	Mint
Duck	Water	hard-mature	Häckeberga	2022	Mint
Duck	Land	soft-immature	Björkelunda	2022	Mint
Drop	Land	hard-mature	Björkelunda	2022	Mint
Drop	Water	hard-immature	Björkelunda	2022	Mint
Walk	Water	hard-mature	Häckeberga	2022	Marsh woundwort
Duck	Water	hard-immature	Häckeberga	2022	Mint
Duck	Water	soft-immature	Häckeberga	2022	Marsh woundwort
Drop	Water	hard-mature	Björkelunda	2022	Mint
Duck	Water	hard-immature	Björkelunda	2022	Mint
Duck	Water	hard-mature	Nöbbelöv	2022	Mint
Drop	Land	hard-immature	Nöbbelöv	2022	Mint
Duck	Water	hard-immature	Björkelunda	2022	Mint
Drop	Land	hard-immature	Nöbbelöv	2022	Gypsywort
Duck	Land	hard-mature	Nöbbelöv	2022	Gypsywort
Duck	Water	hard-immature	Häckeberga	2022	Mint
Duck	Water	hard-mature	Björkelunda	2022	Mint
Duck	Water	hard-immature	Björkelunda	2022	Mint
Duck	Water	hard-mature	Björkelunda	2022	Mint
Duck	Water	hard-mature	Björkelunda	2022	Mint
Duck	Water	hard-mature	Häckeberga	2022	Gypsywort
Walk	Land	soft-immature	Nöbbelöv	2022	Mint
Drop	Land	hard-immature	Björkelunda	2022	Mint
Duck	Water	soft-immature	Häckeberga	2022	Mint
Drop	Water	hard-mature	Häckeberga	2022	Mint

Drop	Land	hard-immature	Nöbbelöv	2022	Mint
Drop	Water	hard-immature	Björkelunda	2022	Mint
Duck	Water	hard-mature	Björkelunda	2022	Mint
Drop	Land	hard-immature	Nöbbelöv	2022	Mint
Duck	Land	hard-immature	Björkelunda	2022	Mint
Drop	Water	hard-immature	Björkelunda	2022	Mint
Duck	Water	hard-mature	Häckeberga	2022	Mint
Duck	Water	hard-mature	Björkelunda	2022	Mint
Drop	Water	hard-mature	Björkelunda	2022	Gypsywort
Duck	Land	hard-mature	Björkelunda	2022	Mint
Duck	Water	hard-immature	Björkelunda	2022	Mint
Drop	Water	hard-immature	Björkelunda	2022	Mint
Duck	Water	hard-mature	Häckeberga	2022	Marsh woundwort
Duck	Water	hard-immature	Häckeberga	2022	Marsh woundwort
Drop	Water	soft-immature	Häckeberga	2022	Marsh woundwort
Drop	Water	hard-immature	Björkelunda	2022	Mint
Duck	Land	hard-immature	Häckeberga	2022	Mint
Duck	Land	hard-immature	Björkelunda	2022	Gypsywort
Duck	Water	soft-immature	Häckeberga	2022	Mint
Duck	Land	hard-immature	Nöbbelöv	2022	Mint
Duck	Water	soft-immature	Häckeberga	2022	Mint
Duck	Water	hard-mature	Björkelunda	2022	Mint
Duck	Water	hard-mature	Häckeberga	2022	Mint
Drop	Land	hard-immature	Björkelunda	2022	Mint
Drop	Water	hard-immature	Björkelunda	2022	Mint
Drop	Land	hard-immature	Nöbbelöv	2022	NA
Duck	Water	hard-mature	Häckeberga	2022	Mint
Duck	Water	hard-mature	Häckeberga	2022	Mint
Duck	Water	hard-mature	Björkelunda	2022	Mint
Drop	Land	hard-mature	Björkelunda	2022	Mint
Drop	Water	hard-immature	Häckeberga	2022	Mint
Drop	Land	hard-immature	Björkelunda	2022	Mint
Drop	Land	hard-immature	Björkelunda	2022	Mint
Walk	Water	hard-immature	Häckeberga	2022	Mint
Duck	Water	soft-immature	Björkelunda	2022	Mint
Duck	Water	hard-immature	Björkelunda	2022	Mint
Duck	Land	hard-mature	Björkelunda	2022	Mint
Drop	Water	hard-immature	Björkelunda	2022	Mint
Drop	Land	soft-immature	Björkelunda	2022	Mint
Drop	Water	soft-immature	Häckeberga	2022	Mint
Walk	Water	hard-mature	Häckeberga	2022	Gypsywort
Drop	Water	soft-immature	Björkelunda	2022	Mint
Duck	Land	hard-mature	Häckeberga	2022	Mint
Duck	Land	hard-mature	Björkelunda	2022	Mint
Duck	Land	hard-immature	Björkelunda	2022	Mint
Drop	Water	hard-immature	Björkelunda	2022	Mint
Duck	Land	hard-immature	Björkelunda	2022	Mint
Walk	Water	hard-mature	Björkelunda	2022	Mint

Duck	Land	hard-mature	Nöbbelöv	2022	Mint
Drop	Water	hard-mature	Björkelunda	2022	Mint
Duck	Water	hard-mature	Björkelunda	2022	Mint
Drop	Water	hard-immature	Björkelunda	2022	Mint
Drop	Land	soft-immature	Björkelunda	2022	Mint
Duck	Water	hard-mature	Björkelunda	2022	Mint
Duck	Water	soft-immature	Häckeberga	2022	Mint
Drop	Water	hard-immature	Nöbbelöv	2022	NA
Duck	Land	hard-immature	Nöbbelöv	2022	Gypsywort
Duck	Water	hard-mature	Björkelunda	2022	Mint
Duck	Water	hard-immature	Björkelunda	2022	Marsh woundwort
Duck	Water	hard-immature	Björkelunda	2022	Mint
Drop	Land	hard-immature	Nöbbelöv	2022	Mint
Walk	Land	soft-immature	Nöbbelöv	2022	Mint
Drop	Water	soft-immature	Björkelunda	2022	Mint
Drop	Land	hard-immature	Björkelunda	2022	Mint
Drop	Land	hard-immature	Björkelunda	2022	Mint
Duck	Water	hard-mature	Björkelunda	2022	Mint
Drop	Land	hard-immature	Björkelunda	2022	Mint
Duck	Water	hard-mature	Häckeberga	2022	Mint
Drop	Water	soft-immature	Häckeberga	2022	Mint
Duck	Water	soft-immature	Häckeberga	2022	Gypsywort
Duck	Water	hard-mature	Björkelunda	2022	Mint
Duck	Water	hard-immature	Häckeberga	2022	Mint
Drop	Water	hard-mature	Häckeberga	2022	Marsh woundwort
Drop	Water	hard-mature	Häckeberga	2022	Marsh woundwort
Duck	Water	hard-mature	Häckeberga	2022	Marsh woundwort
Drop	Land	hard-mature	Björkelunda	2022	Gypsywort
Duck	Land	soft-immature	Björkelunda	2022	Mint
Duck	Water	hard-mature	Björkelunda	2022	Mint
Drop	Land	soft-immature	Nöbbelöv	2022	Gypsywort
Drop	Land	soft-immature	Björkelunda	2022	Mint
Duck	Land	hard-mature	Björkelunda	2022	Mint
Drop	Water	hard-immature	Häckeberga	2022	Marsh woundwort
Duck	Water	hard-mature	Björkelunda	2022	Mint
Drop	Land	soft-immature	Björkelunda	2022	Mint
Duck	Water	hard-mature	Björkelunda	2022	Gypsywort
Drop	Water	hard-immature	Häckeberga	2022	Mint
Drop	Land	hard-immature	Häckeberga	2022	Marsh woundwort
Duck	Water	hard-immature	Björkelunda	2022	Mint
Duck	Land	hard-mature	Björkelunda	2022	Mint
Drop	Water	soft-immature	Häckeberga	2022	Mint
Duck	Water	hard-mature	Björkelunda	2022	Mint
Drop	Water	soft-immature	Häckeberga	2022	Marsh woundwort
Drop	Water	soft-immature	Häckeberga	2022	Mint
Drop	Water	soft-immature	Björkelunda	2022	Mint
Duck	Water	hard-mature	Häckeberga	2022	Mint
Duck	Water	soft-immature	Häckeberga	2022	Mint

Duck	Water	hard-mature	Björkelunda	2022	Mint
Drop	Land	hard-immature	Nöbbelöv	2022	Mint
Drop	Water	soft-immature	Häckeberga	2022	Gypsywort
Drop	Land	soft-immature	Nöbbelöv	2022	Mint
Duck	Water	hard-mature	Häckeberga	2022	Mint
Duck	Water	hard-mature	Björkelunda	2022	Mint
Duck	Water	hard-mature	Häckeberga	2022	Marsh woundwort
Drop	Water	soft-immature	Häckeberga	2022	Marsh woundwort
Drop	Water	hard-mature	Häckeberga	2022	Marsh woundwort
Duck	Water	hard-mature	Häckeberga	2022	Mint
Drop	Land	soft-immature	Björkelunda	2022	Mint
Drop	Water	soft-immature	Nöbbelöv	2022	Mint
Duck	Land	hard-immature	Nöbbelöv	2022	Gypsywort
Walk	Water	soft-immature	Häckeberga	2022	Mint
Walk	Water	soft-immature	Björkelunda	2022	Mint
Duck	Land	hard-mature	Nöbbelöv	2022	Gypsywort
Walk	Land	hard-mature	Björkelunda	2022	Mint
Walk	Land	hard-immature	Nöbbelöv	2022	NA
Duck	Water	hard-mature	Björkelunda	2022	Mint
Duck	Water	hard-immature	Häckeberga	2022	Mint
Duck	Water	hard-mature	Häckeberga	2022	Mint
Drop	Water	soft-immature	Björkelunda	2022	Mint
Drop	Water	hard-immature	Björkelunda	2022	Mint
Drop	Water	hard-mature	Björkelunda	2022	Mint
Duck	Water	hard-mature	Häckeberga	2022	Mint
Drop	Water	soft-immature	Häckeberga	2022	Mint
Duck	Water	hard-mature	Björkelunda	2022	Mint
Walk	Land	hard-mature	Björkelunda	2022	Mint
Duck	Water	hard-mature	Häckeberga	2022	Mint
Duck	Water	hard-mature	Björkelunda	2022	Mint
Drop	Water	hard-mature	Häckeberga	2022	Mint
Drop	Land	hard-mature	Björkelunda	2022	Mint
Drop	Water	soft-immature	Björkelunda	2022	Mint
Drop	Land	hard-immature	Nöbbelöv	2022	Gypsywort
Drop	Water	hard-immature	Häckeberga	2022	Mint
Duck	Water	hard-mature	Björkelunda	2022	Mint
Duck	Water	soft-immature	Björkelunda	2022	Mint
Drop	Water	soft-immature	Häckeberga	2022	Mint
Drop	Water	soft-immature	Häckeberga	2022	Mint
Duck	Water	hard-mature	Häckeberga	2022	Mint
Duck	Water	soft-immature	Häckeberga	2022	Mint
Drop	Water	soft-immature	Häckeberga	2022	Marsh woundwort
Drop	Water	hard-mature	Björkelunda	2022	Mint
Duck	Land	hard-mature	Björkelunda	2022	Mint
Duck	Water	hard-mature	Björkelunda	2022	Mint
Duck	Water	hard-mature	Nöbbelöv	2022	Mint
Duck	Water	hard-immature	Björkelunda	2022	Mint
Duck	Water	hard-mature	Häckeberga	2022	Mint

Drop	Land	hard-immature	Björkelunda	2022	Mint
Walk	Land	hard-mature	Nöbbelöv	2022	Mint
Duck	Land	hard-mature	Nöbbelöv	2022	Mint
Duck	Water	hard-immature	Björkelunda	2022	Mint
Drop	Water	hard-mature	Häckeberga	2022	NA
Drop	Land	hard-immature	Nöbbelöv	2022	Gypsywort
Duck	Water	soft-immature	Häckeberga	2022	Marsh woundwort
Drop	Water	soft-immature	Häckeberga	2022	Mint
Drop	Water	hard-immature	Björkelunda	2022	Mint
Duck	Water	soft-immature	Häckeberga	2022	Mint
Duck	Water	hard-immature	Björkelunda	2022	Mint
Drop	Land	hard-immature	Nöbbelöv	2022	NA
Duck	Water	hard-immature	Björkelunda	2022	Mint
Duck	Land	soft-immature	Nöbbelöv	2022	Gypsywort
Drop	Water	hard-immature	Nöbbelöv	2022	Mint
Drop	Land	hard-immature	Björkelunda	2022	Mint
Drop	Land	hard-mature	Björkelunda	2022	Mint
Duck	Water	soft-immature	Häckeberga	2022	Mint
Duck	Water	hard-mature	Björkelunda	2022	Mint
Duck	Water	hard-mature	Häckeberga	2022	Mint
Duck	Water	hard-mature	Björkelunda	2022	Mint
Drop	Water	soft-immature	Björkelunda	2022	Mint
Duck	Water	hard-immature	Björkelunda	2022	Mint
Duck	Water	hard-immature	Häckeberga	2022	Gypsywort
Duck	Land	hard-mature	Nöbbelöv	2022	Gypsywort
Drop	Water	hard-mature	Björkelunda	2022	Mint
Duck	Land	hard-immature	Nöbbelöv	2022	Mint
Drop	Water	hard-immature	Björkelunda	2022	Mint
Duck	Land	hard-mature	Björkelunda	2022	Gypsywort
Duck	Water	hard-mature	Björkelunda	2022	Mint
Duck	Water	soft-immature	Häckeberga	2022	Mint
Duck	Land	hard-immature	Björkelunda	2022	Mint
Duck	Water	soft-immature	Häckeberga	2022	Mint
Duck	Water	hard-mature	Björkelunda	2022	Mint
Duck	Water	hard-mature	Nöbbelöv	2022	Mint
Drop	Land	hard-mature	Nöbbelöv	2022	Mint
Drop	Land	soft-immature	Björkelunda	2022	Mint
Walk	Water	hard-immature	Björkelunda	2022	Mint
Duck	Water	hard-mature	Häckeberga	2022	Mint
Duck	Land	hard-mature	Nöbbelöv	2022	Mint
Walk	Water	soft-immature	Häckeberga	2022	Gypsywort
Duck	Land	hard-immature	Nöbbelöv	2022	Gypsywort
Duck	Water	hard-immature	Björkelunda	2022	Mint
Duck	Water	hard-mature	Björkelunda	2022	Mint
Drop	Water	hard-immature	Björkelunda	2022	Mint
Duck	Land	hard-mature	Nöbbelöv	2022	Mint
Duck	Water	hard-immature	Björkelunda	2022	Mint
Duck	Land	hard-immature	Nöbbelöv	2022	NA

Duck	Land	soft-immature	Häckeberga	2022	Mint
Walk	Land	soft-immature	Björkelunda	2022	Mint
Drop	Water	hard-mature	Häckeberga	2022	Marsh woundwort
Duck	Water	hard-immature	Björkelunda	2022	Mint
Duck	Water	hard-immature	Björkelunda	2022	Mint
Drop	Land	hard-immature	Björkelunda	2022	Mint
Drop	Land	hard-immature	Björkelunda	2022	Mint
Drop	Land	hard-mature	Häckeberga	2022	Mint
Drop	Water	hard-immature	Häckeberga	2022	Marsh woundwort
Drop	Land	hard-mature	Häckeberga	2022	Mint
Duck	Water	hard-mature	Björkelunda	2022	Gypsywort
Duck	Land	soft-immature	Björkelunda	2022	Mint
Drop	Water	soft-immature	Häckeberga	2022	Mint
Drop	Water	hard-immature	Björkelunda	2022	Mint
Duck	Water	hard-mature	Häckeberga	2022	Gypsywort
Duck	Land	hard-mature	Björkelunda	2022	Mint
Duck	Water	soft-immature	Häckeberga	2022	Mint
Drop	Water	soft-immature	Häckeberga	2022	Mint
Duck	Water	hard-mature	Björkelunda	2022	Marsh woundwort
Duck	Water	hard-immature	Björkelunda	2022	Mint
Walk	Water	hard-mature	Björkelunda	2022	Marsh woundwort
Duck	Water	hard-immature	Nöbbelöv	2022	Mint
Drop	Water	hard-immature	Häckeberga	2022	Mint
Duck	Land	hard-immature	Häckeberga	2022	Mint
Duck	Water	soft-immature	Häckeberga	2022	Mint
Drop	Water	hard-mature	Häckeberga	2022	Mint
Duck	Water	hard-mature	Björkelunda	2022	Mint
Duck	Water	hard-immature	Björkelunda	2022	Mint
Duck	Water	soft-immature	Häckeberga	2022	Mint
Walk	Water	hard-mature	Björkelunda	2022	Mint
Duck	Land	hard-immature	Nöbbelöv	2022	Mint
Duck	Water	soft-immature	Björkelunda	2022	Mint
Drop	Land	soft-immature	Nöbbelöv	2022	Gypsywort
Duck	Water	hard-immature	Häckeberga	2022	Mint
Walk	Water	soft-immature	Häckeberga	2022	Marsh woundwort
Duck	Water	hard-mature	Häckeberga	2022	Mint
Duck	Water	hard-immature	Häckeberga	2022	Gypsywort
Duck	Water	hard-immature	Häckeberga	2022	Marsh woundwort
Drop	Land	soft-immature	Björkelunda	2022	Gypsywort
Drop	Land	hard-mature	Björkelunda	2022	NA
Duck	Water	hard-mature	Björkelunda	2022	Mint
Drop	Water	soft-immature	Häckeberga	2022	Mint
Drop	Land	hard-mature	Björkelunda	2022	Mint
Duck	Water	hard-mature	Björkelunda	2022	Mint
Drop	Land	hard-immature	Nöbbelöv	2022	Gypsywort
Walk	Water	hard-mature	Björkelunda	2022	Mint
Duck	Land	soft-immature	Björkelunda	2022	Mint
Drop	Land	hard-mature	Björkelunda	2022	Mint

Drop	Land	hard-immature	Björkelunda	2022	Mint
Duck	Water	hard-immature	Björkelunda	2022	Mint
Duck	Land	hard-mature	Björkelunda	2022	Gypsywort
Duck	Water	hard-mature	Häckeberga	2022	Mint
Drop	Land	hard-immature	Häckeberga	2022	Marsh woundwort
Duck	Land	hard-mature	Björkelunda	2022	Mint
Walk	Water	hard-mature	Björkelunda	2022	Mint
Duck	Land	soft-immature	Häckeberga	2022	Mint
Duck	Water	hard-mature	Björkelunda	2022	Mint
Drop	Land	hard-immature	Björkelunda	2022	Mint
Drop	Land	hard-immature	Björkelunda	2022	Mint
Drop	Water	soft-immature	Björkelunda	2022	Mint
Drop	Water	hard-mature	Häckeberga	2022	Mint
Duck	Water	hard-immature	Björkelunda	2022	Mint
Duck	Water	hard-mature	Häckeberga	2022	Mint
Drop	Land	soft-immature	Björkelunda	2022	Mint
Duck	Land	soft-immature	Björkelunda	2022	Mint
Walk	Water	soft-immature	Björkelunda	2022	Mint
Duck	Land	soft-immature	Björkelunda	2022	Mint
Duck	Water	soft-immature	Häckeberga	2022	Mint
Drop	Land	soft-immature	Häckeberga	2022	Mint
Duck	Water	soft-immature	Björkelunda	2022	Mint
Walk	Water	soft-immature	Björkelunda	2022	Mint
Walk	Land	soft-immature	Björkelunda	2022	Mint
Drop	Land	soft-immature	Nöbbelöv	2022	Mint
Drop	Land	soft-immature	Nöbbelöv	2022	Mint
Drop	Water	soft-immature	Björkelunda	2022	Mint
Walk	Water	soft-immature	Björkelunda	2022	Mint
Duck	Water	hard-immature	Björkelunda	2022	Mint
Duck	Water	soft-immature	Björkelunda	2022	Mint
Walk	Water	soft-immature	Björkelunda	2022	Mint
Duck	Land	soft-immature	Björkelunda	2022	Mint
Drop	Land	soft-immature	Björkelunda	2022	Mint
Drop	Land	soft-immature	Nöbbelöv	2022	Mint
Drop	Land	soft-immature	Nöbbelöv	2022	Mint
Duck	Land	soft-immature	Nöbbelöv	2022	Mint
Drop	Land	soft-immature	Nöbbelöv	2022	Mint
Drop	Land	soft-immature	Nöbbelöv	2022	Mint
Duck	Land	soft-immature	Nöbbelöv	2022	Mint
Drop	Land	soft-immature	Nöbbelöv	2022	Mint
Duck	Land	soft-immature	Nöbbelöv	2022	NA
Drop	Land	soft-immature	Nöbbelöv	2022	NA
Duck	Land	soft-immature	Nöbbelöv	2022	Mint
Drop	Land	soft-immature	Nöbbelöv	2022	Mint
Drop	Water	soft-immature	Nöbbelöv	2022	Mint
Drop	Water	soft-immature	Nöbbelöv	2022	Mint
Drop	Water	soft-immature	Nöbbelöv	2022	Mint
Drop	Water	soft-immature	Nöbbelöv	2022	Mint

Walk	Land	soft-immature	Nöbbelöv	2022	Mint
Drop	Land	soft-immature	Nöbbelöv	2022	Mint
Duck	Land	soft-immature	Nöbbelöv	2022	Mint
Duck	Land	soft-immature	Nöbbelöv	2022	Mint
Drop	Land	soft-immature	Nöbbelöv	2022	Mint
Duck	Water	soft-immature	Häckeberga	2022	Mint
Duck	Water	soft-immature	Häckeberga	2022	Mint
Drop	Land	soft-immature	Häckeberga	2022	Mint
Duck	Water	soft-immature	Häckeberga	2022	Mint
Duck	Water	soft-immature	Häckeberga	2022	Mint
Duck	Water	soft-immature	Häckeberga	2022	Mint
Duck	Water	soft-immature	Häckeberga	2022	Mint
Duck	Water	soft-immature	Häckeberga	2022	Mint
Drop	Land	soft-immature	Häckeberga	2022	Mint
Drop	Water	soft-immature	Häckeberga	2022	Mint
Drop	Water	soft-immature	Häckeberga	2022	Mint
Drop	Water	soft-immature	Häckeberga	2022	Mint
Drop	Water	soft-immature	Häckeberga	2022	Marsh woundwort
Duck	Water	soft-immature	Häckeberga	2022	Mint
Drop	Water	soft-immature	Häckeberga	2022	Mint
Walk	Water	soft-immature	Häckeberga	2022	Mint
Duck	Water	soft-immature	Häckeberga	2022	Mint
Duck	Water	soft-immature	Häckeberga	2022	Marsh woundwort
Duck	Water	soft-immature	Häckeberga	2022	Mint
Duck	Water	soft-immature	Häckeberga	2022	Mint
Drop	Water	soft-immature	Häckeberga	2022	Mint
Duck	Water	soft-immature	Häckeberga	2022	Mint
Drop	Water	soft-immature	Häckeberga	2022	Mint
Duck	Water	soft-immature	Häckeberga	2022	Mint
Duck	Water	soft-immature	Häckeberga	2022	Mint
Duck	Water	soft-immature	Häckeberga	2022	Mint
Duck	Water	soft-immature	Häckeberga	2022	Mint
Duck	Water	soft-immature	Häckeberga	2022	Mint
Duck	Water	soft-immature	Häckeberga	2022	Mint
Duck	Water	soft-immature	Häckeberga	2022	Mint
Duck	Water	soft-immature	Häckeberga	2022	Mint
Walk	Water	soft-immature	Häckeberga	2022	Mint
Drop	Water	soft-immature	Björkelunda	2022	Mint
Drop	Water	soft-immature	Björkelunda	2022	Mint
Duck	Land	hard-immature	Nöbbelöv	2022	Mint
Drop	Land	hard-immature	Nöbbelöv	2022	Mint
Drop	Land	soft-immature	Nöbbelöv	2022	Mint
Drop	Land	soft-immature	Nöbbelöv	2022	Mint
Walk	Land	soft-immature	Nöbbelöv	2022	Mint
Duck	Land	soft-immature	Nöbbelöv	2022	Gypsywort
Walk	Land	soft-immature	Nöbbelöv	2022	Gypsywort
Duck	Land	soft-immature	Nöbbelöv	2022	NA
Duck	Water	soft-immature	Häckeberga	2022	Mint

Drop	Water	soft-immature	Häckeberga	2022	Marsh woundwort
Drop	Water	hard-immature	Häckeberga	2022	Mint
Duck	Water	soft-immature	Häckeberga	2022	Mint
Drop	Water	hard-immature	Häckeberga	2022	Mint
Drop	Water	soft-immature	Häckeberga	2022	Marsh woundwort
Drop	Water	soft-immature	Häckeberga	2022	Mint
Drop	Water	soft-immature	Häckeberga	2022	Mint
Drop	Water	soft-immature	Häckeberga	2022	Mint
Drop	Water	soft-immature	Häckeberga	2022	Mint
Drop	Water	soft-immature	Häckeberga	2022	Mint
Duck	Water	soft-immature	Häckeberga	2022	Marsh woundwort
Duck	Water	soft-immature	Häckeberga	2022	Gypsywort
Drop	Water	soft-immature	Häckeberga	2022	Mint
Drop	Water	soft-immature	Häckeberga	2022	Mint
Drop	Water	soft-immature	Häckeberga	2022	Mint
Duck	Water	soft-immature	Häckeberga	2022	Gypsywort
Drop	Land	soft-immature	Häckeberga	2022	Gypsywort
Drop	Water	soft-immature	Häckeberga	2022	Mint
Duck	Water	soft-immature	Häckeberga	2022	Mint
Drop	Water	soft-immature	Häckeberga	2022	Mint
Duck	Water	soft-immature	Häckeberga	2022	Gypsywort
Walk	Water	soft-immature	Häckeberga	2022	Mint
Duck	Water	soft-immature	Häckeberga	2022	Gypsywort
Drop	Water	soft-immature	Häckeberga	2022	Mint
Duck	Water	soft-immature	Häckeberga	2022	Marsh woundwort
Duck	Water	soft-immature	Häckeberga	2022	Marsh woundwort
Duck	Water	soft-immature	Häckeberga	2022	Marsh woundwort
Drop	Water	soft-immature	Häckeberga	2022	Marsh woundwort
Duck	Water	soft-immature	Häckeberga	2022	Marsh woundwort
Duck	Water	soft-immature	Häckeberga	2022	Marsh woundwort
Duck	Water	soft-immature	Häckeberga	2022	Marsh woundwort
Drop	Land	soft-immature	Häckeberga	2022	Mint
Drop	Water	soft-immature	Häckeberga	2022	Marsh woundwort
Drop	Land	soft-immature	Häckeberga	2022	Marsh woundwort
Drop	Water	soft-immature	Häckeberga	2022	Marsh woundwort
Duck	Land	soft-immature	Häckeberga	2022	Marsh woundwort
Drop	Land	soft-immature	Häckeberga	2022	Marsh woundwort
Drop	Land	soft-immature	Häckeberga	2022	Marsh woundwort
Duck	Water	soft-immature	Häckeberga	2022	Mint
Drop	Water	soft-immature	Häckeberga	2022	Mint
Drop	Water	soft-immature	Häckeberga	2022	Mint
Drop	Water	soft-immature	Häckeberga	2022	Mint
Drop	Water	soft-immature	Häckeberga	2022	Mint
Drop	Water	soft-immature	Häckeberga	2022	Mint
Drop	Land	soft-immature	Häckeberga	2022	Mint
Drop	Water	soft-immature	Häckeberga	2022	Mint
Drop	Water	soft-immature	Häckeberga	2022	Mint
Drop	Water	soft-immature	Häckeberga	2022	Mint

Walk	Water	soft-immature	Häckeberga	2022	Mint
Duck	Water	soft-immature	Häckeberga	2022	Mint
Drop	Water	soft-immature	Häckeberga	2022	Mint
Drop	Water	soft-immature	Häckeberga	2022	Mint
Duck	Water	soft-immature	Häckeberga	2022	Mint
Duck	Water	soft-immature	Häckeberga	2022	Mint
Duck	Water	soft-immature	Häckeberga	2022	Mint
Duck	Water	soft-immature	Björkelunda	2022	Mint
Duck	Water	soft-immature	Björkelunda	2022	Mint
Duck	Water	soft-immature	Björkelunda	2022	Mint
Duck	Water	soft-immature	Björkelunda	2022	Mint
Duck	Water	soft-immature	Björkelunda	2022	Mint
Duck	Water	hard-immature	Björkelunda	2022	Mint
Duck	Water	hard-immature	Björkelunda	2022	Mint
Duck	Water	soft-immature	Björkelunda	2022	Mint
Drop	Water	soft-immature	Björkelunda	2022	Mint
Drop	Water	soft-immature	Björkelunda	2022	Mint
Drop	Water	soft-immature	Björkelunda	2022	Mint
Duck	Water	soft-immature	Björkelunda	2022	Mint
Drop	Water	soft-immature	Björkelunda	2022	Mint
Duck	Water	soft-immature	Björkelunda	2022	Mint
Duck	Water	soft-immature	Björkelunda	2022	Mint
Drop	Water	soft-immature	Björkelunda	2022	Mint
Duck	Water	hard-immature	Björkelunda	2022	Mint
Drop	Water	hard-immature	Björkelunda	2022	Mint
Duck	Water	soft-immature	Björkelunda	2022	Mint
Duck	Water	hard-immature	Björkelunda	2022	Mint
Duck	Land	hard-immature	Björkelunda	2022	Marsh woundwort
Drop	Land	soft-immature	Björkelunda	2022	Mint
Drop	Water	soft-immature	Björkelunda	2022	Marsh woundwort
Duck	Water	soft-immature	Björkelunda	2022	Mint
Drop	Water	soft-immature	Björkelunda	2022	Mint
Duck	Water	soft-immature	Björkelunda	2022	Mint
Drop	Land	soft-immature	Björkelunda	2022	Mint
Drop	Land	hard-immature	Björkelunda	2022	Mint
Drop	Land	soft-immature	Björkelunda	2022	Mint
Drop	Water	soft-immature	Björkelunda	2022	Mint
Drop	Land	soft-immature	Björkelunda	2022	Mint
Drop	Land	hard-immature	Björkelunda	2022	Mint
Drop	Land	soft-immature	Björkelunda	2022	Mint
Duck	Land	hard-immature	Nöbbelöv	2022	Mint
Duck	Land	hard-immature	Nöbbelöv	2022	Mint
Duck	Land	soft-immature	Nöbbelöv	2022	Mint
Walk	Land	soft-immature	Nöbbelöv	2022	Mint
Duck	Water	soft-immature	Nöbbelöv	2022	Gypsywort
Duck	Land	hard-immature	Nöbbelöv	2022	NA
Drop	Land	hard-immature	Nöbbelöv	2022	Mint
Drop	Water	hard-immature	Nöbbelöv	2022	NA

Drop	Land	hard-immature	Nöbbelöv	2022	NA
Duck	Land	soft-immature	Nöbbelöv	2022	Gypsywort
Walk	Water	hard-immature	Nöbbelöv	2022	Mint
Duck	Water	soft-immature	Nöbbelöv	2022	Mint
Walk	Land	soft-immature	Nöbbelöv	2022	Gypsywort
Drop	Land	hard-immature	Björkelunda	2022	NA
Drop	Land	soft-immature	Björkelunda	2022	Mint
Walk	Land	soft-immature	Björkelunda	2022	Mint
Drop	Land	hard-immature	Björkelunda	2022	Mint
Walk	Land	hard-immature	Björkelunda	2022	Gypsywort
Drop	Water	soft-immature	Häckeberga	2022	Mint
Drop	Water	hard-immature	Häckeberga	2022	Marsh woundwort
Duck	Water	soft-immature	Häckeberga	2022	Mint
Duck	Water	soft-immature	Häckeberga	2022	Mint
Drop	Water	soft-immature	Häckeberga	2022	Mint
Walk	Water	soft-immature	Häckeberga	2022	Marsh woundwort
Duck	Water	soft-immature	Häckeberga	2022	Mint
Drop	Water	hard-immature	Häckeberga	2022	Marsh woundwort
Drop	Water	soft-immature	Häckeberga	2022	Marsh woundwort
Duck	Water	soft-immature	Häckeberga	2022	Mint
Duck	Water	soft-immature	Häckeberga	2022	Mint
Drop	Water	soft-immature	Häckeberga	2022	Mint
Drop	Water	soft-immature	Björkelunda	2022	Mint
Duck	Water	soft-immature	Häckeberga	2022	Mint
Drop	Water	soft-immature	Häckeberga	2022	Mint
Drop	Water	soft-immature	Häckeberga	2022	Mint
Duck	Water	soft-immature	Häckeberga	2022	Marsh woundwort
Drop	Water	soft-immature	Häckeberga	2022	Mint
Duck	Water	soft-immature	Häckeberga	2022	Gypsywort
Drop	Water	soft-immature	Häckeberga	2022	Mint
Drop	Water	soft-immature	Häckeberga	2022	Marsh woundwort
Drop	Land	soft-immature	Häckeberga	2022	Mint
Drop	Land	soft-immature	Häckeberga	2022	Mint
Drop	Water	hard-immature	Häckeberga	2022	Mint
Drop	Water	soft-immature	Häckeberga	2022	Mint
Drop	Water	soft-immature	Häckeberga	2022	Mint
Duck	Water	soft-immature	Häckeberga	2022	Gypsywort
Duck	Land	soft-immature	Häckeberga	2022	Gypsywort
Duck	Water	soft-immature	Häckeberga	2022	Gypsywort
Drop	Water	hard-immature	Häckeberga	2022	Marsh woundwort
Drop	Water	soft-immature	Häckeberga	2022	Mint
Duck	Water	soft-immature	Häckeberga	2022	Marsh woundwort
Duck	Water	soft-immature	Häckeberga	2022	Marsh woundwort
Drop	Water	soft-immature	Häckeberga	2022	Mint
Duck	Water	soft-immature	Häckeberga	2022	Mint
Walk	Water	soft-immature	Häckeberga	2022	Mint
Drop	Water	soft-immature	Häckeberga	2022	Mint
Duck	Water	hard-immature	Häckeberga	2022	Mint

Walk	Land	soft-immature	Häckeberga	2022	Mint
Drop	Land	soft-immature	Nöbbelöv	2022	Mint
Drop	Land	soft-immature	Nöbbelöv	2022	Mint
Duck	Land	soft-immature	Häckeberga	2022	Mint
Duck	Water	soft-immature	Häckeberga	2022	Mint
Duck	Water	soft-immature	Häckeberga	2022	Marsh woundwort
Walk	Water	soft-immature	Häckeberga	2022	Marsh woundwort
Duck	Water	soft-immature	Häckeberga	2022	Gypsywort
Duck	Water	soft-immature	Björkelunda	2022	Mint
Drop	Water	soft-immature	Björkelunda	2022	Mint
Duck	Water	soft-immature	Björkelunda	2022	Mint
Duck	Water	hard-immature	Björkelunda	2022	Mint
Drop	Water	hard-immature	Björkelunda	2022	Mint
Walk	Water	soft-immature	Björkelunda	2022	Mint
Drop	Water	soft-immature	Björkelunda	2022	Mint
Duck	Water	hard-immature	Björkelunda	2022	Mint
Duck	Water	hard-immature	Björkelunda	2022	Mint
Duck	Water	soft-immature	Björkelunda	2022	Mint
Walk	Water	soft-immature	Björkelunda	2022	Mint
Drop	Water	soft-immature	Björkelunda	2022	Mint
Drop	Water	hard-immature	Björkelunda	2022	Mint
Drop	Water	hard-immature	Björkelunda	2022	Mint
Drop	Water	soft-immature	Björkelunda	2022	Mint
Drop	Water	soft-immature	Björkelunda	2022	Mint
Drop	Water	soft-immature	Björkelunda	2022	Mint
Duck	Water	hard-immature	Björkelunda	2022	Mint
Drop	Water	soft-immature	Björkelunda	2022	Mint
Duck	Water	hard-immature	Björkelunda	2022	Mint
Drop	Water	soft-immature	Björkelunda	2022	Mint
Duck	Water	soft-immature	Björkelunda	2022	Mint
Drop	Water	soft-immature	Björkelunda	2022	Mint
Duck	Water	soft-immature	Björkelunda	2022	Mint
Duck	Water	soft-immature	Björkelunda	2022	Mint
Duck	Water	hard-immature	Björkelunda	2022	Mint
Drop	Land	soft-immature	Nöbbelöv	2022	Gypsywort
Drop	Land	hard-immature	Nöbbelöv	2022	Gypsywort
Drop	Land	soft-immature	Nöbbelöv	2022	Gypsywort
Drop	Land	soft-immature	Nöbbelöv	2022	Gypsywort
Drop	Land	hard-immature	Nöbbelöv	2022	Mint
Duck	Water	soft-immature	Häckeberga	2022	Mint
Drop	Land	hard-immature	Nöbbelöv	2022	Mint
Drop	Land	hard-immature	Nöbbelöv	2022	Gypsywort
Duck	Water	hard-immature	Häckeberga	2022	Mint
Duck	Water	soft-immature	Häckeberga	2022	Mint
Duck	Water	soft-immature	Häckeberga	2022	Mint
Drop	Water	soft-immature	Häckeberga	2022	Mint
Duck	Water	hard-immature	Häckeberga	2022	Mint
Duck	Water	hard-immature	Häckeberga	2022	Marsh woundwort

Duck	Water	hard-immature	Björkelunda	2022	Mint
Duck	Land	hard-immature	Björkelunda	2022	Mint
Walk	Water	hard-mature	Björkelunda	2022	Mint
Drop	Water	hard-immature	Björkelunda	2022	Mint
Duck	Land	hard-immature	Björkelunda	2022	Mint
Duck	Water	soft-immature	Björkelunda	2022	Mint
Duck	Water	soft-immature	Björkelunda	2022	Mint
Duck	Water	soft-immature	Häckeberga	2022	Mint
Duck	Land	hard-immature	Björkelunda	2022	NA
Drop	Water	soft-immature	Björkelunda	2022	Mint
Duck	Water	soft-immature	Björkelunda	2022	Mint
Duck	Water	hard-immature	Björkelunda	2022	Mint
Duck	Water	hard-immature	Björkelunda	2022	Mint
Drop	Water	soft-immature	Björkelunda	2022	Mint
Duck	Water	soft-immature	Björkelunda	2022	Mint
Duck	Water	soft-immature	Björkelunda	2022	Mint
Drop	Water	soft-immature	Björkelunda	2022	Mint
Drop	Water	soft-immature	Björkelunda	2022	Mint
Drop	Water	soft-immature	Björkelunda	2022	Mint
Duck	Water	hard-immature	Björkelunda	2022	Mint
Duck	Water	hard-immature	Björkelunda	2022	Mint
Drop	Land	hard-immature	Nöbbelöv	2022	Gypsywort
Drop	Land	soft-immature	Nöbbelöv	2022	Gypsywort
Drop	Land	soft-immature	Nöbbelöv	2022	Gypsywort
Drop	Land	soft-immature	Nöbbelöv	2022	Gypsywort
Drop	Land	soft-immature	Nöbbelöv	2022	Gypsywort
Drop	Water	soft-immature	Häckeberga	2022	Gypsywort
Duck	Land	hard-immature	Häckeberga	2022	Marsh woundwort
Drop	Water	hard-immature	Häckeberga	2022	Mint
Duck	Water	hard-immature	Häckeberga	2022	Mint
Duck	Water	hard-immature	Häckeberga	2022	Mint
Walk	Water	hard-immature	Häckeberga	2022	Marsh woundwort
Drop	Land	hard-immature	Häckeberga	2022	Mint
Drop	Land	soft-immature	Björkelunda	2022	Mint
Drop	Land	hard-immature	Björkelunda	2022	Mint
Drop	Water	hard-immature	Björkelunda	2022	Mint
Drop	Land	soft-immature	Björkelunda	2022	Mint
Duck	Land	soft-immature	Björkelunda	2022	Mint
Duck	Water	soft-immature	Nöbbelöv	2022	Mint
Walk	Land	soft-immature	Nöbbelöv	2022	Gypsywort
Drop	Water	soft-immature	Häckeberga	2022	Marsh woundwort
Duck	Land	soft-immature	Björkelunda	2022	Mint
Duck	Water	soft-immature	Björkelunda	2022	Mint
Duck	Water	soft-immature	Björkelunda	2022	Mint
Duck	Water	soft-immature	Björkelunda	2022	Mint
Duck	Land	soft-immature	Björkelunda	2022	Marsh woundwort
Drop	Land	soft-immature	Björkelunda	2022	Marsh woundwort
Walk	Land	soft-immature	Björkelunda	2022	Mint

Duck	Water	soft-immature	Björkelunda	2022	Mint
Duck	Water	soft-immature	Björkelunda	2022	Mint
Duck	Water	hard-immature	Björkelunda	2022	Mint
Duck	Water	soft-immature	Björkelunda	2022	Mint
Duck	Water	hard-immature	Björkelunda	2022	Mint
Duck	Land	soft-immature	Björkelunda	2022	Mint
Duck	Water	soft-immature	Björkelunda	2022	Mint
Walk	Land	soft-immature	Nöbbelöv	2022	Gypsywort
Walk	Land	hard-immature	Nöbbelöv	2022	Gypsywort
Duck	Land	soft-immature	Nöbbelöv	2022	Gypsywort
Drop	Water	soft-immature	Häckeberga	2022	Marsh woundwort
Drop	Land	hard-immature	Björkelunda	2022	Mint
Duck	Land	soft-immature	Nöbbelöv	2022	Gypsywort
Walk	Land	soft-immature	Nöbbelöv	2022	Mint
Drop	Land	soft-immature	Nöbbelöv	2022	Gypsywort
Duck	Land	soft-immature	Björkelunda	2022	Mint
Duck	Land	soft-immature	Björkelunda	2022	Mint
Duck	Land	soft-immature	Björkelunda	2022	Mint
Duck	Land	soft-immature	Björkelunda	2022	Mint
Drop	Water	soft-immature	Björkelunda	2022	Mint
Walk	Water	soft-immature	Björkelunda	2022	Mint
Duck	Water	soft-immature	Björkelunda	2022	Mint
Duck	Land	soft-immature	Björkelunda	2022	Mint
Duck	Water	soft-immature	Björkelunda	2022	Mint
Drop	Water	soft-immature	Björkelunda	2022	Mint
Drop	Water	soft-immature	Björkelunda	2022	Mint
Drop	Water	soft-immature	Björkelunda	2022	Mint
Drop	Water	soft-immature	Björkelunda	2022	Mint
Duck	Water	soft-immature	Häckeberga	2022	Mint
Drop	Water	soft-immature	Häckeberga	2022	Mint
Duck	Water	soft-immature	Häckeberga	2022	Mint
Drop	Water	soft-immature	Häckeberga	2022	Mint
Drop	Water	soft-immature	Häckeberga	2022	Mint
Duck	Water	soft-immature	Häckeberga	2022	Mint
Duck	Water	soft-immature	Häckeberga	2022	Mint
Duck	Water	soft-immature	Häckeberga	2022	Mint
Drop	Land	soft-immature	Nöbbelöv	2022	Mint
Walk	Water	soft-immature	Nöbbelöv	2022	Mint
Drop	Land	soft-immature	Nöbbelöv	2022	Gypsywort
Drop	Land	soft-immature	Nöbbelöv	2022	Mint
Drop	Land	soft-immature	Nöbbelöv	2022	Mint
Drop	Water	soft-immature	Nöbbelöv	2022	Mint
Duck	Land	soft-immature	Nöbbelöv	2022	Gypsywort
Drop	Land	soft-immature	Nöbbelöv	2022	Mint
Drop	Land	soft-immature	Nöbbelöv	2022	Mint
Duck	Land	soft-immature	Nöbbelöv	2022	Mint
Walk	Water	hard-mature	Björkelunda	2022	Mint
