# Supplementary material for: Predator avoidance behaviour in green tortoise beetles depends on environment and shield ontogeny, but varies over time and space
Source: Oecologia. 2026 Nov 11;208(12):169. doi: 10.1007/s00442-026-06005-1 (PMC13751549; doi:10.1007/s00442-026-06005-1)
Supplement: Supplementary file 2 [file 442_2026_6005_MOESM3_ESM.pdf]

Supplementary material for the scientific article: "Predator avoidance behaviour in green tortoise beetles depends on environment and shield ontogeny, but varies over time and space"

Authors: Erica Winslott*, Kristina Karlsson Green, Maja Tarka

*Corresponding author: erica.winslott@biol.lu.se

Table S1 Full model *output (**Anova**, test = "LR"), in Drop.or.Duck response **walk is set as duck**, with **main effects** output.*

Model: glm(Drop.or.Duck ~ Substrate + Ontogeny + Population + Year, Data, family = binomial)

	LR Chisq	Df	Pr(>Chisq)
Substrate	26.411392	1	0.0000003
Ontogeny	63.569040	2	0.0000000
Population	10.788313	2	0.0045431
Year	1.063152	1	0.3024975

Table S2 Full model *output (**Anova**, test = "LR"), in Drop.or.Duck response **walk is removed**, with **main effects** output.*

Model: glm(Drop.or.Duck ~ Substrate + Ontogeny + Population + Year, Data, family = binomial)

	LR Chisq	Df	Pr(>Chisq)
Substrate	28.665930	1	0.0000001
Ontogeny	62.824209	2	0.0000000
Population	7.127082	2	0.0283383
Year	1.899329	1	0.1681534

Table S3 Full model *output (**Anova**, test = “LR”), in Drop.or.Duck response **walk is set as duck**, with **main effects and interactions** output.*

*Model: glm(Drop.or.Duck ~ Substrate * Ontogeny * Population * Year, Data, family = binomial)*

	**LR Chisq**	**Df**	**Pr(>Chisq)**
Substrate	22.8693297	1	0.0000017
Ontogeny	65.8310669	2	0.0000000
Population	7.4203307	2	0.0244735
Year	1.9528139	1	0.1622839
Substrate:Ontogeny	5.5588348	2	0.0620747
Substrate:Population	0.2118642	2	0.8994857
Ontogeny:Population	8.6754242	4	0.0697446
Substrate:Year	0.4652694	1	0.4951715
Ontogeny:Year	1.0617483	2	0.5880907
Population:Year	6.7118920	2	0.0348764
Substrate:Ontogeny:Population	1.0697988	4	0.8990339
Substrate:Ontogeny:Year	1.0490577	2	0.5918342
Substrate:Population:Year	4.9245921	2	0.0852390
Ontogeny:Population:Year	12.9413560	4	0.0115659
Substrate:Ontogeny:Population:Year	3.8722861	4	0.4235659

***Table S4 Full model** output (**Anova**, test = "LR"), in Drop.or.Duck response **walk is removed**, with **main effects and interactions** output.*

*Model: glm(Drop.or.Duck ~ Substrate * Ontogeny * Population * Year, Data, family = binomial)*

	**LR Chisq**	**Df**	**Pr(>Chisq)**
Substrate	25.7694905	1	0.0000004
Ontogeny	63.8365204	2	0.0000000
Population	4.9626390	2	0.0836328
Year	3.1456972	1	0.0761275
Substrate:Ontogeny	2.6425977	2	0.2667886
Substrate:Population	0.5700230	2	0.7520056
Ontogeny:Population	6.2350525	4	0.1822686
Substrate:Year	0.0307495	1	0.8608005
Ontogeny:Year	0.7326880	2	0.6932643
Population:Year	3.0213877	2	0.2207568
Substrate:Ontogeny:Population	0.9624829	4	0.9154304
Substrate:Ontogeny:Year	1.8814966	2	0.3903356
Substrate:Population:Year	5.8755335	2	0.0529839
Ontogeny:Population:Year	8.0704010	4	0.0890331
Substrate:Ontogeny:Population:Year	4.0562910	4	0.3984413

Table S5 *Output (**Anova**, test = "LR") from Model chosen with Backward elimination, the "**best fit model**", (glm(Drop.or.Duck~ Substrate + Ontogeny + Population + Year + Substrate * Ontogeny + Substrate * Population + Ontogeny * Population + Substrate * Year + Ontogeny * Year + Population * Year + Substrate * Population * Year + Ontogeny * Population * Year, Data, family = binomial)). For response variable Drop.or.Duck walk is set as duck.*

	LR Chisq	Df	Pr(>Chisq)
Substrate	22.8693297	1	0.0000017
Ontogeny	65.8310669	2	0.0000000
Population	7.5496524	2	0.0229411
Year	2.0579965	1	0.1514091
Substrate:Ontogeny	5.5588348	2	0.0620747
Substrate:Population	0.3009541	2	0.8602975
Ontogeny:Population	8.6692544	4	0.0699197
Substrate:Year	0.3416251	1	0.5588927
Ontogeny:Year	0.8630496	2	0.6495180
Population:Year	6.7242240	2	0.0346620
Substrate:Population:Year	5.2227000	2	0.0734353
Ontogeny:Population:Year	12.5796341	4	0.0135233

Table S6 *Output (**Anova**, test = "LR") from Model chosen with **Akaike Information Criterion** (AIC) (glm(Drop.or.Duck ~ Population + Shield + Substrate + Year + Population * Shield + Population * Year + Shield * Substrate + Shield * Year + Population * Shield *Year, Data, family = binomial)). For response variable Drop.or.Duck walk is set as duck.*

	LR Chisq	Df	Pr(>Chisq)
Population	8.7671205	2	0.0124808
Ontogeny	66.3574219	2	0.0000000
Substrate	22.8693297	1	0.0000017
Year	1.8457226	1	0.1742817
Population:Ontogeny	8.4458477	4	0.0765458
Population:Year	7.8883629	2	0.0193671
Ontogeny:Substrate	5.4293557	2	0.0662263
Ontogeny:Year	0.8780058	2	0.6446789
Population:Ontogeny:Year	12.3607277	4	0.0148610

Table S7 *From the* ***"best fit model"*** *-* ***Post hoc pairwise comparisons*** *of estimated marginal means for* ***Ontogeny × Population × Year****. Results are averaged over Substrate, expressed on the log-odds scale. P-values were adjusted for multiple comparisons using the Tukey method (family of 18 estimates).*

Table 7 (continued)

contrast	estimate	SE	df	z.ratio	p.value
(hard-immature Björkelunda Year2022) - (hard-mature Björkelunda Year2022)	-1.024	0.290	Inf	-3.525	0.044
(hard-immature Björkelunda Year2022) - (soft-immature Björkelunda Year2022)	0.057	0.250	Inf	0.229	1.000
(hard-immature Björkelunda Year2022) - (hard-immature Häckeberga Year2022)	0.223	0.333	Inf	0.670	1.000
(hard-immature Björkelunda Year2022) - (hard-mature Häckeberga Year2022)	-0.338	0.354	Inf	-0.955	1.000
(hard-immature Björkelunda Year2022) - (soft-immature Häckeberga Year2022)	0.308	0.274	Inf	1.123	1.000
(hard-immature Björkelunda Year2022) - (hard-immature Nöbbelöv Year2022)	0.385	0.407	Inf	0.947	1.000
(hard-immature Björkelunda Year2022) - (hard-mature Nöbbelöv Year2022)	-1.553	0.523	Inf	-2.968	0.216
(hard-immature Björkelunda Year2022) - (soft-immature Nöbbelöv Year2022)	0.333	0.346	Inf	0.962	1.000
(hard-immature Björkelunda Year2022) - (hard-immature Björkelunda Year2023)	-0.031	0.348	Inf	-0.089	1.000
(hard-immature Björkelunda Year2022) - (hard-mature Björkelunda Year2023)	-0.230	0.343	Inf	-0.669	1.000
(hard-immature Björkelunda Year2022) - (soft-immature Björkelunda Year2023)	-0.405	0.272	Inf	-1.486	0.992
(hard-immature Björkelunda Year2022) - (hard-immature Häckeberga Year2023)	0.695	0.607	Inf	1.145	1.000
(hard-immature Björkelunda Year2022) - (hard-mature Häckeberga Year2023)	-0.812	0.309	Inf	-2.629	0.432
(hard-immature Björkelunda Year2022) - (soft-immature Häckeberga Year2023)	0.562	0.311	Inf	1.809	0.942
(hard-immature Björkelunda Year2022) - (hard-immature Nöbbelöv Year2023)	0.203	0.812	Inf	0.250	1.000
(hard-immature Björkelunda Year2022) - (hard-mature Nöbbelöv Year2023)	-1.002	0.567	Inf	-1.768	0.953
(hard-immature Björkelunda Year2022) - (soft-immature Nöbbelöv Year2023)	1.296	0.550	Inf	2.358	0.640
(hard-mature Björkelunda Year2022) - (soft-immature Björkelunda Year2022)	1.081	0.279	Inf	3.879	0.013
(hard-mature Björkelunda Year2022) - (hard-immature Häckeberga Year2022)	1.247	0.362	Inf	3.446	0.057
(hard-mature Björkelunda Year2022) - (hard-mature Häckeberga Year2022)	0.685	0.367	Inf	1.865	0.925
(hard-mature Björkelunda Year2022) - (soft-immature Häckeberga Year2022)	1.331	0.297	Inf	4.484	0.001
(hard-mature Björkelunda Year2022) - (hard-immature Nöbbelöv Year2022)	1.409	0.416	Inf	3.388	0.069
(hard-mature Björkelunda Year2022) - (hard-mature Nöbbelöv Year2022)	-0.530	0.542	Inf	-0.977	1.000
(hard-mature Björkelunda Year2022) - (soft-immature Nöbbelöv Year2022)	1.357	0.369	Inf	3.672	0.027
(hard-mature Björkelunda Year2022) - (hard-immature Björkelunda Year2023)	0.993	0.380	Inf	2.614	0.443
(hard-mature Björkelunda Year2022) - (hard-mature Björkelunda Year2023)	0.794	0.361	Inf	2.202	0.753
(hard-mature Björkelunda Year2022) - (soft-immature Björkelunda Year2023)	0.619	0.298	Inf	2.077	0.830
(hard-mature Björkelunda Year2022) - (hard-immature Häckeberga Year2023)	1.719	0.627	Inf	2.743	0.350
(hard-mature Björkelunda Year2022) - (hard-mature Häckeberga Year2023)	0.211	0.331	Inf	0.637	1.000
(hard-mature Björkelunda Year2022) - (soft-immature Häckeberga Year2023)	1.586	0.334	Inf	4.742	0.000
(hard-mature Björkelunda Year2022) - (hard-immature Nöbbelöv Year2023)	1.227	0.816	Inf	1.504	0.991
(hard-mature Björkelunda Year2022) - (hard-mature Nöbbelöv Year2023)	0.022	0.582	Inf	0.037	1.000
(hard-mature Björkelunda Year2022) - (soft-immature Nöbbelöv Year2023)	2.320	0.563	Inf	4.121	0.005
(soft-immature Björkelunda Year2022) - (hard-immature Häckeberga Year2022)	0.166	0.335	Inf	0.496	1.000
(soft-immature Björkelunda Year2022) - (hard-mature Häckeberga Year2022)	-0.396	0.345	Inf	-1.146	1.000
(soft-immature Björkelunda Year2022) - (soft-immature Häckeberga Year2022)	0.251	0.252	Inf	0.995	1.000
(soft-immature Björkelunda Year2022) - (hard-immature Nöbbelöv Year2022)	0.328	0.385	Inf	0.852	1.000
(soft-immature Björkelunda Year2022) - (hard-mature Nöbbelöv Year2022)	-1.611	0.517	Inf	-3.113	0.150
(soft-immature Björkelunda Year2022) - (soft-immature Nöbbelöv Year2022)	0.276	0.345	Inf	0.801	1.000

Table 7 (continued)

contrast	estimate	SE	df	z.ratio	p.value
(soft-immature Björkelunda Year2022) - (hard-immature Björkelunda Year2023)	-0.088	0.355	Inf	-0.248	1.000
(soft-immature Björkelunda Year2022) - (hard-mature Björkelunda Year2023)	-0.287	0.335	Inf	-0.856	1.000
(soft-immature Björkelunda Year2022) - (soft-immature Björkelunda Year2023)	-0.462	0.253	Inf	-1.825	0.937
(soft-immature Björkelunda Year2022) - (hard-immature Häckeberga Year2023)	0.638	0.611	Inf	1.044	1.000
(soft-immature Björkelunda Year2022) - (hard-mature Häckeberga Year2023)	-0.870	0.300	Inf	-2.903	0.251
(soft-immature Björkelunda Year2022) - (soft-immature Häckeberga Year2023)	0.505	0.299	Inf	1.690	0.969
(soft-immature Björkelunda Year2022) - (hard-immature Nöbbelöv Year2023)	0.146	0.801	Inf	0.182	1.000
(soft-immature Björkelunda Year2022) - (hard-mature Nöbbelöv Year2023)	-1.059	0.561	Inf	-1.890	0.916
(soft-immature Björkelunda Year2022) - (soft-immature Nöbbelöv Year2023)	1.239	0.544	Inf	2.275	0.702
(hard-immature Häckeberga Year2022) - (hard-mature Häckeberga Year2022)	-0.561	0.386	Inf	-1.456	0.993
(hard-immature Häckeberga Year2022) - (soft-immature Häckeberga Year2022)	0.085	0.314	Inf	0.270	1.000
(hard-immature Häckeberga Year2022) - (hard-immature Nöbbelöv Year2022)	0.162	0.467	Inf	0.347	1.000
(hard-immature Häckeberga Year2022) - (hard-mature Nöbbelöv Year2022)	-1.776	0.564	Inf	-3.150	0.136
(hard-immature Häckeberga Year2022) - (soft-immature Nöbbelöv Year2022)	0.110	0.402	Inf	0.273	1.000
(hard-immature Häckeberga Year2022) - (hard-immature Björkelunda Year2023)	-0.254	0.398	Inf	-0.638	1.000
(hard-immature Häckeberga Year2022) - (hard-mature Björkelunda Year2023)	-0.453	0.404	Inf	-1.120	1.000
(hard-immature Häckeberga Year2022) - (soft-immature Björkelunda Year2023)	-0.628	0.349	Inf	-1.800	0.944
(hard-immature Häckeberga Year2022) - (hard-immature Häckeberga Year2023)	0.472	0.636	Inf	0.741	1.000
(hard-immature Häckeberga Year2022) - (hard-mature Häckeberga Year2023)	-1.036	0.375	Inf	-2.761	0.338
(hard-immature Häckeberga Year2022) - (soft-immature Häckeberga Year2023)	0.339	0.377	Inf	0.899	1.000
(hard-immature Häckeberga Year2022) - (hard-immature Nöbbelöv Year2023)	-0.020	0.844	Inf	-0.024	1.000
(hard-immature Häckeberga Year2022) - (hard-mature Nöbbelöv Year2023)	-1.225	0.606	Inf	-2.023	0.858
(hard-immature Häckeberga Year2022) - (soft-immature Nöbbelöv Year2023)	1.073	0.589	Inf	1.821	0.938
(hard-mature Häckeberga Year2022) - (soft-immature Häckeberga Year2022)	0.646	0.312	Inf	2.072	0.833
(hard-mature Häckeberga Year2022) - (hard-immature Nöbbelöv Year2022)	0.724	0.455	Inf	1.590	0.983
(hard-mature Häckeberga Year2022) - (hard-mature Nöbbelöv Year2022)	-1.215	0.588	Inf	-2.066	0.836
(hard-mature Häckeberga Year2022) - (soft-immature Nöbbelöv Year2022)	0.671	0.414	Inf	1.622	0.979
(hard-mature Häckeberga Year2022) - (hard-immature Björkelunda Year2023)	0.308	0.434	Inf	0.709	1.000
(hard-mature Häckeberga Year2022) - (hard-mature Björkelunda Year2023)	0.109	0.399	Inf	0.273	1.000
(hard-mature Häckeberga Year2022) - (soft-immature Björkelunda Year2023)	-0.067	0.364	Inf	-0.183	1.000
(hard-mature Häckeberga Year2022) - (hard-immature Häckeberga Year2023)	1.033	0.663	Inf	1.558	0.986
(hard-mature Häckeberga Year2022) - (hard-mature Häckeberga Year2023)	-0.474	0.381	Inf	-1.243	0.999
(hard-mature Häckeberga Year2022) - (soft-immature Häckeberga Year2023)	0.900	0.391	Inf	2.301	0.683
(hard-mature Häckeberga Year2022) - (hard-immature Nöbbelöv Year2023)	0.541	0.834	Inf	0.649	1.000
(hard-mature Häckeberga Year2022) - (hard-mature Nöbbelöv Year2023)	-0.664	0.620	Inf	-1.071	1.000
(hard-mature Häckeberga Year2022) - (soft-immature Nöbbelöv Year2023)	1.634	0.595	Inf	2.749	0.346
(soft-immature Häckeberga Year2022) - (hard-immature Nöbbelöv Year2022)	0.078	0.393	Inf	0.198	1.000
(soft-immature Häckeberga Year2022) - (hard-mature Nöbbelöv Year2022)	-1.861	0.525	Inf	-3.546	0.041
(soft-immature Häckeberga Year2022) - (soft-immature Nöbbelöv Year2022)	0.025	0.361	Inf	0.070	1.000
(soft-immature Häckeberga Year2022) - (hard-immature Björkelunda Year2023)	-0.339	0.372	Inf	-0.909	1.000
(soft-immature Häckeberga Year2022) - (hard-mature Björkelunda Year2023)	-0.537	0.351	Inf	-1.533	0.988
(soft-immature Häckeberga Year2022) - (soft-immature Björkelunda Year2023)	-0.713	0.267	Inf	-2.670	0.402
(soft-immature Häckeberga Year2022) - (hard-immature Häckeberga Year2023)	0.387	0.621	Inf	0.624	1.000
(soft-immature Häckeberga Year2022) - (hard-mature Häckeberga Year2023)	-1.120	0.315	Inf	-3.553	0.041
(soft-immature Häckeberga Year2022) - (soft-immature Häckeberga Year2023)	0.254	0.313	Inf	0.813	1.000
(soft-immature Häckeberga Year2022) - (hard-immature Nöbbelöv Year2023)	-0.105	0.805	Inf	-0.130	1.000

Table 7 (continued)

contrast	estimate	SE	df	z.ratio	p.value
(soft-immature Häckeberga Year2022) - (hard-mature Nöbbelöv Year2023)	-1.310	0.568	Inf	-2.308	0.678
(soft-immature Häckeberga Year2022) - (soft-immature Nöbbelöv Year2023)	0.988	0.553	Inf	1.786	0.948
(hard-immature Nöbbelöv Year2022) - (hard-mature Nöbbelöv Year2022)	-1.939	0.548	Inf	-3.539	0.042
(hard-immature Nöbbelöv Year2022) - (soft-immature Nöbbelöv Year2022)	-0.052	0.369	Inf	-0.142	1.000
(hard-immature Nöbbelöv Year2022) - (hard-immature Björkelunda Year2023)	-0.416	0.488	Inf	-0.852	1.000
(hard-immature Nöbbelöv Year2022) - (hard-mature Björkelunda Year2023)	-0.615	0.452	Inf	-1.360	0.997
(hard-immature Nöbbelöv Year2022) - (soft-immature Björkelunda Year2023)	-0.790	0.397	Inf	-1.992	0.874
(hard-immature Nöbbelöv Year2022) - (hard-immature Häckeberga Year2023)	0.309	0.698	Inf	0.443	1.000
(hard-immature Nöbbelöv Year2022) - (hard-mature Häckeberga Year2023)	-1.198	0.430	Inf	-2.786	0.321
(hard-immature Nöbbelöv Year2022) - (soft-immature Häckeberga Year2023)	0.176	0.430	Inf	0.410	1.000
(hard-immature Nöbbelöv Year2022) - (hard-immature Nöbbelöv Year2023)	-0.183	0.848	Inf	-0.215	1.000
(hard-immature Nöbbelöv Year2022) - (hard-mature Nöbbelöv Year2023)	-1.388	0.642	Inf	-2.161	0.780
(hard-immature Nöbbelöv Year2022) - (soft-immature Nöbbelöv Year2023)	0.910	0.626	Inf	1.454	0.993
(hard-mature Nöbbelöv Year2022) - (soft-immature Nöbbelöv Year2022)	1.886	0.515	Inf	3.660	0.028
(hard-mature Nöbbelöv Year2022) - (hard-immature Björkelunda Year2023)	1.523	0.572	Inf	2.660	0.409
(hard-mature Nöbbelöv Year2022) - (hard-mature Björkelunda Year2023)	1.324	0.577	Inf	2.294	0.688
(hard-mature Nöbbelöv Year2022) - (soft-immature Björkelunda Year2023)	1.148	0.523	Inf	2.195	0.758
(hard-mature Nöbbelöv Year2022) - (hard-immature Häckeberga Year2023)	2.248	0.756	Inf	2.974	0.213
(hard-mature Nöbbelöv Year2022) - (hard-mature Häckeberga Year2023)	0.741	0.552	Inf	1.343	0.997
(hard-mature Nöbbelöv Year2022) - (soft-immature Häckeberga Year2023)	2.115	0.547	Inf	3.865	0.013
(hard-mature Nöbbelöv Year2022) - (hard-immature Nöbbelöv Year2023)	1.756	0.931	Inf	1.886	0.918
(hard-mature Nöbbelöv Year2022) - (hard-mature Nöbbelöv Year2023)	0.551	0.721	Inf	0.764	1.000
(hard-mature Nöbbelöv Year2022) - (soft-immature Nöbbelöv Year2023)	2.849	0.714	Inf	3.990	0.008
(soft-immature Nöbbelöv Year2022) - (hard-immature Björkelunda Year2023)	-0.364	0.415	Inf	-0.877	1.000
(soft-immature Nöbbelöv Year2022) - (hard-mature Björkelunda Year2023)	-0.563	0.408	Inf	-1.381	0.996
(soft-immature Nöbbelöv Year2022) - (soft-immature Björkelunda Year2023)	-0.738	0.360	Inf	-2.049	0.845
(soft-immature Nöbbelöv Year2022) - (hard-immature Häckeberga Year2023)	0.362	0.648	Inf	0.558	1.000
(soft-immature Nöbbelöv Year2022) - (hard-mature Häckeberga Year2023)	-1.146	0.383	Inf	-2.993	0.203
(soft-immature Nöbbelöv Year2022) - (soft-immature Häckeberga Year2023)	0.229	0.387	Inf	0.591	1.000
(soft-immature Nöbbelöv Year2022) - (hard-immature Nöbbelöv Year2023)	-0.130	0.844	Inf	-0.154	1.000
(soft-immature Nöbbelöv Year2022) - (hard-mature Nöbbelöv Year2023)	-1.335	0.614	Inf	-2.175	0.771
(soft-immature Nöbbelöv Year2022) - (soft-immature Nöbbelöv Year2023)	0.963	0.594	Inf	1.620	0.979
(hard-immature Björkelunda Year2023) - (hard-mature Björkelunda Year2023)	-0.199	0.396	Inf	-0.503	1.000
(hard-immature Björkelunda Year2023) - (soft-immature Björkelunda Year2023)	-0.374	0.322	Inf	-1.161	1.000
(hard-immature Björkelunda Year2023) - (hard-immature Häckeberga Year2023)	0.726	0.639	Inf	1.135	1.000
(hard-immature Björkelunda Year2023) - (hard-mature Häckeberga Year2023)	-0.782	0.392	Inf	-1.994	0.873
(hard-immature Björkelunda Year2023) - (soft-immature Häckeberga Year2023)	0.593	0.393	Inf	1.507	0.990
(hard-immature Björkelunda Year2023) - (hard-immature Nöbbelöv Year2023)	0.234	0.857	Inf	0.273	1.000
(hard-immature Björkelunda Year2023) - (hard-mature Nöbbelöv Year2023)	-0.971	0.615	Inf	-1.580	0.984
(hard-immature Björkelunda Year2023) - (soft-immature Nöbbelöv Year2023)	1.327	0.600	Inf	2.213	0.745
(hard-mature Björkelunda Year2023) - (soft-immature Björkelunda Year2023)	-0.175	0.321	Inf	-0.545	1.000
(hard-mature Björkelunda Year2023) - (hard-immature Häckeberga Year2023)	0.925	0.654	Inf	1.414	0.995
(hard-mature Björkelunda Year2023) - (hard-mature Häckeberga Year2023)	-0.583	0.375	Inf	-1.555	0.986
(hard-mature Björkelunda Year2023) - (soft-immature Häckeberga Year2023)	0.792	0.382	Inf	2.075	0.831
(hard-mature Björkelunda Year2023) - (hard-immature Nöbbelöv Year2023)	0.433	0.834	Inf	0.519	1.000
(hard-mature Björkelunda Year2023) - (hard-mature Nöbbelöv Year2023)	-0.772	0.612	Inf	-1.262	0.999

Table 7 (continued)

contrast	estimate	SE	df	z.ratio	p.value
(hard-mature Björkelunda Year2023) - (soft-immature Nöbbelöv Year2023)	1.526	0.590	Inf	2.586	0.464
(soft-immature Björkelunda Year2023) - (hard-immature Häckeberga Year2023)	1.100	0.618	Inf	1.780	0.950
(soft-immature Björkelunda Year2023) - (hard-mature Häckeberga Year2023)	-0.408	0.316	Inf	-1.290	0.998
(soft-immature Björkelunda Year2023) - (soft-immature Häckeberga Year2023)	0.967	0.313	Inf	3.092	0.159
(soft-immature Björkelunda Year2023) - (hard-immature Nöbbelöv Year2023)	0.608	0.807	Inf	0.753	1.000
(soft-immature Björkelunda Year2023) - (hard-mature Nöbbelöv Year2023)	-0.597	0.567	Inf	-1.053	1.000
(soft-immature Björkelunda Year2023) - (soft-immature Nöbbelöv Year2023)	1.701	0.553	Inf	3.073	0.167
(hard-immature Häckeberga Year2023) - (hard-mature Häckeberga Year2023)	-1.507	0.627	Inf	-2.403	0.606
(hard-immature Häckeberga Year2023) - (soft-immature Häckeberga Year2023)	-0.133	0.625	Inf	-0.213	1.000
(hard-immature Häckeberga Year2023) - (hard-immature Nöbbelöv Year2023)	-0.492	0.992	Inf	-0.496	1.000
(hard-immature Häckeberga Year2023) - (hard-mature Nöbbelöv Year2023)	-1.697	0.790	Inf	-2.149	0.787
(hard-immature Häckeberga Year2023) - (soft-immature Nöbbelöv Year2023)	0.601	0.779	Inf	0.771	1.000
(hard-mature Häckeberga Year2023) - (soft-immature Häckeberga Year2023)	1.374	0.346	Inf	3.972	0.009
(hard-mature Häckeberga Year2023) - (hard-immature Nöbbelöv Year2023)	1.015	0.823	Inf	1.234	0.999
(hard-mature Häckeberga Year2023) - (hard-mature Nöbbelöv Year2023)	-0.190	0.591	Inf	-0.321	1.000
(hard-mature Häckeberga Year2023) - (soft-immature Nöbbelöv Year2023)	2.108	0.572	Inf	3.685	0.026
(soft-immature Häckeberga Year2023) - (hard-immature Nöbbelöv Year2023)	-0.359	0.824	Inf	-0.436	1.000
(soft-immature Häckeberga Year2023) - (hard-mature Nöbbelöv Year2023)	-1.564	0.589	Inf	-2.656	0.412
(soft-immature Häckeberga Year2023) - (soft-immature Nöbbelöv Year2023)	0.734	0.574	Inf	1.279	0.999
(hard-immature Nöbbelöv Year2023) - (hard-mature Nöbbelöv Year2023)	-1.205	0.898	Inf	-1.343	0.997
(hard-immature Nöbbelöv Year2023) - (soft-immature Nöbbelöv Year2023)	1.093	0.924	Inf	1.183	0.999
(hard-mature Nöbbelöv Year2023) - (soft-immature Nöbbelöv Year2023)	2.298	0.731	Inf	3.144	0.138

Table S8 *From the **“best fit model”** - **Estimated marginal means** (predicted probabilities) of ducking for **Ontogeny × Population × Year,** averaged over Substrate. Values are shown with 95% confidence intervals (asymp.LCL and asymp.UCL), back-transformed from the logit scale.*

Ontogeny	Population	Year	prob	SE	df	asymp.LCL	asymp.UCL
hard-immature	Björkelunda	2022	0.5140	0.0467	Inf	0.4230	0.6040
hard-mature	Björkelunda	2022	0.7464	0.0423	Inf	0.6552	0.8201
soft-immature	Björkelunda	2022	0.4997	0.0423	Inf	0.4175	0.5819
hard-immature	Häckeberga	2022	0.4583	0.0703	Inf	0.3269	0.5958
hard-mature	Häckeberga	2022	0.5973	0.0714	Inf	0.4533	0.7263
soft-immature	Häckeberga	2022	0.4374	0.0479	Inf	0.3468	0.5324
hard-immature	Nöbbelöv	2022	0.4184	0.0858	Inf	0.2649	0.5895
hard-mature	Nöbbelöv	2022	0.8333	0.0682	Inf	0.6563	0.9290
soft-immature	Nöbbelöv	2022	0.4312	0.0724	Inf	0.2983	0.5748
hard-immature	Björkelunda	2023	0.5217	0.0760	Inf	0.3751	0.6647
hard-mature	Björkelunda	2023	0.5709	0.0701	Inf	0.4316	0.6998
soft-immature	Björkelunda	2023	0.6132	0.0463	Inf	0.5196	0.6992
hard-immature	Häckeberga	2023	0.3455	0.1320	Inf	0.1440	0.6236
hard-mature	Häckeberga	2023	0.7044	0.0512	Inf	0.5955	0.7942
soft-immature	Häckeberga	2023	0.3762	0.0582	Inf	0.2705	0.4950
hard-immature	Nöbbelöv	2023	0.4633	0.1954	Inf	0.1562	0.8011
hard-mature	Nöbbelöv	2023	0.7423	0.1025	Inf	0.5020	0.8917
soft-immature	Nöbbelöv	2023	0.2245	0.0900	Inf	0.0951	0.4436

Table S9 *From the **"best fit model"** - **Post hoc pairwise comparisons** of estimated marginal means for **Substrate × Population × Year.** Results are averaged over Ontogeny, expressed on the log-odds scale. P-values were adjusted for multiple comparisons using the Tukey method (family of 12 estimates).*

**Table 9 (continued)**					
**contrast**	**estimate**	**SE**	**df**	**z.ratio**	**p.value**
Land Björkelunda Year2022 - Water Björkelunda Year2022	-0.837	0.224	Inf	-3.738	0.010
Land Björkelunda Year2022 - Land Häckeberga Year2022	0.454	0.359	Inf	1.265	0.983
Land Björkelunda Year2022 - Water Häckeberga Year2022	-0.518	0.225	Inf	-2.301	0.476
Land Björkelunda Year2022 - Land Nöbbelöv Year2022	-0.369	0.267	Inf	-1.383	0.967
Land Björkelunda Year2022 - Water Nöbbelöv Year2022	-0.381	0.518	Inf	-0.735	1.000
Land Björkelunda Year2022 - Land Björkelunda Year2023	0.010	0.358	Inf	0.028	1.000
Land Björkelunda Year2022 - Water Björkelunda Year2023	-0.646	0.229	Inf	-2.823	0.170
Land Björkelunda Year2022 - Land Häckeberga Year2023	0.191	0.380	Inf	0.503	1.000
Land Björkelunda Year2022 - Water Häckeberga Year2023	-0.088	0.301	Inf	-0.292	1.000
Land Björkelunda Year2022 - Land Nöbbelöv Year2023	0.862	0.430	Inf	2.005	0.690
Land Björkelunda Year2022 - Water Nöbbelöv Year2023	-0.724	0.665	Inf	-1.088	0.995
Water Björkelunda Year2022 - Land Häckeberga Year2022	1.291	0.344	Inf	3.747	0.010
Water Björkelunda Year2022 - Water Häckeberga Year2022	0.319	0.194	Inf	1.641	0.894
Water Björkelunda Year2022 - Land Nöbbelöv Year2022	0.468	0.243	Inf	1.926	0.743
Water Björkelunda Year2022 - Water Nöbbelöv Year2022	0.456	0.503	Inf	0.907	0.999
Water Björkelunda Year2022 - Land Björkelunda Year2023	0.847	0.341	Inf	2.484	0.350
Water Björkelunda Year2022 - Water Björkelunda Year2023	0.191	0.198	Inf	0.965	0.998
Water Björkelunda Year2022 - Land Häckeberga Year2023	1.028	0.361	Inf	2.846	0.161
Water Björkelunda Year2022 - Water Häckeberga Year2023	0.749	0.280	Inf	2.680	0.236
Water Björkelunda Year2022 - Land Nöbbelöv Year2023	1.699	0.415	Inf	4.098	0.002
Water Björkelunda Year2022 - Water Nöbbelöv Year2023	0.113	0.656	Inf	0.172	1.000
Land Häckeberga Year2022 - Water Häckeberga Year2022	-0.972	0.334	Inf	-2.906	0.138
Land Häckeberga Year2022 - Land Nöbbelöv Year2022	-0.822	0.370	Inf	-2.225	0.531
Land Häckeberga Year2022 - Water Nöbbelöv Year2022	-0.834	0.582	Inf	-1.434	0.957
Land Häckeberga Year2022 - Land Björkelunda Year2023	-0.444	0.438	Inf	-1.015	0.997
Land Häckeberga Year2022 - Water Björkelunda Year2023	-1.100	0.346	Inf	-3.179	0.065
Land Häckeberga Year2022 - Land Häckeberga Year2023	-0.263	0.459	Inf	-0.572	1.000
Land Häckeberga Year2022 - Water Häckeberga Year2023	-0.542	0.396	Inf	-1.367	0.970
Land Häckeberga Year2022 - Land Nöbbelöv Year2023	0.409	0.502	Inf	0.815	1.000
Land Häckeberga Year2022 - Water Nöbbelöv Year2023	-1.178	0.715	Inf	-1.647	0.891
Water Häckeberga Year2022 - Land Nöbbelöv Year2022	0.150	0.242	Inf	0.618	1.000
Water Häckeberga Year2022 - Water Nöbbelöv Year2022	0.138	0.503	Inf	0.273	1.000
Water Häckeberga Year2022 - Land Björkelunda Year2023	0.528	0.342	Inf	1.544	0.928
Water Häckeberga Year2022 - Water Björkelunda Year2023	-0.128	0.197	Inf	-0.649	1.000
Water Häckeberga Year2022 - Land Häckeberga Year2023	0.710	0.365	Inf	1.946	0.730
Water Häckeberga Year2022 - Water Häckeberga Year2023	0.430	0.279	Inf	1.545	0.928
Water Häckeberga Year2022 - Land Nöbbelöv Year2023	1.381	0.415	Inf	3.330	0.041
Water Häckeberga Year2022 - Water Nöbbelöv Year2023	-0.206	0.654	Inf	-0.315	1.000
Land Nöbbelöv Year2022 - Water Nöbbelöv Year2022	-0.012	0.509	Inf	-0.024	1.000

Table 9 (continued)

contrast	estimate	SE	df	z.ratio	p.value
Land Nöbbelöv Year2022 - Land Björkelunda Year2023	0.378	0.369	Inf	1.026	0.997
Land Nöbbelöv Year2022 - Water Björkelunda Year2023	-0.278	0.246	Inf	-1.130	0.993
Land Nöbbelöv Year2022 - Land Häckeberga Year2023	0.560	0.391	Inf	1.432	0.957
Land Nöbbelöv Year2022 - Water Häckeberga Year2023	0.281	0.314	Inf	0.895	0.999
Land Nöbbelöv Year2022 - Land Nöbbelöv Year2023	1.231	0.439	Inf	2.803	0.178
Land Nöbbelöv Year2022 - Water Nöbbelöv Year2023	-0.355	0.671	Inf	-0.530	1.000
Water Nöbbelöv Year2022 - Land Björkelunda Year2023	0.391	0.580	Inf	0.673	1.000
Water Nöbbelöv Year2022 - Water Björkelunda Year2023	-0.266	0.505	Inf	-0.526	1.000
Water Nöbbelöv Year2022 - Land Häckeberga Year2023	0.572	0.592	Inf	0.967	0.998
Water Nöbbelöv Year2022 - Water Häckeberga Year2023	0.293	0.542	Inf	0.540	1.000
Water Nöbbelöv Year2022 - Land Nöbbelöv Year2023	1.243	0.623	Inf	1.995	0.697
Water Nöbbelöv Year2022 - Water Nöbbelöv Year2023	-0.343	0.802	Inf	-0.428	1.000
Land Björkelunda Year2023 - Water Björkelunda Year2023	-0.656	0.335	Inf	-1.961	0.720
Land Björkelunda Year2023 - Land Häckeberga Year2023	0.181	0.448	Inf	0.405	1.000
Land Björkelunda Year2023 - Water Häckeberga Year2023	-0.098	0.396	Inf	-0.247	1.000
Land Björkelunda Year2023 - Land Nöbbelöv Year2023	0.853	0.500	Inf	1.707	0.866
Land Björkelunda Year2023 - Water Nöbbelöv Year2023	-0.734	0.718	Inf	-1.022	0.997
Water Björkelunda Year2023 - Land Häckeberga Year2023	0.837	0.365	Inf	2.295	0.480
Water Björkelunda Year2023 - Water Häckeberga Year2023	0.558	0.282	Inf	1.983	0.705
Water Björkelunda Year2023 - Land Nöbbelöv Year2023	1.509	0.416	Inf	3.623	0.015
Water Björkelunda Year2023 - Water Nöbbelöv Year2023	-0.078	0.656	Inf	-0.119	1.000
Land Häckeberga Year2023 - Water Häckeberga Year2023	-0.279	0.358	Inf	-0.779	1.000
Land Häckeberga Year2023 - Land Nöbbelöv Year2023	0.671	0.515	Inf	1.304	0.979
Land Häckeberga Year2023 - Water Nöbbelöv Year2023	-0.915	0.732	Inf	-1.251	0.985
Water Häckeberga Year2023 - Land Nöbbelöv Year2023	0.950	0.461	Inf	2.063	0.650
Water Häckeberga Year2023 - Water Nöbbelöv Year2023	-0.636	0.684	Inf	-0.930	0.999
Land Nöbbelöv Year2023 - Water Nöbbelöv Year2023	-1.586	0.735	Inf	-2.158	0.581

***Table S10** From the "**best fit model**" - **Estimated marginal means** (predicted probabilities) of ducking for **Substrate × Population × Year,** averaged over Ontogeny. Values are shown with 95% confidence intervals (asymp.LCL and asymp.UCL), back-transformed from the logit scale.*

Substrate	Population	Year	prob	SE	df	asymp.LCL	asymp.UCL
Land	Björkelunda	2022	0.4899	0.0445	Inf	0.4039	0.5766
Water	Björkelunda	2022	0.6893	0.0296	Inf	0.6285	0.7442
Land	Häckeberga	2022	0.3789	0.0737	Inf	0.2483	0.5298
Water	Häckeberga	2022	0.6172	0.0324	Inf	0.5520	0.6785
Land	Nöbbelöv	2022	0.5813	0.0484	Inf	0.4845	0.6723
Water	Nöbbelöv	2022	0.5843	0.1179	Inf	0.3518	0.7844
Land	Björkelunda	2023	0.4875	0.0777	Inf	0.3409	0.6362
Water	Björkelunda	2023	0.6470	0.0326	Inf	0.5808	0.7080
Land	Häckeberga	2023	0.4423	0.0827	Inf	0.2913	0.6049
Water	Häckeberga	2023	0.5119	0.0607	Inf	0.3945	0.6279
Land	Nöbbelöv	2023	0.2885	0.0803	Inf	0.1585	0.4661
Water	Nöbbelöv	2023	0.6646	0.1429	Inf	0.3606	0.8744

***Table S11** From the "**best fit model**" - **Post hoc pairwise comparisons** of estimated marginal means for **Population × Year.** Results are averaged over Ontogeny and Substrate, expressed on the log-odds scale. P-values were adjusted for multiple comparisons using the Tukey method (family of 6 estimates).*

contrast	estimate	SE	df	z.ratio	p.value
Björkelunda Year2022 - Häckeberga Year2022	0.386	0.208	Inf	1.853	0.431
Björkelunda Year2022 - Nöbbelöv Year2022	0.044	0.293	Inf	0.150	1.000
Björkelunda Year2022 - Björkelunda Year2023	0.100	0.208	Inf	0.482	0.997
Björkelunda Year2022 - Häckeberga Year2023	0.470	0.258	Inf	1.825	0.449
Björkelunda Year2022 - Nöbbelöv Year2023	0.488	0.400	Inf	1.220	0.827
Häckeberga Year2022 - Nöbbelöv Year2022	-0.342	0.322	Inf	-1.062	0.896
Häckeberga Year2022 - Björkelunda Year2023	-0.286	0.247	Inf	-1.160	0.856
Häckeberga Year2022 - Häckeberga Year2023	0.084	0.291	Inf	0.289	1.000
Häckeberga Year2022 - Nöbbelöv Year2023	0.101	0.421	Inf	0.241	1.000
Nöbbelöv Year2022 - Björkelunda Year2023	0.056	0.323	Inf	0.175	1.000
Nöbbelöv Year2022 - Häckeberga Year2023	0.426	0.356	Inf	1.196	0.839
Nöbbelöv Year2022 - Nöbbelöv Year2023	0.444	0.468	Inf	0.949	0.934
Björkelunda Year2023 - Häckeberga Year2023	0.370	0.287	Inf	1.288	0.792
Björkelunda Year2023 - Nöbbelöv Year2023	0.387	0.423	Inf	0.915	0.943
Häckeberga Year2023 - Nöbbelöv Year2023	0.017	0.450	Inf	0.039	1.000

Table S12 *From the **“best fit model”** - **Estimated marginal means** (predicted probabilities) of ducking for **Population × Year,** averaged over Ontogeny and Substrate. Values are shown with 95% confidence intervals (asymp.LCL and asymp.UCL), back-transformed from the logit scale.*

**Population**	**Year**	**prob**	**SE**	**df**	**asymp.LCL**	**asymp.UCL**
Björkelunda	2022	0.5934	0.0274	Inf	0.5388	0.6458
Häckeberga	2022	0.4980	0.0436	Inf	0.4134	0.5827
Nöbbelöv	2022	0.5828	0.0656	Inf	0.4515	0.7034
Björkelunda	2023	0.5690	0.0428	Inf	0.4839	0.6503
Häckeberga	2023	0.4770	0.0578	Inf	0.3668	0.5895
Nöbbelöv	2023	0.4726	0.0955	Inf	0.2972	0.6551

***Table S13** From the **"best fit model"** - **Post hoc pairwise comparisons** of estimated marginal means for **Ontogeny × Population**. Results are averaged over Substrate and Year, expressed on the log-odds scale. P-values were adjusted for multiple comparisons using the Tukey method (family of 9 estimates).*

contrast	estimate	SE	df	z.ratio	p.value
(hard-immature Björkelunda) - (hard-mature Björkelunda)	-0.611	0.251	Inf	-2.435	0.265
(hard-immature Björkelunda) - (soft-immature Björkelunda)	-0.158	0.213	Inf	-0.744	0.998
(hard-immature Björkelunda) - (hard-immature Häckeberga)	0.474	0.360	Inf	1.316	0.927
(hard-immature Björkelunda) - (hard-mature Häckeberga)	-0.560	0.272	Inf	-2.058	0.503
(hard-immature Björkelunda) - (soft-immature Häckeberga)	0.450	0.248	Inf	1.814	0.673
(hard-immature Björkelunda) - (hard-immature Nöbbelöv)	0.310	0.489	Inf	0.633	0.999
(hard-immature Björkelunda) - (hard-mature Nöbbelöv)	-1.262	0.406	Inf	-3.108	0.049
(hard-immature Björkelunda) - (soft-immature Nöbbelöv)	0.830	0.346	Inf	2.398	0.285
(hard-mature Björkelunda) - (soft-immature Björkelunda)	0.453	0.217	Inf	2.089	0.481
(hard-mature Björkelunda) - (hard-immature Häckeberga)	1.086	0.381	Inf	2.852	0.101
(hard-mature Björkelunda) - (hard-mature Häckeberga)	0.051	0.259	Inf	0.198	1.000
(hard-mature Björkelunda) - (soft-immature Häckeberga)	1.062	0.245	Inf	4.327	0.001
(hard-mature Björkelunda) - (hard-immature Nöbbelöv)	0.921	0.471	Inf	1.954	0.575
(hard-mature Björkelunda) - (hard-mature Nöbbelöv)	-0.651	0.415	Inf	-1.568	0.822
(hard-mature Björkelunda) - (soft-immature Nöbbelöv)	1.441	0.348	Inf	4.143	0.001
(soft-immature Björkelunda) - (hard-immature Häckeberga)	0.633	0.360	Inf	1.756	0.711
(soft-immature Björkelunda) - (hard-mature Häckeberga)	-0.402	0.241	Inf	-1.669	0.766
(soft-immature Björkelunda) - (soft-immature Häckeberga)	0.609	0.200	Inf	3.045	0.059
(soft-immature Björkelunda) - (hard-immature Nöbbelöv)	0.468	0.452	Inf	1.036	0.982
(soft-immature Björkelunda) - (hard-mature Nöbbelöv)	-1.104	0.385	Inf	-2.867	0.097
(soft-immature Björkelunda) - (soft-immature Nöbbelöv)	0.988	0.329	Inf	3.001	0.067
(hard-immature Häckeberga) - (hard-mature Häckeberga)	-1.034	0.378	Inf	-2.735	0.136
(hard-immature Häckeberga) - (soft-immature Häckeberga)	-0.024	0.359	Inf	-0.067	1.000
(hard-immature Häckeberga) - (hard-immature Nöbbelöv)	-0.165	0.565	Inf	-0.292	1.000
(hard-immature Häckeberga) - (hard-mature Nöbbelöv)	-1.737	0.489	Inf	-3.555	0.011
(hard-immature Häckeberga) - (soft-immature Nöbbelöv)	0.355	0.441	Inf	0.806	0.997
(hard-mature Häckeberga) - (soft-immature Häckeberga)	1.010	0.238	Inf	4.248	0.001
(hard-mature Häckeberga) - (hard-immature Nöbbelöv)	0.870	0.474	Inf	1.834	0.660
(hard-mature Häckeberga) - (hard-mature Nöbbelöv)	-0.702	0.424	Inf	-1.658	0.772
(hard-mature Häckeberga) - (soft-immature Nöbbelöv)	1.390	0.353	Inf	3.932	0.003
(soft-immature Häckeberga) - (hard-immature Nöbbelöv)	-0.141	0.461	Inf	-0.305	1.000
(soft-immature Häckeberga) - (hard-mature Nöbbelöv)	-1.713	0.396	Inf	-4.329	0.001
(soft-immature Häckeberga) - (soft-immature Nöbbelöv)	0.380	0.341	Inf	1.114	0.972
(hard-immature Nöbbelöv) - (hard-mature Nöbbelöv)	-1.572	0.535	Inf	-2.937	0.080
(hard-immature Nöbbelöv) - (soft-immature Nöbbelöv)	0.520	0.505	Inf	1.030	0.983
(hard-mature Nöbbelöv) - (soft-immature Nöbbelöv)	2.092	0.451	Inf	4.640	0.000

Table S14 *From the* ***"best fit model"*** *-* ***Estimated marginal means*** *(predicted probabilities) of ducking for* ***Ontogeny × Population****, averaged over Substrate and Year. Values are shown with 95% confidence intervals (asymp.LCL and asymp.UCL), back-transformed from the logit scale.*

Ontogeny	Population	prob	SE	df	asymp.LCL	asymp.UCL
hard-immature	Björkelunda	0.5179	0.0458	Inf	0.4285	0.6061
hard-mature	Björkelunda	0.6643	0.0407	Inf	0.5805	0.7390
soft-immature	Björkelunda	0.5572	0.0325	Inf	0.4929	0.6196
hard-immature	Häckeberga	0.4006	0.0793	Inf	0.2592	0.5608
hard-mature	Häckeberga	0.6528	0.0441	Inf	0.5622	0.7336
soft-immature	Häckeberga	0.4064	0.0383	Inf	0.3340	0.4832
hard-immature	Nöbbelöv	0.4407	0.1077	Inf	0.2507	0.6498
hard-mature	Nöbbelöv	0.7915	0.0604	Inf	0.6493	0.8861
soft-immature	Nöbbelöv	0.3190	0.0647	Inf	0.2071	0.4565

Table S15 *From the* ***"best fit model"*** *-* ***Post hoc pairwise comparisons*** *of estimated marginal means for* ***Substrate × Ontogeny****. Results are averaged over Population and Year, expressed on the log-odds scale. P-values were adjusted for multiple comparisons using the Tukey method (family of 6 estimates).*

contrast	estimate	SE	df	z.ratio	p.value
(Land hard-immature) - (Water hard-immature)	-0.587	0.321	Inf	-1.828	0.448
(Land hard-immature) - (Land hard-mature)	-0.776	0.315	Inf	-2.460	0.136
(Land hard-immature) - (Water hard-mature)	-1.956	0.320	Inf	-6.103	0.000
(Land hard-immature) - (Land soft-immature)	0.022	0.291	Inf	0.075	1.000
(Land hard-immature) - (Water soft-immature)	-0.383	0.293	Inf	-1.308	0.781
(Water hard-immature) - (Land hard-mature)	-0.189	0.322	Inf	-0.588	0.992
(Water hard-immature) - (Water hard-mature)	-1.369	0.284	Inf	-4.816	0.000
(Water hard-immature) - (Land soft-immature)	0.608	0.309	Inf	1.970	0.360
(Water hard-immature) - (Water soft-immature)	0.203	0.256	Inf	0.795	0.968
(Land hard-mature) - (Water hard-mature)	-1.179	0.286	Inf	-4.124	0.001
(Land hard-mature) - (Land soft-immature)	0.798	0.258	Inf	3.088	0.025
(Land hard-mature) - (Water soft-immature)	0.393	0.260	Inf	1.514	0.655
(Water hard-mature) - (Land soft-immature)	1.977	0.276	Inf	7.171	0.000
(Water hard-mature) - (Water soft-immature)	1.572	0.230	Inf	6.822	0.000
(Land soft-immature) - (Water soft-immature)	-0.405	0.227	Inf	-1.782	0.478

Table S16 *From the* ***"best fit model"*** *-* ***Estimated marginal means*** *(predicted probabilities) of ducking for* ***Substrate × Ontogeny****, averaged over Population and Year. Values are shown with 95% confidence intervals (asymp.LCL and asymp.UCL), back-transformed from the logit scale.*

Substrate	Ontogeny	prob	SE	df	asymp.LCL	asymp.UCL
Land	hard-immature	0.3815	0.0579	Inf	0.2760	0.4995
Water	hard-immature	0.5258	0.0614	Inf	0.4063	0.6425
Land	hard-mature	0.5727	0.0492	Inf	0.4747	0.6653
Water	hard-mature	0.8134	0.0319	Inf	0.7427	0.8681
Land	soft-immature	0.3764	0.0395	Inf	0.3026	0.4563
Water	soft-immature	0.4750	0.0405	Inf	0.3970	0.5543

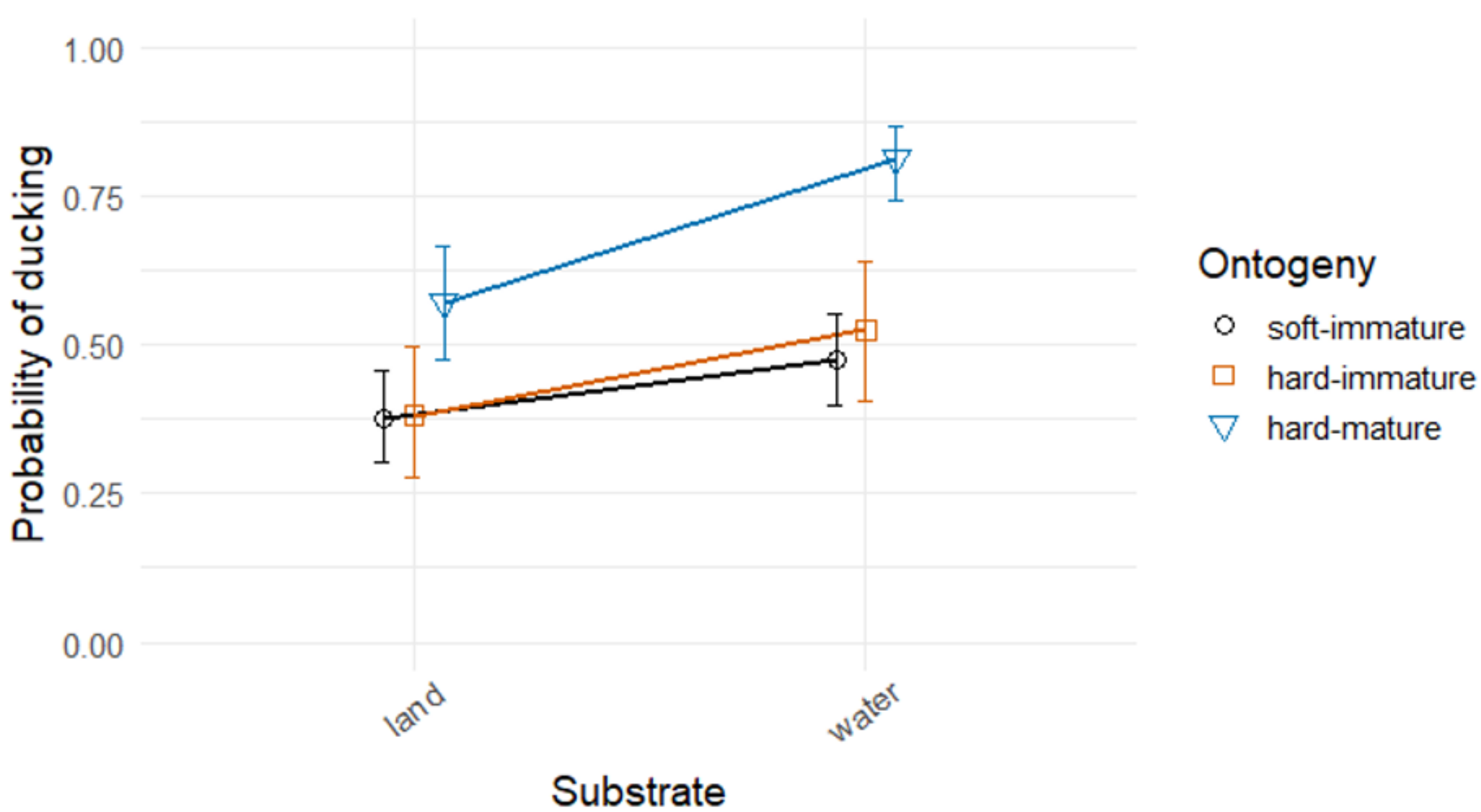

Figure S1 *Probability of ducking behaviour in Cassida viridis depending on ontogeny (black open circle = soft-immature, orange open square = hard-immature, and blue open triangle = hard-mature) and on the substrate beneath the host plant (land or water). Points show estimated marginal means from the* ***"best fit model"****; error bars represent 95% confidence intervals calculated using the emmeans package in R.*

Table S17 *Beetle frequency (5th column) separated by the three study populations (Björkelunda, Häckeberga, and Nöbbelöv), substrate (land and water), shield ontogeny (hard-mature, hard-immature, and soft-immature) and predator avoidance behaviour (drop and duck).*

Population	Substrate	Shield Ontogeny	Predator avoidance behaviour	n
Björkelunda	land	hard-immature	Drop	30
Björkelunda	water	hard-immature	Drop	55
Björkelunda	land	hard-mature	Drop	22
Björkelunda	water	hard-mature	Drop	29
Björkelunda	land	soft-immature	Drop	38
Björkelunda	water	soft-immature	Drop	113
Björkelunda	land	hard-immature	Duck	21
Björkelunda	water	hard-immature	Duck	83
Björkelunda	land	hard-mature	Duck	29
Björkelunda	water	hard-mature	Duck	110
Björkelunda	land	soft-immature	Duck	36
Björkelunda	water	soft-immature	Duck	184
Häckeberga	land	hard-immature	Drop	9
Häckeberga	water	hard-immature	Drop	30
Häckeberga	land	hard-mature	Drop	16
Häckeberga	water	hard-mature	Drop	31
Häckeberga	land	soft-immature	Drop	33
Häckeberga	water	soft-immature	Drop	127
Häckeberga	land	hard-immature	Duck	7
Häckeberga	water	hard-immature	Duck	30
Häckeberga	land	hard-mature	Duck	21
Häckeberga	water	hard-mature	Duck	101
Häckeberga	land	soft-immature	Duck	18
Häckeberga	water	soft-immature	Duck	125
Nöbbelöv	land	hard-immature	Drop	36
Nöbbelöv	water	hard-immature	Drop	3
Nöbbelöv	land	hard-mature	Drop	11
Nöbbelöv	water	hard-mature	Drop	2
Nöbbelöv	land	soft-immature	Drop	58
Nöbbelöv	water	soft-immature	Drop	11
Nöbbelöv	land	hard-immature	Duck	25
Nöbbelöv	water	hard-immature	Duck	3
Nöbbelöv	land	hard-mature	Duck	26
Nöbbelöv	water	hard-mature	Duck	15
Nöbbelöv	land	soft-immature	Duck	40
Nöbbelöv	water	soft-immature	Duck	6

Table S18 "Pooled host plant model" *output (**Anova**, test = "LR") with **main effects and their interactions.** For response variable Drop.or.Duck: walk is set as duck.*

*Model: (glm(Drop.orDuck ~ Substrate * Host plant * Ontogeny, Data, family = binomial.*

	LR Chisq	Df	Pr(>Chisq)
Substrate	35.0381018	1	0.0000000
Hostplant	5.5114613	2	0.0635626
Ontogeny	63.9220930	2	0.0000000
Substrate:Hostplant	3.1992363	2	0.2019736
Substrate:Ontogeny	0.9183881	2	0.6317926
Hostplant:Ontogeny	4.5570452	4	0.3358369
Substrate:Hostplant:Ontogeny	4.2477309	4	0.3735161

Table S19 "Pooled host plant model" *output (Anova, test = "LR") with **main effects**, without interactions since these were non-significant in the backward elimination process. For response variable Drop.or.Duck: walk is set as duck.*

Model: (glm(Drop.orDuck ~ Substrate + Host plant + Ontogeny, Data, family = binomial)

	LR Chisq	Df	Pr(>Chisq)
Substrate	34.107078	1	0.000000
Hostplant	5.480879	2	0.064542
Ontogeny	64.565487	2	0.000000

Table S20 *From the **"Pooled host plant model"** with main effects - **Post hoc pairwise comparisons** of estimated marginal means for **Host plant**. Results are averaged over Substrate and Ontogeny, expressed on the log-odds scale. P-values were adjusted for multiple comparisons using the Tukey method (family of 3 estimates).*

contrast	estimate	SE	df	z.ratio	p.value
Gypsywort - Marsh woundwort	0.5893	0.2632	Inf	2.239	0.0648
Gypsywort - Mint	0.2358	0.2032	Inf	1.160	0.4770
Marsh woundwort - Mint	-0.3536	0.1812	Inf	-1.952	0.1245

Table S21 *From the* ***"Pooled host plant model"*** *with main effects -* ***Estimated marginal means*** *(predicted probabilities) of ducking for* ***Host plant****, averaged over Substrate and Ontogeny. Values are shown with 95% confidence intervals (asymp.LCL and asymp.UCL), back-transformed from the logit scale.*

**Hostplant**	**prob**	**SE**	**df**	**asymp.LCL**	**asymp.UCL**
Gypsywort	0.6236	0.0444	Inf	0.5335	0.7058
Marsh woundwort	0.4789	0.0441	Inf	0.3939	0.5651
Mint	0.5669	0.0170	Inf	0.5333	0.5998

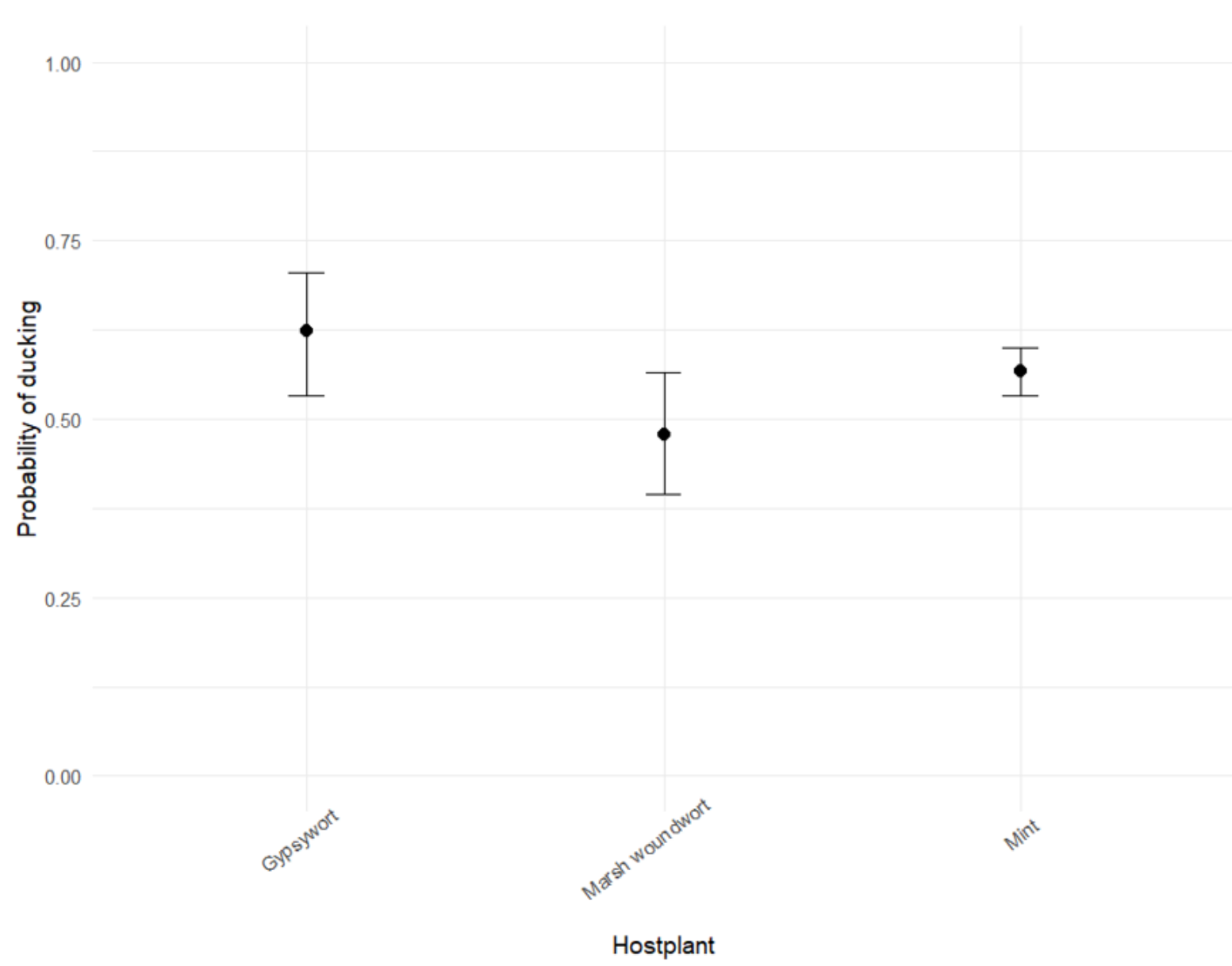

Figure S2 *Probability of ducking behaviour dependent on host plant species (gypsywort, mint, marsh woundwort). Points show estimated marginal means from the* ***"Pooled host plant model"*** *with main effects; error bars represent 95% confidence intervals calculated using the emmeans package in R.*

Table S22 *Overall* ***Chi-square*** *test results for* ***Host plant × Ontogeny, with pooled Population and Year data.*** *The table shows the Chi-squared statistics, degrees of freedom, and p-value testing independence between the factors.*

Test	Chi_Sq	DF	P_value
Hostplant × Ontogeny	1.123693	4	0.8904951

Table S23 Cell-level contributions for Host plant × Ontogeny, with pooled Population and Year data. *Observed and expected counts, as well as standardized residuals, are shown for each combination of factors. Standardized residuals indicate which cells contribute most to the overall Chi-squared statistics. P-value is reported in Table S23.*

Hostplant	Ontogeny	Observed	Expected	Std_Residual
Gypsywort	hard-immature	31	27.01937	0.9015900
Marsh woundwort	hard-immature	33	31.24115	0.3732127
Mint	hard-immature	252	257.73948	-0.9373986
Gypsywort	hard-mature	34	34.20174	-0.0421384
Marsh woundwort	hard-mature	38	39.54576	-0.3024852
Mint	hard-mature	328	326.25250	0.2632103
Gypsywort	soft-immature	63	66.77889	-0.6992111
Marsh woundwort	soft-immature	77	77.21309	-0.0369388
Mint	soft-immature	641	637.00802	0.5326318

Supplementary material for the scientific article: "Predator avoidance behaviour in green tortoise beetles depends on environment and shield ontogeny, but varies over time and space"

Methods and Results from Chi-square tests: Host plant × Ontogeny

Methods: Because sample sizes were insufficient for separate analyses, data were pooled across populations and years. We used Pearson's Chi-square test to determine whether the observed distribution of ontogenetic stages differed from that expected under the assumption of independence. Significance was assessed based on Chi-square statistics, p-values, and inspection of residual patterns.

Results: The results show an even distribution of ontogenetic stages across all host plants (Supplementary table S22–S23).

Table S24 *Overall* ***Chi-square*** *test results for* ***Substrate × Host plant, with pooled Population and Year data.*** *The table shows the Chi-squared statistics, degrees of freedom, and p-value testing independence between the factors.*

Test	Chi_Sq	DF	P_value
Substrate × Hostplant	147.065	2	0.0000000

Table S25 Cell-level contributions for Substrate × Host plant, with pooled Population and Year data. *Observed and expected counts, as well as standardized residuals, are shown for each combination of factors. Standardized residuals indicate which cells contribute most to the overall Chi-squared statistics. P-value is reported in Table S21.*

Substrate	Hostplant	Observed	Expected	Std_Residual
Land	Gypsywort	97	38.98998	11.649874
Water	Gypsywort	31	89.01002	-11.649874
Land	Marsh woundwort	21	45.08216	-4.530891
Water	Marsh woundwort	127	102.91784	4.530891
Land	Mint	338	371.92786	-4.913251
Water	Mint	883	849.07214	4.913251

Supplementary material for the scientific article: "Predator avoidance behaviour in green tortoise beetles depends on environment and shield ontogeny, but varies over time and space"

Methods and Discussion from Chi-square tests: Substrate × Host plant

Methods: The data were pooled by population and year because the sample sizes were too small when analysed separately. To assess if the occurrence of host plant species differed significantly from expected values under independence, we applied a Pearson's Chi-square test. We examined the Chi-square values, p-values, and the residuals of the test.

Results: There was a significant difference in host plant distribution above water and land in our collected data (supplementary table S24). Gypsywort occurred more on land than on water (supplementary table S25). Mint and marsh woundwort occurred more on water than on land (supplementary table S25).

Discussion:

The adjusted probability of gypsywort (62%, SE = 4%) from the ***"Pooled host plant model"*** *with main effects* (supplementary table S21), significantly deviated from 50% (95% confidence interval does not overlap with 50%, and 50% = no preference to drop or duck), indicating that beetles on this host plant duck in a higher frequency than they drop. Therefore, the higher occurrence of gypsywort on land could affect the anti-predator behavioural response on land towards increased ducking. However, this is not what we see in the results from the ***"best fit model"*** (Result section in MS), in which the adjusted probability to duck on land (44%, SE = 3.0%) did not differ from 50% (50% = no preference to drop or duck).

Furthermore, the adjusted probability of mint (57%, SE = 2%) ***"Pooled host plant model"*** *with main effects* (supplementary table S21) also deviated from 50% (50% = no preference to drop or duck), showing a higher preference of ducking. Since mint was more abundant above water, the preference of the beetles to duck on this host plant aligns with the higher preference of ducking above water (the adjusted probability to duck above water: 62% (SE = 3.5%) from the ***"best fit model"*** in the Result section in MS). Although we cannot fully disentangle the effect of hostplant and substrate in this case, the results (Result section in MS) show that effects of ontogeny and substrate are constant across different models and highly statistically significant compared to the effect of hostplant.

Finally, the adjusted probability of marsh woundwort (48%, SE= 4%) ***"Pooled host plant model"*** *with main effects* (supplementary table S21) did not deviate from 50% (50% = no preference to drop or duck), indicating that there is no preference to either drop or duck for beetles on this host plant. Marsh woundwort occurred more above water, but despite no preference for either behaviour on this host plant, beetles above water still ducked to a higher extent (***"best fit model"*** (Result section in MS), 62% (SE = 3.5%) significantly deviates from 50% (50% = no preference to drop or duck)). This result indicates that the effect of substrate is still evident despite the effect of host plant on antipredator behaviour.

Table S26 *Overall* ***Chi-square*** *test results for* ***Substrate × Ontogeny within Population and Year.*** *The table shows the Chi-squared statistics, degrees of freedom, and p-value testing independence between the factors.*

Population	Year	Test	Chi_Sq	DF	P_value
Björkelunda	2022	Substrate × Ontogeny	0.8914323	2	0.6403655
Häckeberga	2022	Substrate × Ontogeny	7.364999	2	0.02516
Nöbbelöv	2022	Substrate × Ontogeny	3.543335	2	0.1700492
Björkelunda	2023	Substrate × Ontogeny	5.291611	2	0.0709482
Häckeberga	2023	Substrate × Ontogeny	2.837172	2	0.2420561
Nöbbelöv	2023	Substrate × Ontogeny	3.966235	2	0.1376395

Table S27 Cell-level contributions for Substrate × Ontogeny within Population and Year*. Observed and expected counts, as well as standardized residuals, are shown for each combination of factors. Standardized residuals indicate which cells contribute most to the overall Chi-squared statistics. P-values are reported in Table S25.*

**Table S27 (continued)**						
**Population**	**Year**	**Ontogeny**	**Substrate**	**Observed**	**Expected**	**Std_Residual**
Björkelunda	2022	hard-immature	Land	44	40.55528	0.7905268
Björkelunda	2022	hard-mature	Land	37	40.55528	-0.8159003
Björkelunda	2022	soft-immature	Land	50	49.88943	0.0241576
Björkelunda	2022	hard-immature	Water	82	85.44472	-0.7905268
Björkelunda	2022	hard-mature	Water	89	85.44472	0.8159003
Björkelunda	2022	soft-immature	Water	105	105.11057	-0.0241576
Häckeberga	2022	hard-immature	Land	14	7.605333	2.7096920
Häckeberga	2022	hard-mature	Land	8	10.058667	-0.7839987
Häckeberga	2022	soft-immature	Land	24	28.336000	-1.4033696
Häckeberga	2022	hard-immature	Water	48	54.394667	-2.7096920
Häckeberga	2022	hard-mature	Water	74	71.941333	0.7839987
Häckeberga	2022	soft-immature	Water	207	202.664000	1.4033696
Nöbbelöv	2022	hard-immature	Land	53	50.871508	1.0353415
Nöbbelöv	2022	hard-mature	Land	25	28.067039	-1.8222335
Nöbbelöv	2022	soft-immature	Land	79	78.061453	0.4273251
Nöbbelöv	2022	hard-immature	Water	5	7.128492	-1.0353415
Nöbbelöv	2022	hard-mature	Water	7	3.932961	1.8222335
Nöbbelöv	2022	soft-immature	Water	10	10.938548	-0.4273251
Björkelunda	2023	hard-immature	Land	7	8.265306	-0.5226045

Table S27 (continued)

Population	Year	Ontogeny	Substrate	Observed	Expected	Std_Residual
Björkelunda	2023	hard-mature	Land	14	8.396501	2.3003502
Björkelunda	2023	soft-immature	Land	24	28.338192	-1.4368349
Björkelunda	2023	hard-immature	Water	56	54.734694	0.5226045
Björkelunda	2023	hard-mature	Water	50	55.603499	-2.3003502
Björkelunda	2023	soft-immature	Water	192	187.661808	1.4368349
Häckeberga	2023	hard-immature	Land	2	4.693642	-1.5906824
Häckeberga	2023	hard-mature	Land	29	29.167630	-0.0539945
Häckeberga	2023	soft-immature	Land	27	24.138728	0.9348424
Häckeberga	2023	hard-immature	Water	12	9.306358	1.5906824
Häckeberga	2023	hard-mature	Water	58	57.832370	0.0539945
Häckeberga	2023	soft-immature	Water	45	47.861272	-0.9348424
Nöbbelöv	2023	hard-immature	Land	8	6.157895	1.4395153
Nöbbelöv	2023	hard-mature	Land	12	15.052632	-1.7867869
Nöbbelöv	2023	soft-immature	Land	19	17.789474	0.6925489
Nöbbelöv	2023	hard-immature	Water	1	2.842105	-1.4395153
Nöbbelöv	2023	hard-mature	Water	10	6.947368	1.7867869
Nöbbelöv	2023	soft-immature	Water	7	8.210526	-0.6925489

Supplementary material for the scientific article: “Predator avoidance behaviour in green tortoise beetles depends on environment and shield ontogeny, but varies over time and space”

Methods and Discussion from Chi-square tests: Substrate × Ontogeny

Methods: The data were analysed separately per population and year. A Pearson’s Chi-square test was applied to each data table to assess if beetles with different shield ontogeny differed significantly from expected values under independence. We examined the Chi-square values, p-values, and residuals for each test.

Results: Overall, all ontogenetic stages were evenly distributed across the substrates, except in Häckeberga 2022 and Björkelunda 2023 (supplementary table S26). In Häckeberga 2022 hard-immature beetles distributed more above land and in Björkelunda 2023 hard-mature beetles distributed more above land (supplementary table S27).

Discussion:

Hard-mature beetles, which duck to a higher extent (adjusted probability from the **“*best fit model*”:** 71% (SE = 3.1%) significantly deviates from 50% (95% confidence interval does not overlap with 50%, and 50% = no preference to drop or duck (Result section in MS)), does not distribute mainly above water, on which we find a higher probability of ducking (the adjusted probability to duck above water 62% (SE = 3.5%) from the **“*best fit model”*** significantly deviates from 50% (Result section in MS)).

Furthermore, the soft-immature beetles, that ducks less (from the **“*best fit model”*** , adjusted probability 43% (SE = 2.9%) significantly deviate from 50%, 50% = no preference to drop or duck (Result section in MS)), were not mainly distributed above land.

These results may therefore support that beetles of all ontogenetic life stages in our study populations duck more above water.
