# Supplementary material for: Predator avoidance behaviour in green tortoise beetles depends on environment and shield ontogeny, but varies over time and space
Source: Oecologia. 2026 Nov 11;208(12):169. doi: 10.1007/s00442-026-06005-1 (PMC13751549; doi:10.1007/s00442-026-06005-1)
Supplement: Supplementary file 3 [file 442_2026_6005_MOESM2_ESM.docx]

Supplementary material (R-code) for the scientific article: “Predator avoidance behaviour in green tortoise beetles depends on environment and shield ontogeny, but varies over time and space”

Authors: Erica Winslott*, Kristina Karlsson Green, Maja Tarka

*Corresponding author: erica.winslott@biol.lu.se

\# R-packages

\## Data structuring

library(dplyr)	# Tools for data manipulation (e.g., filter, select, mutate)

library(tidyr) # Functions for reshaping and tidying data (e.g., pivot, separate)

library(stringr)	# Consistent and simple string handling

library(tibble)	# Tidyverse-friendly data frames with enhanced printing

\## Data analysis and diagnostics

library(car)	# Regression diagnostics and utilities (e.g., VIF, recode)

library(MuMIn)	# Model selection and multi-model inference

library(emmeans)	# Estimated marginal means (least-squares means) analysis

\# Comparing Models with Walk as Duck, and Walk removed from analyses.

\## Models with main effects.

\### Walk = Duck

rm(list=ls())

setwd("C:/XXX")

Data <- read.csv("Data.csv", header = T, row.names=NULL, sep = ",")

\# Walk = Duck

Data$Drop.or.Duck <- replace(Data$Drop.or.Duck, Data["Drop.or.Duck"]== "Walk", "Duck")

\# Make data binomial

Data$Drop.or.Duck <- replace(Data$Drop.or.Duck, Data["Drop.or.Duck"]== "Duck", "1")

Data$Drop.or.Duck <- replace(Data$Drop.or.Duck, Data["Drop.or.Duck"]== "Drop", "0")

\# Data structure

Data$Drop.or.Duck <- factor(Data$Drop.or.Duck)

Data$Substrate <- factor(Data$Substrate)

Data$Population <- factor(Data$Population)

Data$Ontogeny <- factor(Data$Ontogeny)

Data$Year <- factor(Data$Year)

Data$Hostplant <- factor(Data$Hostplant)

Model1 <- glm(Drop.or.Duck~Substrate+Ontogeny+Population+Year,data = Data, family = binomial)

Anova(Model1, test="LR")

\### Walk = Removed

rm(list=ls())

setwd("C:/XXX")

Data <- read.csv("Data.csv", header = T, row.names=NULL, sep = ",")

\# Removing walk

Data$Drop.or.Duck <- replace(Data$Drop.or.Duck, Data["Drop.or.Duck"]== "Walk", "NA")

Data <- Data %>% filter(Drop.or.Duck != "NA")

\# Make data binomial

Data$Drop.or.Duck <- replace(Data$Drop.or.Duck, Data["Drop.or.Duck"]== "Duck", "1")

Data$Drop.or.Duck <- replace(Data$Drop.or.Duck, Data["Drop.or.Duck"]== "Drop", "0")

\# Data structure

Data$Drop.or.Duck <- factor(Data$Drop.or.Duck)

Data$Substrate <- factor(Data$Substrate)

Data$Population <- factor(Data$Population)

Data$Ontogeny <- factor(Data$Ontogeny)

Data$Year <- factor(Data$Year)

Model2 <- glm(Drop.or.Duck~Substrate+Ontogeny+Population+Year,data = Data, family = binomial)

Anova(Model2, test="LR")

\## Models with main effects and all interactions.

\### Walk = Duck

rm(list=ls())

setwd("C:/XXX")

Data <- read.csv("Data.csv", header = T, row.names=NULL, sep = ",")

\# Walk = Duck

Data$Drop.or.Duck <- replace(Data$Drop.or.Duck, Data["Drop.or.Duck"]== "Walk", "Duck")

\# Make data binomial

Data$Drop.or.Duck <- replace(Data$Drop.or.Duck, Data["Drop.or.Duck"]== "Duck", "1")

Data$Drop.or.Duck <- replace(Data$Drop.or.Duck, Data["Drop.or.Duck"]== "Drop", "0")

\# Data structure

Data$Drop.or.Duck <- factor(Data$Drop.or.Duck)

Data$Substrate <- factor(Data$Substrate)

Data$Population <- factor(Data$Population)

Data$Ontogeny <- factor(Data$Ontogeny)

Data$Year <- factor(Data$Year)

Model3 <- glm(Drop.or.Duck~Substrate*Ontogeny*Population*Year,data = Data, family = binomial)

Anova(Model3, test="LR")

\### Walk = Removed

rm(list=ls())

setwd("C:/XXX")

Data <- read.csv("Data.csv", header = T, row.names=NULL, sep = ",")

\# Removing walk

Data$Drop.or.Duck <- replace(Data$Drop.or.Duck, Data["Drop.or.Duck"]== "Walk", "NA")

Data <- Data %>% filter(Drop.or.Duck != "NA")

\# Make data binomial

Data$Drop.or.Duck <- replace(Data$Drop.or.Duck, Data["Drop.or.Duck"]== "Duck", "1")

Data$Drop.or.Duck <- replace(Data$Drop.or.Duck, Data["Drop.or.Duck"]== "Drop", "0")

Data$Drop.or.Duck <- factor(Data$Drop.or.Duck)

Data$Substrate <- factor(Data$Substrate)

Data$Population <- factor(Data$Population)

Data$Ontogeny <- factor(Data$Ontogeny)

Data$Year <- factor(Data$Year)

Model4 <- glm(Drop.or.Duck~Substrate*Ontogeny*Population*Year,data = Data, family = binomial)

Anova(Model4, test="LR")

\# Data Analyses with Walk = Duck

\## Backward elimination and Akaike Information Criterion (AIC)

\### Backward elimination

rm(list=ls())

setwd("C:/XXX")

Data <- read.csv("Data.csv", header = T, row.names=NULL, sep = ",")

\# Walk = Duck

Data$Drop.or.Duck <- replace(Data$Drop.or.Duck, Data["Drop.or.Duck"]== "Walk", "Duck")

\# Make data binomial

Data$Drop.or.Duck <- replace(Data$Drop.or.Duck, Data["Drop.or.Duck"]== "Duck", "1")

Data$Drop.or.Duck <- replace(Data$Drop.or.Duck, Data["Drop.or.Duck"]== "Drop", "0")

\# Data structure

Data$Drop.or.Duck <- factor(Data$Drop.or.Duck)

Data$Substrate <- factor(Data$Substrate)

Data$Population <- factor(Data$Population)

Data$Ontogeny <- factor(Data$Ontogeny)

Data$Year <- factor(Data$Year)

ModelA <- glm(Drop.or.Duck~Substrate*Ontogeny*Population*Year,data = Data, family = binomial)

Anova(ModelA, test="LR")

ModelB <- glm(Drop.or.Duck~Substrate+Ontogeny+Population+Year+

Substrate*Ontogeny+Substrate*Population+Ontogeny*Population+Substrate*Year+Ontogeny*Year+Population*Year+

Substrate*Ontogeny*Population+Substrate*Ontogeny*Year+Substrate*Population*Year+Ontogeny*Population*Year+

Substrate*Ontogeny*Population*Year,data = Data, family = binomial)

Anova(ModelB, test="LR")

\## Model A = B

\# Remove four way interaction: Substrate*Ontogeny*Population*Year

ModelC <- glm(Drop.or.Duck~Substrate+Ontogeny+Population+Year+

Substrate*Ontogeny+Substrate*Population+Ontogeny*Population+Substrate*Year+Ontogeny*Year+Population*Year+

Substrate*Ontogeny*Population+Substrate*Ontogeny*Year+Substrate*Population*Year+Ontogeny*Population*Year,

data = Data, family = binomial)

Anova(ModelC, test="LR")

\# Remove three way interaction: Substrate*Ontogeny*Population

ModelD <- glm(Drop.or.Duck~Substrate+Ontogeny+Population+Year+

Substrate*Ontogeny+Substrate*Population+Ontogeny*Population+Substrate*Year+Ontogeny*Year+Population*Year+

Substrate*Ontogeny*Year+Substrate*Population*Year+Ontogeny*Population*Year,

data = Data, family = binomial)

Anova(ModelD, test="LR")

\# Remove three way interaction: Substrate*Ontogeny*Year

ModelE <- glm(Drop.or.Duck~Substrate+Ontogeny+Population+Year+

Substrate*Ontogeny+Substrate*Population+Ontogeny*Population+Substrate*Year+Ontogeny*Year+Population*Year+

Substrate*Population*Year+Ontogeny*Population*Year,

data = Data, family = binomial)

Anova(ModelE, test="LR")

\### Model E best Model

\### Akaike Information Criterion (AIC)

rm(list=ls())

setwd("C:/XXX")

Data <- read.csv("Data.csv", header = T, row.names=NULL, sep = ",")

\# Walk = Duck

Data$Drop.or.Duck <- replace(Data$Drop.or.Duck, Data["Drop.or.Duck"]== "Walk", "Duck")

\# Make data binomial

Data$Drop.or.Duck <- replace(Data$Drop.or.Duck, Data["Drop.or.Duck"]== "Duck", "1")

Data$Drop.or.Duck <- replace(Data$Drop.or.Duck, Data["Drop.or.Duck"]== "Drop", "0")

\# Data structure

Data$Drop.or.Duck <- factor(Data$Drop.or.Duck)

Data$Substrate <- factor(Data$Substrate)

Data$Population <- factor(Data$Population)

Data$Ontogeny <- factor(Data$Ontogeny)

Data$Year <- factor(Data$Year)

ModelX <- glm(Drop.or.Duck~Substrate*Ontogeny*Population*Year,data = Data, family = binomial)

options(na.action = "na.fail")

AICX <- dredge(ModelX, rank=AIC)

opts <- options(knitr.kable.NA = "")

AICX[1:10,]

ModelZ <- glm(Drop.or.Duck~Population+Ontogeny+Substrate+Year+

Population*Ontogeny+Population*Year+Ontogeny*Substrate+Ontogeny*Year+

Population*Ontogeny*Year,data = Data, family = binomial)

options(na.action = "na.fail")

AICZ <- dredge(ModelZ, rank=AIC)

opts <- options(knitr.kable.NA = "")

AICZ[1:10,]

\## Since the difference in AIC is less than 2 between the first rows, the second one is chosen due to most factors included.

Anova(ModelZ, test="LR")

\## Best Model: Model E from Backward elimination

\### Anova

rm(list=ls())

setwd("C:/XXX")

Data <- read.csv("Data.csv", header = T, row.names=NULL, sep = ",")

\# Walk = Duck

Data$Drop.or.Duck <- replace(Data$Drop.or.Duck, Data["Drop.or.Duck"]== "Walk", "Duck")

\# Make data binomial

Data$Drop.or.Duck <- replace(Data$Drop.or.Duck, Data["Drop.or.Duck"]== "Duck", "1")

Data$Drop.or.Duck <- replace(Data$Drop.or.Duck, Data["Drop.or.Duck"]== "Drop", "0")

\# Data structure

Data$Drop.or.Duck <- factor(Data$Drop.or.Duck)

Data$Substrate <- factor(Data$Substrate)

Data$Population <- factor(Data$Population)

Data$Ontogeny <- factor(Data$Ontogeny)

Data$Year <- factor(Data$Year)

Data$Hostplant <- factor(Data$Hostplant)

ModelE <- glm(Drop.or.Duck~Substrate+Ontogeny+Population+Year+

Substrate*Ontogeny+Substrate*Population+Ontogeny*Population+Substrate*Year+Ontogeny*Year+Population*Year+

Substrate*Population*Year+Ontogeny*Population*Year,

data = Data, family = binomial)

Anova(ModelE, test="LR")

\### Post Hoc test on Main effects

\#### Ontogeny

\# Compute estimated marginal means for Ontogeny

emm <- emmeans(ModelE, ~Ontogeny)

\# Perform pairwise comparisons

pairwise_comps <- contrast(emm, method = "pairwise")

\# Print the results

summary(pairwise_comps)

\#### Substrate

\# Compute estimated marginal means for Population

emm <- emmeans(ModelE, ~Substrate)

\# Perform pairwise comparisons

pairwise_comps <- contrast(emm, method = "pairwise")

\# Print the results

summary(pairwise_comps)

\#### Population

\# Compute estimated marginal means for Population

emm <- emmeans(ModelE, ~Population)

\# Perform pairwise comparisons

pairwise_comps <- contrast(emm, method = "pairwise")

\# Print the results

summary(pairwise_comps)

\### Effect sizes of Main effects

\#### Ontogeny

emm <- emmeans(ModelE, ~Ontogeny, type="response")

summary(emm)

\#### Substrate

emm <- emmeans(ModelE, ~Substrate, type="response")

summary(emm)

\#### Population

emm <- emmeans(ModelE, ~Population, type="response")

summary(emm)

\### Post Hoc test on interactions

\#### Ontogeny x Population x Year

\# Compute estimated marginal means for Ontogeny*Population*Year

emm <- emmeans(ModelE, ~Ontogeny*Population*Year)

\# Perform pairwise comparisons

pairwise_comps <- contrast(emm, method = "pairwise")

summary(pairwise_comps)

\#### Substrate x Population x Year

\# Compute estimated marginal means for Substrate*Population*Year

emm <- emmeans(ModelE, ~Substrate*Population*Year)

\# Perform pairwise comparisons

pairwise_comps <- contrast(emm, method = "pairwise")

summary(pairwise_comps)

\#### Population x Year

\# Compute estimated marginal means for Population*Year

emm <- emmeans(ModelE, ~Population*Year)

\# Perform pairwise comparisons

pairwise_comps <- contrast(emm, method = "pairwise")

summary(pairwise_comps)

\#### Ontogeny x Population

\# Compute estimated marginal means for Ontogeny*Population

emm <- emmeans(ModelE, ~Ontogeny*Population)

\# Perform pairwise comparisons

pairwise_comps <- contrast(emm, method = "pairwise")

summary(pairwise_comps)

\#### Substrate x Ontogeny

\# Compute estimated marginal means for Substrate*Ontogeny

emm <- emmeans(ModelE, ~Substrate*Ontogeny)

\# Perform pairwise comparisons

pairwise_comps <- contrast(emm, method = "pairwise")

summary(pairwise_comps)

\### Effect sizes of interactions

\#### Ontogeny x Population x Year

emm <- emmeans(ModelE, ~Ontogeny*Population*Year, type="response")

summary(emm)

\#### Substrate x Population x Year

emm <- emmeans(ModelE, ~Substrate*Population*Year, type="response")

summary(emm)

\#### Population x Year

emm <- emmeans(ModelE, ~Population*Year, type="response")

summary(emm)

\#### Ontogeny x Population

emm <- emmeans(ModelE, ~Ontogeny*Population, type="response")

summary(emm)

\#### Substrate x Ontogeny

emm <- emmeans(ModelE, ~Substrate*Ontogeny, type="response")

summary(emm)

\# Chi-Square test - Distribution of beetles on land and water

\## Populations - 2022

\# Björkelunda

Bjork22 <- Data %>% filter(Population == "Björkelunda") %>% filter(Year == "2022")

T_B <- table(Bjork22$Ontogeny, Bjork22$Substrate)

Chitest <- chisq.test(T_B)

Chitest$stdres

Chitest

\# Häckeberga

Hacke22 <- Data %>% filter(Population == "Häckeberga") %>% filter(Year == "2022")

T_H <- table(Hacke22$Ontogeny, Hacke22$Substrate)

Chitest <- chisq.test(T_H)

Chitest$stdres

Chitest

\# Nöbbelöv

Nobbe22 <- Data %>% filter(Population == "Nöbbelöv") %>% filter(Year == "2022")

T_N <- table(Nobbe22$Ontogeny, Nobbe22$Substrate)

Chitest <- chisq.test(T_N)

Chitest$stdres

Chitest

\## Populations - 2023

\# Björkelunda

Bjork23 <- Data %>% filter(Population == "Björkelunda") %>% filter(Year == "2023")

T_B <- table(Bjork23$Ontogeny, Bjork23$Substrate)

Chitest <- chisq.test(T_B)

Chitest$stdres

Chitest

\# Häckeberga

Hacke23 <- Data %>% filter(Population == "Häckeberga") %>% filter(Year == "2023")

T_H <- table(Hacke23$Ontogeny, Hacke23$Substrate)

Chitest <- chisq.test(T_H)

Chitest$stdres

Chitest

\# Nöbbelöv

Nobbe23 <- Data %>% filter(Population == "Nöbbelöv") %>% filter(Year == "2023")

T_N <- table(Nobbe23$Ontogeny, Nobbe23$Substrate)

Chitest <- chisq.test(T_N)

Chitest$stdres

Chitest

\# Additional GLM with Ontogeny, Substrate, and Host plant species as fixed effects

\# Remove Host plant species = NA

Data <- Data %>%

filter(!is.na(Hostplant))

\## GLM

\### Substrate x Hostplant x Ontogeny

Model1 <- glm(Drop.or.Duck~Substrate*Hostplant*Ontogeny,data = Data, family = binomial)

Anova(Model1, test="LR")

\### Substrate + Hostplant + Ontogeny + Substrate x Hostplant + Substrate x Ontogeny + Hostplant x Ontogeny

Model2 <- glm(Drop.or.Duck~Substrate+Hostplant+Ontogeny +

Substrate*Hostplant + Substrate*Ontogeny + Hostplant * Ontogeny ,data = Data, family = binomial)

Anova(Model2, test="LR")

\### Substrate + Hostplant + Ontogeny + Substrate x Hostplant + Hostplant x Ontogeny

Model3 <- glm(Drop.or.Duck~Substrate+Hostplant+Ontogeny +

Substrate*Hostplant + Hostplant * Ontogeny ,data = Data, family = binomial)

Anova(Model3, test="LR")

\### Substrate + Hostplant + Ontogeny + Substrate x Hostplant

Model4 <- glm(Drop.or.Duck~Substrate+Hostplant+Ontogeny +

Substrate*Hostplant ,data = Data, family = binomial)

Anova(Model4, test="LR")

\### Substrate + Hostplant + Ontogeny

Model5 <- glm(Drop.or.Duck~Substrate+Hostplant+Ontogeny ,data = Data, family = binomial)

Anova(Model5, test="LR")

\## Effect sizes of Host plant species

emm <- emmeans(Model5, ~Hostplant, type="response")

summary(emm)

\## Post Hoc test of Host plant species

\# Compute estimated marginal means for Hostplant

emm <- emmeans(Model5, ~Hostplant)

\# Perform pairwise comparisons

pairwise_comps <- contrast(emm, method = "pairwise")

\# Print the results

summary(pairwise_comps)

\# Chi-Square tests

\## Distribution of ontogenetic stages on Host plant species

Table_OnT <- table(Data$Hostplant, Data$Ontogeny)

Chitest <- chisq.test(Table_OnT)

Chitest$stdres

Chitest

\## Occurrence of Host plant species above substrates

Table_LoW <- table(Data$Substrate, Data$Hostplant)

Chitest <- chisq.test(Table_LoW)

Chitest$stdres

Chitest
